# Cooperative CO_2_ adsorption mechanism in a perfluorinated Ce^IV^-based metal organic framework†

**DOI:** 10.1039/d2ta09746j

**Published:** 2023-02-14

**Authors:** Margherita Cavallo, Cesare Atzori, Matteo Signorile, Ferdinando Costantino, Diletta Morelli Venturi, Athanasios Koutsianos, Kirill A. Lomachenko, Lucia Calucci, Francesca Martini, Andrea Giovanelli, Marco Geppi, Valentina Crocellà, Marco Taddei

**Affiliations:** a Dipartimento di Chimica, Centro di Riferimento NIS e INSTM, Università di Torino *Via* G. Quarello 15, I-10135 and *Via* P. Giuria 7 I-10125 Torino Italy valentina.crocella@unito.it; b European Synchrotron Radiation Facility 71 Avenue des Martyrs, CS 40220 38043 Grenoble Cedex 9 France; c Dipartimento di Chimica, Biologia e Biotecnologie, Unità di Ricerca INSTM, Università di Perugia Via Elce di Sotto 8 06123 Perugia Italy; d Centre for Research & Technology Hellas/Chemical Process and Energy Resources Institute 6th km. Charilaou-Thermis 57001 Greece; e Istituto di Chimica dei Composti Organo Metallici, Unità di Ricerca INSTM, Consiglio Nazionale delle Ricerche Via Giuseppe Moruzzi 1 56124 Pisa Italy; f Centro per l’Integrazione della Strumentazione Scientifica dell’Università di Pisa (CISUP) 56126 Pisa Italy; g Dipartimento di Chimica e Chimica Industriale, Unità di Ricerca INSTM, Università di Pisa Via Giuseppe Moruzzi 13 56124 Pisa Italy marco.taddei@unipi.it; h Energy Safety Research Institute, Swansea University Fabian Way Swansea SA1 8EN UK

## Abstract

Adsorbents able to uptake large amounts of gases within a narrow range of pressure, *i.e.*, phase-change adsorbents, are emerging as highly interesting systems to achieve excellent gas separation performances with little energy input for regeneration. A recently discovered phase-change metal–organic framework (MOF) adsorbent is F4_MIL-140A(Ce), based on Ce^IV^ and tetrafluoroterephthalate. This MOF displays a non-hysteretic step-shaped CO_2_ adsorption isotherm, reaching saturation in conditions of temperature and pressure compatible with real life application in post-combustion carbon capture, biogas upgrading and acetylene purification. Such peculiar behaviour is responsible for the exceptional CO_2_/N_2_ selectivity and reverse CO_2_/C_2_H_2_ selectivity of F4_MIL-140A(Ce). Here, we combine data obtained from a wide pool of characterisation techniques – namely gas sorption analysis, *in situ* infrared spectroscopy, *in situ* powder X-ray diffraction, *in situ* X-ray absorption spectroscopy, multinuclear solid state nuclear magnetic resonance spectroscopy and adsorption microcalorimetry – with periodic density functional theory simulations to provide evidence for the existence of a unique cooperative CO_2_ adsorption mechanism in F4_MIL-140A(Ce). Such mechanism involves the concerted rotation of perfluorinated aromatic rings when a threshold partial pressure of CO_2_ is reached, opening the gate towards an adsorption site where CO_2_ interacts with both open metal sites and the fluorine atoms of the linker.

## Abstract 2

The peculiar mechanism of cooperative CO_2_ adsorption in a perfluorinated Ce^IV^-based metal–organic framework with MIL-140 topology is disclosed using a combination of experimental and computational methods.

## Graphical abstract

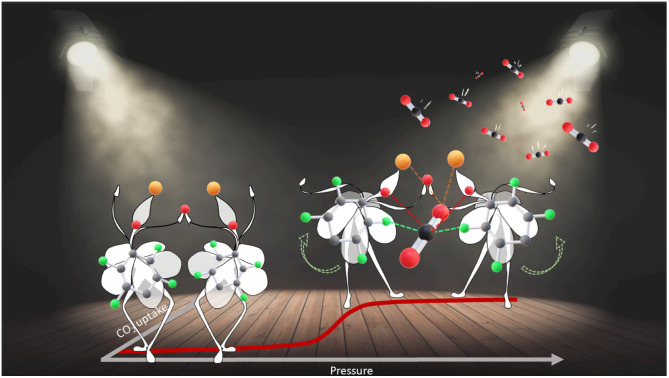

## Introduction

Solid adsorbents for gas separations are the object of intensive research, due to their potential advantages, in terms of energy intensity and recyclability, over state-of-the-art technologies based on either cryogenic distillation or absorption/scrubbing.^1–5^ Metal–organic frameworks (MOFs) are among the candidates investigated to this end, alongside zeolites, activated carbons and amine-functionalised porous silicas.^3,4,6,7^ If compared to the other classes of adsorbents, MOFs offer superior structural control at the atomic level and virtually unlimited possibilities for functionalisation and tuning of the physicochemical properties.^1^ One of the most intriguing aspects of MOF adsorbents is that they can display unique adsorption phenomena, associated with either highly specific interaction of the adsorbate with the surface, structural flexibility, or combinations thereof.^8–16^

So-called “phase-change” MOFs are of particular interest for gas separations because they display steep gas uptake when a threshold partial pressure of a given adsorbate is reached, with potential benefits in terms of achievable working capacity, selectivity and energy efficiency.^17,18^ This behaviour is determined by an adsorption-induced structural rearrangement, which can be either (i) a breathing/swelling effect, due to the flexible nature of the framework, which leads to significant variations in the volume of the unit cell;^9,19–22^ (ii) a cooperative adsorption mechanism, such as the CO_2_ insertion occurring in so-called amine-appended MOFs, which involves changes in the coordination environment of the metal and highly specific hydrogen bonding interactions;^18,23,24^ or (iii) the concerted rotation of organic rings within the framework, which results in a ring configuration that maximises the host–guest interaction.^25–27^

**Fig. 1:**
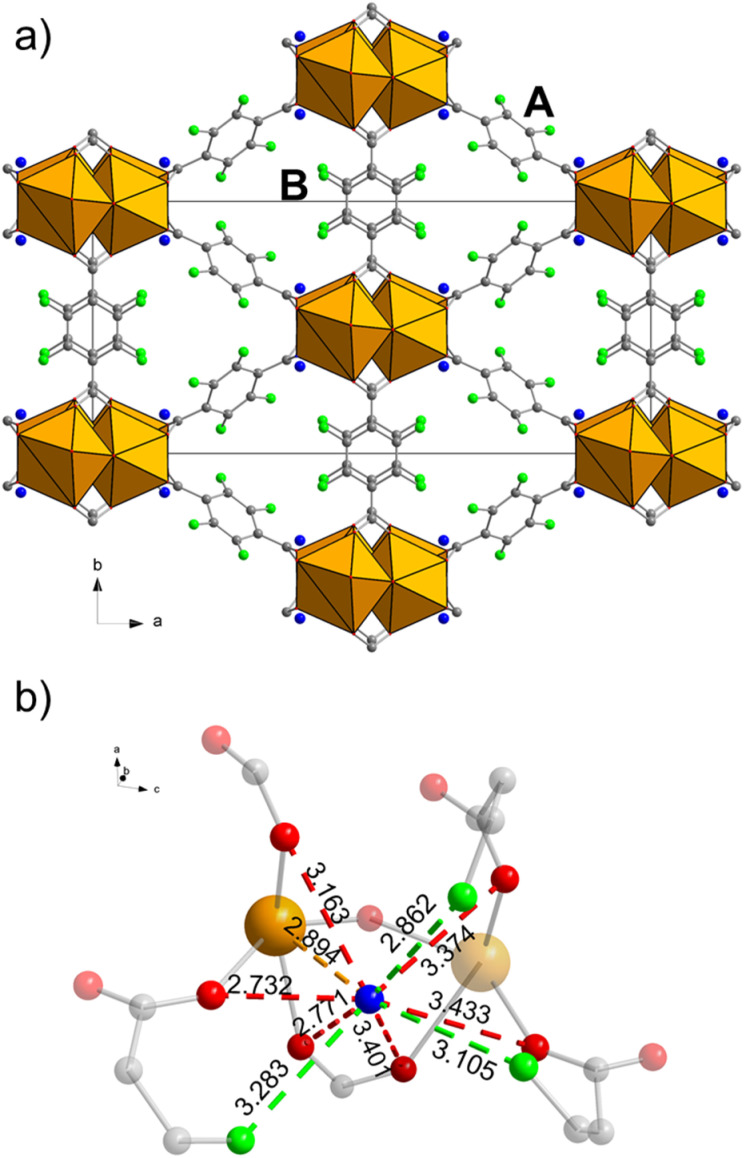
Crystal structure of as-synthesised F4_MIL-140A(Ce) viewed along the crystallographic *c* axis (a) and local environment around the water molecule (b). In (a) the two crystallographically independent linkers sitting on an inversion centre and on a two-fold axis are identified with A and B, respectively. In (b) a threshold distance of 3.5 Å between the O atom of water and surrounding atoms was chosen to identify possible interactions, represented as dashed lines having the same colour of the interacting atom. Non-interacting atoms in (b) are shaded. Colour code: Ce, orange; F, green; C, grey; O, red; H_2_O, blue. H atoms are not shown because the structure was determined from PXRD data.

Some of us recently reported the discovery of F4_MIL-140A(Ce), an ultramicroporous MOF based on Ce^IV^ and tetrafluoroterephthalate (Fig. 1).^28^ F4_MIL-140A(Ce) is built from the connection of one-dimensional inorganic building units, made up of octacoordinated Ce^IV^ ions, carboxylate groups belonging to the linker and μ_3_-O species, *via* the perfluorinated aromatic rings of the linker. This structure features narrow triangular channel-like pores lined with the fluorine atoms belonging to the linkers (Fig. 1a). Different from its Zr^IV^-based MIL-140 analogues,^29^ F4_MIL-140A(Ce) contains one water molecule per Ce atom, located in the proximity of the metal atom and at distances compatible with a number of non-covalent interactions with the organic linker (Fig. 1b).

F4_MIL-140A(Ce) displays a non-hysteretic step-shaped CO_2_ adsorption isotherm, with steep uptake increase at pressure <0.2 bar at 298 K, leading to reach saturation within a narrow range of pressure.^28^ Such behaviour is not observed when the MOF is exposed to other gases, *e.g.*, N_2_ or C_2_H_2_, endowing this material with high CO_2_/N_2_ selectivity and with reverse CO_2_/C_2_H_2_ selectivity, a highly sought-after feature.^30^ Furthermore, this MOF can be synthesised from commercially available reagents in mild conditions and in aqueous medium, an attractive method for prospective bulk production.^31^ Considering such a unique combination of features, we believe that understanding the adsorption mechanism in F4_MIL-140A(Ce) becomes an issue of primary concern. Besides the fundamental interest in describing a potentially novel phenomenon, the knowledge deriving from such an endeavour might help to engineer new materials with the same working principle and improved separation performance.

A mechanism was in fact recently proposed based on powder X-ray diffraction (PXRD) data, infrared (IR) spectroscopy data, and density functional theory (DFT) simulations, claiming that CO_2_ adsorbs in a site located among three fluorinated rings, where the Lewis acidic C atom interacts with four electron-rich F atoms,^30^ akin to what observed in SIFSIX-3 materials^32,33^ and in fluorinated bis(pyrazolyl)-based MOFs.^34^ In the present work, we propose an alternative mechanism, deduced from the combination of data obtained from a wide pool of characterisation techniques, including gas sorption analysis, *in situ* IR spectroscopy, *in situ* PXRD, *in situ* X-ray absorption spectroscopy (XAS) at the Ce K-edge, ^1^H, ^13^C and ^19^F solid state nuclear magnetic resonance (SSNMR) spectroscopy and adsorption microcalorimetry, with periodic DFT simulations.

## Results and discussion

### Adsorption properties

#### Gas adsorption volumetry

**Fig. 2:**
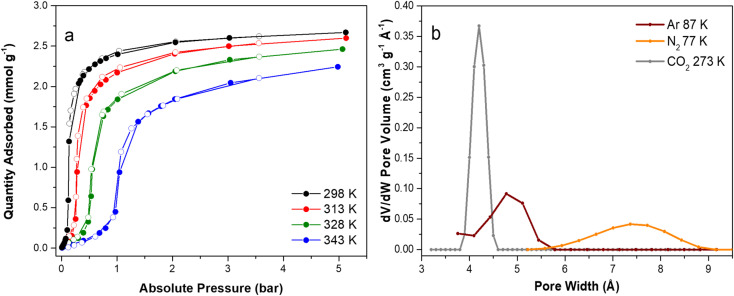
CO_2_ adsorption (filled circles) and desorption (empty circles) isotherms collected on F4_MIL-140A(Ce) at 298 K (black), 313 K (red), 328 K (green) and 343 K (blue) up to 5 bar (a) and pore size distributions of F4_MIL-140A(Ce) computed by NL-DFT of: Ar at 87 K (dark red), N_2_ at 77 K (orange) and CO_2_ at 273 K (grey) (b).

CO_2_ adsorption/desorption volumetric isotherms were collected at 298, 313, 328 and 343 K in the 0–5 bar pressure range to study the pure CO_2_ adsorption capacity of the material (Fig. 2a). All the isotherms exhibit a peculiar step, which moves to progressively higher pressure by increasing the temperature and the pressure range over which saturation occurs becomes broader. The maximum amount of adsorbed CO_2_ on F4_MIL-140A(Ce) is around 2.6 mmol g^−1^ at 298 K and 5 bar and shows the usual decrease by increasing the temperature (2.2 mmol g^−1^ at 343 K and 5 bar). The isotherms were fitted with a dual site Langmuir-Freundlich model (Fig. S1–S4†) and the pressure at which they display the maximum slope (*P*_max_) was determined from the first derivative curve. Plotting log *P*_max_*versus* temperature, a linear trend emerges between 298 and 343 K, in principle allowing to predict which pressure the step will take place at within the investigated temperature range (Fig. S5†). The isosteric heat of adsorption (*Q*_st_), extracted from the fitted isotherms using the linear version of the Clausius–Clapeyron equation (Fig. S6†), ranges between 35 and 45 kJ mol^−1^.

The sharp uptake of gas (*i.e.* the isotherm step) moves to higher pressures by increasing the temperature. This peculiar adsorption feature allows easily adapting the phase transition mechanism of this MOF to the partial pressure of common post-combustion streams. Additionally, this characteristic isotherm is known to be particularly suitable for adsorption processes thanks to the large working capacity that can be achieved upon small swings in temperature and/or pressure. Such step-shaped isotherms have previously been observed in other MOFs exhibiting higher CO_2_ adsorption capacities at saturation, as in the amine-appended Mg_2_(dobpdc)^18^ (3.4 mmol g^−1^ at 298 K and 5 bar) and in ELM-11 (ref. 9) (3.6 mmol g^−1^ at 273 K and 0.3 bar). However, in the former case, the material displays a much higher isosteric heat of CO_2_ adsorption (around −70 kJ mol^−1^), whereas in the latter the phase transition occurs in a higher pressure range (300 mbar at 273 K). This leads, on the one hand, to stronger interaction energies and, consequently, to higher regeneration energies, on the other hand, to a low adsorption of CO_2_ at low pressures. To further investigate the peculiar adsorption mechanism of F4_MIL-140A(Ce), the textural properties were accurately studied by collecting low pressure adsorption/desorption volumetric isotherms using three different adsorptives: N_2_ at 77 K, Ar at 87 K and CO_2_ at 273 K (Fig. S7† in the ESI†). The isotherms were measured consecutively using the same MOF powder. Therefore, the differences observed with the selected probe molecules can be attributed to the nature of the probe molecule at the analysis temperature. Ar and N_2_ show the typical type I(a) isotherm characteristic of microporous materials, while CO_2_ exhibits the less common step-shaped isotherm specific of the so-called “phase change” adsorbents (Fig. S7a† in the 0 < *p*/*p*^0^ < 0.03 range and Fig. S7b† in the whole *p*/*p*^0^ range), in agreement with the previous results reported by D'Amato *et al.*^28^ The semi-logarithmic isotherms reported in the inset of Fig. S7a† show that the pore filling with Ar occurs at higher relative pressure regions compared to N_2_. The difference in relative pressures becomes more significant if the MOF possesses open metal sites, highlighting the advantage of using monoatomic inert Ar for the characterization of ultramicroporous MOFs. The BET area values (Table S2 and Fig. S8–S10†) display an incremental trend, suggesting a different interaction of the molecules with the material (Table S2†). The BET area obtained by Ar isotherm (213.8 m^2^ g^−1^) is lower compared to N_2_ (244.8 m^2^ g^−1^). The spherical Ar molecules display a more ideal packing on the surface compared to diatomic N_2_ molecules, whose orientation on the surface can be affected by the possible presence of open metal sites able to interact with N_2_ itself. As a consequence, an uncertainty in the evaluation of the cross-sectional area of nitrogen gas is very likely, resulting in an overestimation of the computed BET area with this probe.^35,36^ The highest BET area value is the one derived from the CO_2_ isotherm at 273 K (262 m^2^ g^−1^), suggesting that a structural rearrangement might occur when F4_MIL-140A(Ce) interacts with CO_2_, as highlighted by the peculiar step-shaped isotherm collected by using this molecule as adsorptive (Fig. S7†).

The pore size distribution (PSD) was also evaluated by applying the non-local density functional theory (NL-DFT) to the adsorption isotherms. It is well-known that the PSD computed by NL-DFT methods depends on the selected kernel of isotherms. However, despite the high number of MOF structures reported to date, proper DFT kernels to study the unique pore size and shapes of these microporous materials are still missing.^36^ For this reason, the PSD data for MOFs should be considered as merely indicative. Moreover, the goodness of the PSD obtained by DFT depends on the goodness of the fitting of the experimental isotherm (see Fig. S11, S12 and S13†). In all the investigated cases, the fit between the calculated and the experimental isotherms was imperfect (the best fit is the one obtained with the N_2_ isotherm). Again, this suggests that PSD data should be merely considered as a possible (and not unquestionable) description of the intrinsic porosity of the investigated MOF. The PSDs computed from the three molecular probes are compared in Fig. 2b and Table S1.† N_2_, a non-ideal molecule for the analysis of microporous materials, gives the highest value (∼7.4 Å), while Ar and CO_2_ PSDs show the presence of a family of ultramicropores of around 4.8 and 4.2 Å diameter, respectively, values in better agreement with that estimated on the basis of the crystal structure of as-synthesised F4_MIL-140A(Ce) (between 4.8 and 5.8 Å, measured from the atomic centres).^28^ The result obtained with N_2_ does not surprise, indeed it is known that this probe can significantly overestimate the PSD of MOFs with very small micropores, especially if the pore dimension is close to the kinetic diameter of the molecule (3.64 Å for N_2_).^36^ The micropore size obtained by both Ar and CO_2_ is more reliable and the PSD derived from CO_2_ at 273 K is very narrow. This could be a further proof of a possible structural rearrangement of the material in the presence of CO_2_, which allows the detection of the actual micropores size just with an adsorptive inducing a “phase change” adsorption. Still, it is worth noting that N_2_ and Ar show a compatible micropore volume (Fig. S14†) while CO_2_ displays a higher value, which might be seen as a further proof of a structural transition caused by this molecule.

#### *In situ* IR spectroscopy

**Fig. 3:**
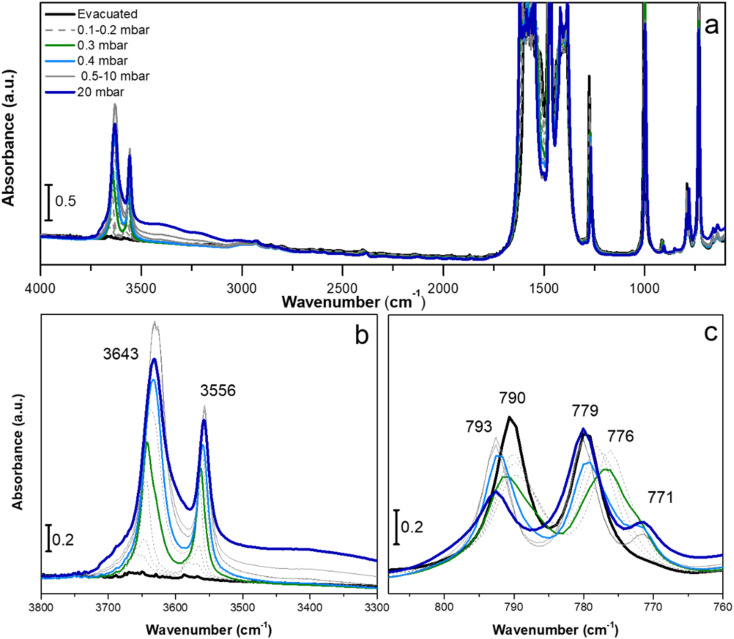
IR spectra of F4_MIL-140A(Ce) collected dosing 0.1–20 mbar of H_2_O on the evacuated sample, reported in the full spectral range (a), and in the 3800–3300 cm^−1^ (b) and 810–760 cm^−1^ (c) regions.

As mentioned in the Introduction, a notable feature of as-synthesised F4_MIL-140A(Ce) is that it contains one H_2_O molecule per formula unit, located in the proximity of a Ce atom.^28^ Hydrogen bonding-like interactions are also likely to exist with F atoms and carboxylate groups from the surrounding organic linkers (Fig. 1b). According to the thermogravimetric curve (Fig. S15†), removal of H_2_O is completed at a temperature as high as 423 K, suggesting that it is strongly adsorbed. The evacuation process of F4_MIL-140A(Ce) was further investigated using *in situ* IR spectroscopy (Fig. 3). Activation at 393 K for 12 h under vacuum was effective to remove the adsorbed H_2_O before the spectroscopic experiments (see black spectrum in Fig. 3). The evacuated F4_MIL-140A(Ce) was then exposed to increasing pressures of H_2_O, observing significant changes in the IR spectrum, especially in the 3700–3500 cm^−1^ and 800–770 cm^−1^ spectral ranges (Fig. 3b and c). In the former spectral region, two well-defined bands at 3643 and 3556 cm^−1^ appear upon sending increasing H_2_O doses. The presence of those peculiar narrow bands in the O–H stretching region is ascribable to the interaction of H_2_O with specific acid sites.^37^ In the 800–770 cm^−1^ region, two initial bands at 790 and 779 cm^−1^ are observed in the evacuated sample (see black spectrum in Fig. 3c). After sending 0.3 mbar of H_2_O (corresponding to about 0.3–0.4% relative humidity, assuming a local temperature of around 313–318 K under the IR beam; vapour pressure of H_2_O at 313 K is 74 mbar, at 318 K is 96 mbar) the 779 cm^−1^ band shifts to 776 cm^−1^, before going back to the original position upon further increasing the H_2_O pressure. A new band at 771 cm^−1^ also appears at high H_2_O pressure. The 790 cm^−1^ signal decreases until 0.3 mbar of H_2_O, then it slowly shifts to 793 cm^−1^, changing again its intensity. The spectral modifications observed upon H_2_O contact in this region are probably due to changes occurring on the fluorinated rings. A more specific assignment of the bands present in those two spectral regions is provided by DFT calculations: in the high frequency region, the signals at 3643 and 3556 cm^−1^ can be disambiguated as the O–H stretching mode of water forming distinct H-bonds with F atoms on aromatic rings and O atoms from carboxylates, respectively. The assignment of bands in the low frequency region is less trivial, due to the coupling of different vibrations generating complex vibrational modes. In a simplified view, the observed signals can be attributed to the out-of-plane bending modes of carboxylate groups with respect to the aromatic rings. The coexistence of multiple features is explained by the presence of two crystallographically independent linkers (Fig. 1), as well as by the perturbation induced by adsorbates on the linkers themselves. It is worth noting that the main changes in the 800–770 cm^−1^ spectral region were observed with a H_2_O dose of 0.3 mbar (green curve in Fig. 3). This suggests that the exposure of the sample to a specific H_2_O pressure is responsible for a sudden change in the structure, hinting at an adsorption process similar to the one displayed by CO_2_ and possibly involving the same adsorption sites. This is supported by the very similar saturation loading of about 2.5 mmol g^−1^ for both water and CO_2_, corresponding to one adsorbate molecule per formula unit (see Fig. 2a and S15†).

*In situ* IR analysis using CO and N_2_ as molecular probes at liquid nitrogen temperature was performed to further study the presence of Lewis acidic adsorption sites in the evacuated MOF (Fig. S16†). Both the CO and N_2_ IR measurements highlight that, after H_2_O removal, F4_MIL-140A(Ce) features accessible Ce^IV^ sites available for interaction with Lewis bases, even as weak as N_2_. Detailed discussion of these results is available in Section S4 of the ESI.†

**Fig. 4:**
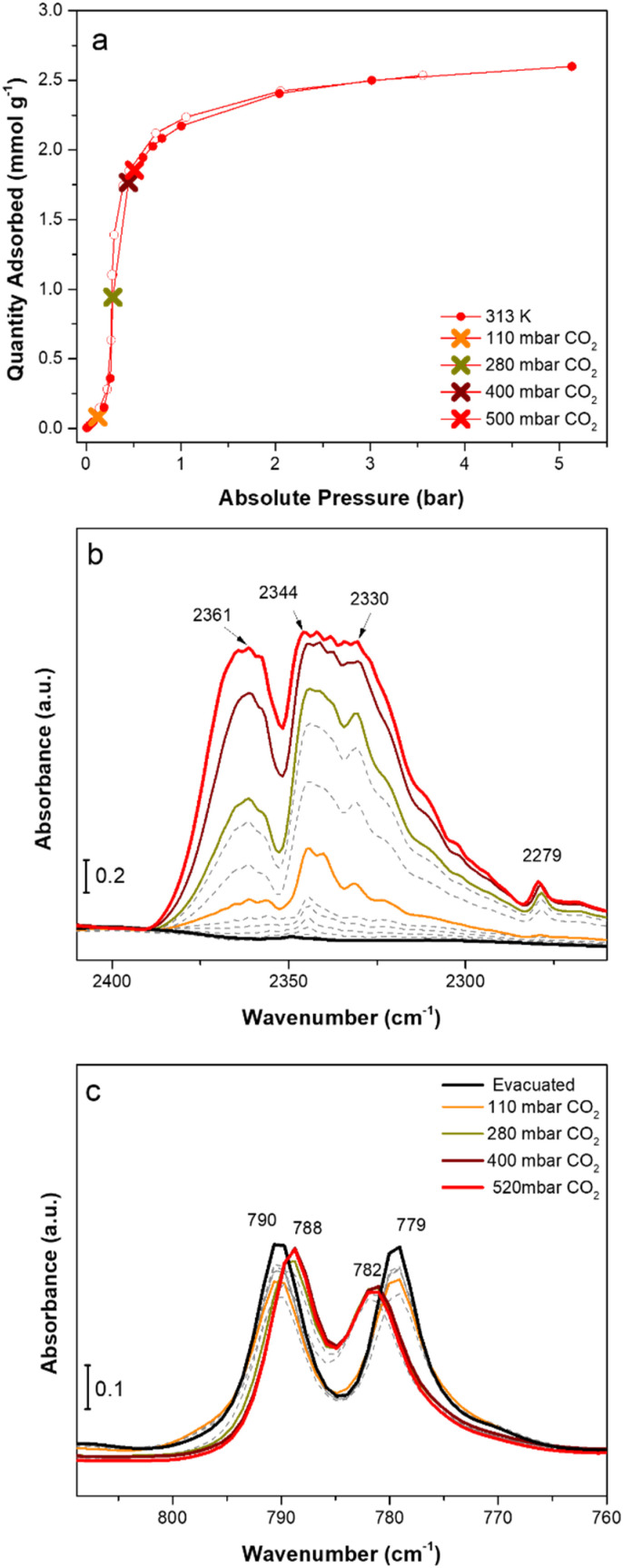
CO_2_ adsorption (filled circles) and desorption (empty circles) isotherms collected on F4_MIL-140A(Ce) at 313 K (40 °C) up to 5 bar (a). IR spectra of CO_2_ adsorption on evacuated F4_MIL-140A(Ce) reported in the 2410–2260 cm^−1^ (b) and 810–760 cm^−1^ (c) spectral ranges. The same incremental pressure doses of CO_2_ reported with coloured crosses in the volumetric isotherm of panel (a) were employed during the IR experiment. Dashed lines represent intermediate pressures.

Next, we investigated the vibrational behaviour of evacuated F4_MIL-140A(Ce) when exposed to an increasing CO_2_ pressure in the range between 110 and 550 mbar, at beam temperature (*i.e.*, around 313 K). The CO_2_ pressure range was accurately selected by considering the position of the step of the CO_2_ isotherm collected at 313 K (Fig. 4a). The incremental CO_2_ doses are responsible for significant changes in the CO_2_ asymmetric stretching region between 2400 and 2250 cm^−1^ (Fig. 4b) and in the 800–770 cm^−1^ spectral range (Fig. 4c). Upon sending CO_2_ doses below the isotherm step (*i.e.*, until around 110 mbar), a new, quite broad band at 2344 cm^−1^ appears, slightly shifted to lower frequencies compared to the antisymmetric CO_2_ stretching vibrational mode (at 2349 cm^−1^). At the same time, no significant changes are detected in the low frequency region, showing only a slight decrease in intensity of the 790 and 779 cm^−1^ bands. These spectral modifications suggest that, below the isotherm step, CO_2_ weakly interacts with the sorbent, likely because access to the open metal sites is prevented by the structural arrangement within channels. By increasing the CO_2_ pressure and approaching the isotherm step, a new band at 2361 cm^−1^, probably due to the interaction of CO_2_ with Ce^IV^, appears, while the 2344 cm^−1^ band increases giving rise to an intense band with apparent maxima at 2344 and 2330 cm^−1^. In general, the assignment of the above-mentioned bands is not straightforward because the rotovibrational profile of gaseous CO_2_, which falls in the same spectral region and prevails at high CO_2_ coverages, superimposes on those components. Concurrently, the bands at 790 and 779 cm^−1^ are shifted to 788 and 782 cm^−1^, respectively, suggesting that changes are occurring on the fluorinated aromatic rings. The CO_2_ doses corresponding to the final part of the adsorption step and the beginning of the isotherm plateau cause no further changes in the low frequency spectral range, while both the 2361 and the 2344 and 2330 cm^−1^ bands go out of scale. The predominance of the band at 2361 cm^−1^, possibly related to the direct interaction of CO_2_ with Ce^IV^ open metal sites, at CO_2_ pressures that correspond to the isotherm step could be a further proof of the occurrence of a phase-change adsorption process that enables the establishment of a strong adsorbate/surface interaction.

#### DFT optimisation of molecular adducts

IR experiments provided good evidence of the presence of open metal sites able to interact with various adsorbates. The energetics of interaction of F4_MIL-140A(Ce) with H_2_O, CO, N_2_ and CO_2_ were then investigated by means of periodic DFT calculations. Initially, the experimental structure of the MOF from ref. 28 was optimized in the absence of any adsorbed molecule, *i.e.* in its evacuated form. The adsorption of H_2_O, CO, N_2_ and CO_2_ was then simulated by manually placing the adsorbates at opportune positions within the MOF (*i.e.*, in the vicinity of the inorganic building unit) and by reoptimising the obtained structure. To fully exploit the framework symmetry, only full coverage (*i.e.*, 1 : 1 adsorbate/Ce ratio) was studied. For H_2_O, CO and N_2_, the molecule was positioned in direct interaction with the Ce open metal site through the O atom, the C atom and one of the N atoms, respectively. In the case of CO_2_, two possible adsorption sites were considered: (i) as for the other adsorbates, with an O atom in direct interaction with Ce (referred as “Ce” site); and (ii) positioned in the pore and interacting with the surrounding perfluorobenzene rings, as recently proposed by Zhao and coworkers^30^ (labelled here as “channel” site). The graphical representation of these adducts is presented Section 5 of the ESI,† whereas the main structural parameters are listed in Table S3.† The adsorption energetics for these adducts were also computed (details in Section 5 of the ESI†), as reported in Table 1.

**Table 1:** Adsorption electronic energy (Δ*E*) enthalpy (Δ*H*) and Gibbs free energy (Δ*G*) for F4_MIL-140A(Ce) in interaction with H_2_O, CO_2_ (for the latter, at both the “Ce” and the “channel” sites), CO and N_2_. All values are corrected for the basis set superposition error and given per adsorbed molecule. Δ*H* and Δ*G* values are computed at a temperature of 298.15 K and a pressure of 1013 mbar

Model	Δ*E* (kJ mol^−1^)	Δ*H* (kJ mol^−1^)	Δ*G* (kJ mol^−1^)
+H_2_O	−86.0	−77.8	−35.8
+CO_2_ (Ce)	−55.2	−46.1	−7.5
+CO_2_ (channel)	−35.2	−26.1	10.5
+CO	−40.5	−36.1	1.0
+N_2_	−32.2	−28.0	6.8

The energetic description of the adsorption phenomena suggests a strong preference of F4_MIL-140A(Ce) for H_2_O over the other investigated adsorbates. Interestingly, in the case of CO_2_, distinct interaction energies are computed depending on the adsorption site. In particular, the “channel” site, analogous to what previously proposed by Zhao and coworkers as the preferred one for CO_2_ adsorption, shows a lower interaction energy compared to that of CO_2_ in direct interaction with the open metal site. Furthermore, the Δ*G* values estimated for the adsorption occurring at the two sites point out that the interaction is thermodynamically favoured only for the “Ce” site, whereas the “channel” site is not providing a stable adduct at ambient conditions. In fact, the computed Δ*G* value for CO_2_ adsorption at the “channel” site is the highest observed in Table 1, suggesting that the involvement of the Ce^IV^ site is key for a favourable interaction with the adsorbates. A close inspection of the optimised crystal structures reveals that both H_2_O and CO_2_ are stabilised by interactions with both the metal centre (a Lewis acid), through their negatively polarised oxygen atoms, and the fluorine atoms belonging to the organic linker (Lewis bases), through the H atoms and C atom, respectively (Fig. S18–S24†). Thus, the ability of H_2_O and CO_2_ to behave as both Lewis acids and bases appears to be crucial to maximise their affinity for the adsorbent surface. We also simulated the interaction of CO_2_ with a fully hydrogenated MIL-140A(Ce) (Fig. S25†), observing a significant reduction in binding energy to the “Ce” site down to −39.4 kJ mol^−1^. This value is comparable to the strength of the interaction computed in the case of CO with F4_MIL-140A(Ce). Notably, the Gibbs free energy becomes positive in MIL-140A(Ce) (5.8 kJ mol^−1^), suggesting that the interactions with the linker also play a key role in stabilising the MOF-CO_2_ adduct.

### Structural characterisation

#### Powder X-ray crystallography: long range structure

**Fig. 5:**
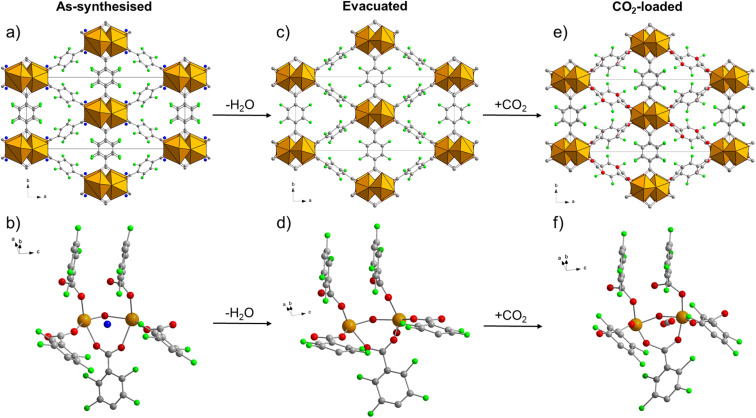
Comparison of the crystal structure viewed along the *c* axis and the local environment around the adsorption site of the as-synthesised (a and b, respectively), evacuated (c and d, respectively) and CO_2_-loaded (e and f, respectively) forms of F4_MIL-140A(Ce). Colour code: Ce, orange; F, green; C, grey; O, red; H_2_O, blue. H atoms not shown because their positions cannot be determined from PXRD data.

To investigate the structural response of F4_MIL-140A(Ce) upon evacuation and CO_2_ adsorption, we carried out an *in situ* synchrotron PXRD study. The as-synthesised MOF was first kept under dynamic vacuum, progressively heating up to 403 K. A phase transition occurs already below 373 K, corresponding with the removal of the adsorbed H_2_O molecules, and the new phase is stable when cooled down to 298 K (Fig. S26†). The structure retains the monoclinic *C*2/*c* space group during the phase transition. The main change in the unit cell is the increase of the *β* angle from 96.35(5)° in the as-synthesised MOF to 103.99(2)° in the evacuated one, which leads to a 2.7% decrease in volume from 2402(2) Å^3^ to 2336.0(5) Å^3^, respectively (Table S4†). The trend is in agreement with what suggested by DFT optimisation (Table S3†). Rietveld refinement of the PXRD pattern for the evacuated MOF was carried out starting from the DFT-optimised model, finding that H_2_O removal leads to rotation of the aromatic rings belonging to linker A (Table S5, Fig. 5a–d, S28–S29 and S44†). This structural rearrangement occurs in response to the loss of several interactions between the framework and the water molecules: H_2_O interacts with one Ce atom *via* its electron rich O atom and with at least three coordinated carboxylic O atoms and three F atoms (belonging to both linkers A and B) through hydrogen bonding (Fig. 1 and S44†).

The evacuated F4_MIL-140A(Ce) was then held at a constant temperature of 328 K and exposed to increasing CO_2_ pressures in the 0.055–1.485 bar range. As displayed in Fig. S30,† a phase transition occurs between 0.367 and 0.745 bar, corresponding to the pressure range where the CO_2_ adsorption isotherm collected at the same temperature displays the step (Fig. S31†). Upon reduction of the pressure from 1.485 down to 0.322 bar, the reverse transition takes place, regenerating the evacuated form of the MOF (Fig. S32†). An analogous, reversible phase transition is observed between 0.222 and 0.527 bar at 313 K, and between 0.833 and 1.490 bar at 343 K, in both cases in excellent agreement with the step position in the corresponding CO_2_ adsorption isotherms (Fig. S33–S36†). The PXRD pattern of the CO_2_-loaded MOF is consistent at the three temperatures, suggesting that the equilibrium structure is the same in the investigated temperature range (Fig. S37†). We also performed an isobaric experiment exposing the MOF to a constant pressure of 0.498 bar and decreasing the temperature from 343 to 313 K in 5 K steps (Fig. S38†). The MOF switches from the evacuated to the CO_2_-loaded form between 328 and 323 K, again in excellent agreement with what expected from the adsorption isotherms measured at different temperatures (Fig. S39†). Again, the unit cell parameters are only slightly affected by the phase transition, with the *β* angle decreasing from 103.99(2)° in the evacuated MOF to 97.68(1)° in the CO_2_-loaded one (Table S4†). This trend is also in agreement with what suggested by DFT optimisation (Table S3†). For the sake of Rietveld refinement of the CO_2_-loaded MOF, a structural model with CO_2_ adsorbed at the “Ce” site was adopted as the most probable guess, based on the DFT results previously discussed. Rietveld refinement was carried out on the PXRD pattern for the CO_2_-loaded MOF at 313 K, finding that the insertion of CO_2_ triggers rotation of linker A to a position similar to that observed for the as-synthesised MOF (Table S5,†Fig. 5e–f and S41). The CO_2_ molecule displays coordination-like interaction with two Ce atoms, *via* one of its electron-rich O atoms, besides additional interactions with three F atoms (belonging to both linkers A and B) and with two coordinated carboxylic O atoms, *via* the electron-poor C atom (Fig. S42†). Thus, CO_2_ appears to be adsorbed in a pocket where it is stabilised by a wealth of non-covalent interactions, akin to what seen for H_2_O in the as-synthesised MOF. The step observed in the CO_2_ adsorption isotherm most likely arises from the concerted rotation of perfluorinated aromatic rings upon the insertion of CO_2_ in such an adsorption site, once the activation barrier for ring rotation is overcome and the site is made accessible. Further support to this interpretation is provided herein by means of other characterisation techniques.

Comparing the three forms of F4_MIL-140A(Ce), it is evident that the as-synthesised and the CO_2_-loaded ones have common features, ascribable to the presence of the adsorbate, which are not shared by the evacuated form. By examining the torsional angles between the carboxylate groups and the aromatic rings for each linker in the different forms of the MOF (Table S5†), it can be seen that O2–C1–C2–C3 (linker A) and O5–C10–C9–C8 (linker B) torsional angles significantly change when adsorbates are present, by bringing the aromatic rings more out-of-plane respect to the carboxylates, as a result of interactions with the adsorbate. The third torsional angle, O4–C5–C6–C7 belonging to linker B, is instead only slightly affected, because of the lack of interactions with the adsorbates on that side of the linker (Fig. S43†).

#### XAS (Ce K-edge): local structure of the metal ion

**Fig. 6:**
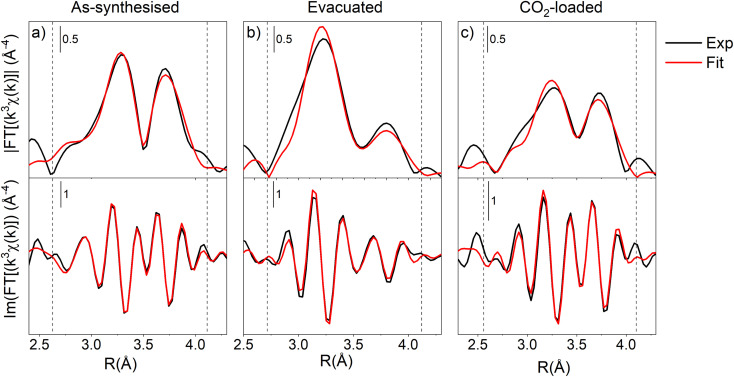
Comparison between experimental (black lines) and fitted values (red lines) of magnitude (top) and imaginary part (bottom) of phase-uncorrected Fourier transform of k^3^-weighted Ce K-edge EXAFS spectra for the as-synthesised (a), evacuated (b) and CO_2_-loaded (c) states. Dashed lines delimit the fitting range.

To complement the long-range structural analysis carried out by PXRD, Ce K-edge XAS spectra were collected on the as-synthesised, evacuated and CO_2_-loaded F4_MIL-140A(Ce) samples, respectively (Fig. S45†). This allowed us probing the local structure of Ce centres and gathering sensible information by fitting the extended X-ray absorption fine structure (EXAFS) signal as already done for several MOFs.^38–42^ The experiments were carried out at 328 K in dynamic conditions, *i.e.*, under a He flow for the evacuation step and under a controlled mixing of He and CO_2_ to follow the adsorption/desorption of CO_2_ within the porous system. The changes displayed by the EXAFS signal (Fig. S46a†) collected on F4_MIL-140A(Ce) during the adsorption process agree with what expected on the basis of the CO_2_ adsorption isotherm measured at 328 K (Fig. S9†). Moreover, the Fourier transform of the EXAFS signal (Fig. S46b†) shows evident changes in the 2.5–4 Å range, *i.e.*, the second shell of ligands from Ce centres. The signals observed in this region can be assigned, by looking at the crystallographic structure, to Ce–Ce, Ce–H_2_O and Ce–CO_2_ distances which are indeed being modified by adsorption/desorption processes. The scattering length of these three paths was parametrised in order to achieve a satisfactory data/parameter ratio allowing us to refine one Ce-O_w_ and two Ce–C_CO_2__ distances in addition to the Ce–Ce ones. By fitting the EXAFS signal using paths whose starting position is calculated from the crystallographic data coming from the Rietveld refinement, results in Fig. 6 and Table S6† are obtained.

Ce–Ce paths, whose refinement was compulsory because their distances fall very close to the Ce-adsorbates ones, gave values in excellent agreement with the ones observed by PXRD. The Ce-adsorbate distance for the as-synthesised material (Ce–O_w_) was refined at 3.368(3) Å, whereas the Ce–C_CO_2__ distances for the CO_2_-loaded one were fitted respectively to 4.06(3) Å and 4.44(4) Å. Such values are in agreement with those refined from PXRD data through Rietveld method (2.89 and 3.62 for Ce–O_w_; 4.04 and 4.44 for Ce–C_CO_2__) and from DFT optimisation (2.63 and 3.13 for Ce–O_w_; 3.93 and 4.20 for Ce–C_CO_2__), thus strengthening their reliability and consistency.

#### SSNMR: local structure of the adsorbate and the organic linker

The local structure of as-synthesised, evacuated, and CO_2_-loaded F4_MIL-140A(Ce) was further characterized by multinuclear SSNMR spectroscopy exploiting the ^19^F and ^13^C nuclei of the linkers. Moreover, for the as-synthesised and the CO_2_-loaded samples, ^1^H and ^13^C nuclei of water and CO_2_ were respectively investigated to unravel the state of the adsorbates in the MOF.

**Fig. 7:**
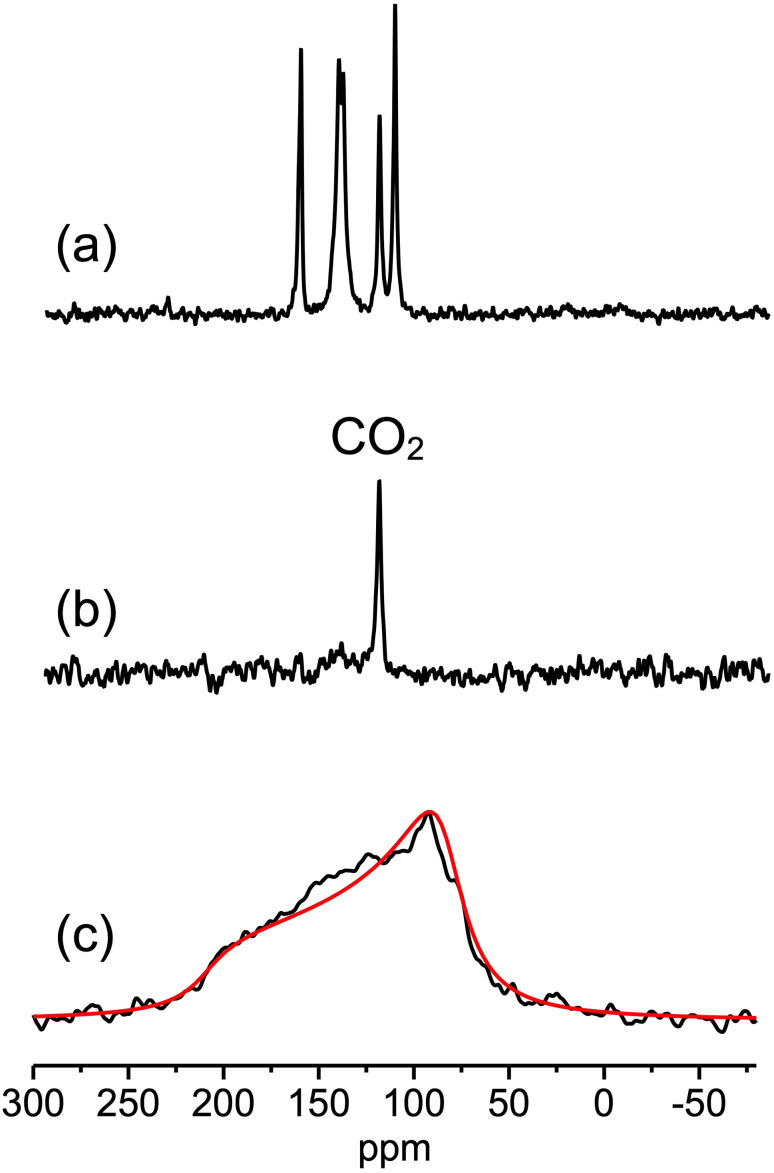
^13^C DE spectra of CO_2_-loaded F4_MIL-140A(Ce) recorded (a) under MAS with a recycle delay of 150 s, (b) under MAS with a recycle delay of 2 s, and (c) in static conditions with a recycle delay of 2 s.

The ^1^H direct excitation (DE) static spectrum of as-synthesised F4_MIL-140A(Ce) indicates the presence of both weakly physisorbed and structural water, which is completely removed after heating under vacuum, as shown by the absence of any signal in the spectrum of evacuated F4_MIL-140A(Ce) (Fig. S47†). The ^13^C DE spectrum of CO_2_-loaded F4_MIL-140A(Ce) (Fig. 7a), recorded under magic angle spinning (MAS) at a rate of 15 kHz and with a recycle delay between subsequent scans (150 s) sufficient to quantitatively observe the signals of MOF and CO_2_ carbons, indicates that almost one CO_2_ molecule (signal at 124.7 ppm) per Ce atom was adsorbed in the MOF when loaded at a CO_2_ pressure of 1 bar. When the ^13^C DE-MAS spectrum is acquired with a short recycle delay of 2 s, the sole signal of CO_2_ is selectively observed (Fig. 7b), since the ^13^C longitudinal relaxation time of CO_2_ carbons is much shorter than those of the linker carbons, in agreement with ref. 43. The ^13^C DE static spectrum recorded with the short recycle delay shows a powder pattern with a line shape arising from the anisotropic ^13^C chemical shift (CS) interaction averaged by CO_2_ dynamics (Fig. 7c), as found in other MOFs.^44^ The observed residual chemical shift anisotropy (CSA) indicates that CO_2_ reorientation in F4_MIL-140A(Ce) is not isotropic, the CO_2_ mobility being restricted by the interactions with the MOF linkers and Ce atoms and/or by steric hindrance in the pores. Generally, the CS interaction can be described with a second rank tensor with three principal components *δ*_11_, *δ*_22_, and *δ*_33_, with *δ*_11_ ≥ *δ*_22_ ≥ *δ*_33_ (“Mehring notation”). Three parameters are sufficient to describe the tensor: following the “Maryland notation”, they are the isotropic chemical shift [*δ*_iso_ = (*δ*_11_ + *δ*_22_ + *δ*_33_)/3], the span (*Ω* = *δ*_11_ − *δ*_33_), and the skew [*κ* = 3(*δ*_22_ − *δ*_iso_)/*Ω*].^45^ The span measures the breadth of the observed powder pattern and is used to indicate the extent of the CSA, while the skew, ranging from −1 to 1, gives an indication of the symmetry of the CS tensor, the two limit values of *κ* corresponding to an axial symmetry. For CO_2_ in the solid state, an axial CS tensor is reported in the literature, with *Ω* = 315−335 ppm and *κ* = 1.^46,47^ The signal observed for CO_2_ adsorbed on F4_MIL-140A(Ce) is instead characterised by *Ω* ≃ 127 ppm and *κ* = −0.98. The span reduction with respect to fully immobilised CO_2_ indicates that a motion with a rate higher than the spectral broadness in frequency units occurs for CO_2_ in F4_MIL-140A(Ce) at room temperature. Wobbling on an individual adsorption site and hopping among symmetry equivalent adsorption sites have been found for CO_2_ in other MOFs by ^13^C SSNMR spectroscopy.^48–53^ In our case, the negative value of *κ* suggests that a wobbling or hopping motion occurs with an angle (*β*) between the *C*_∞_ symmetry axis of CO_2_ and the motion axis larger than the magic angle (54.74°),^53^ while the axial line shape indicates a 3-fold or higher symmetry for the motion, which should occur in the fast regime. A satisfactory simulation of the spectral line shape (Fig. 7c) could indeed be obtained with a wobbling in a cone motion among 3 (or more) equivalent sites in the fast regime with *β* = 75°. It must be pointed out that this motion could be associated either to a reorientation of CO_2_ about a local axis or to the hopping of CO_2_ molecules among equivalent adsorption sites, also in combination with translation. The occurrence of both wobbling and hopping motions cannot be excluded. Further variable temperature ^13^C SSNMR static experiments on F4_MIL-140A(Ce) loaded with ^13^C isotopically enriched CO_2_ and line shape analyses are necessary to characterise CO_2_ dynamics in more detail.

^19^F DE and ^19^F–^13^C cross polarization (CP) experiments were also recorded under MAS on all samples to highlight possible effects of H_2_O or CO_2_ adsorption on linkers. The spectra, showing signals from the linker fluorine and carbon atoms, respectively, are reported in Fig. S48† and 8.

**Fig. 8:**
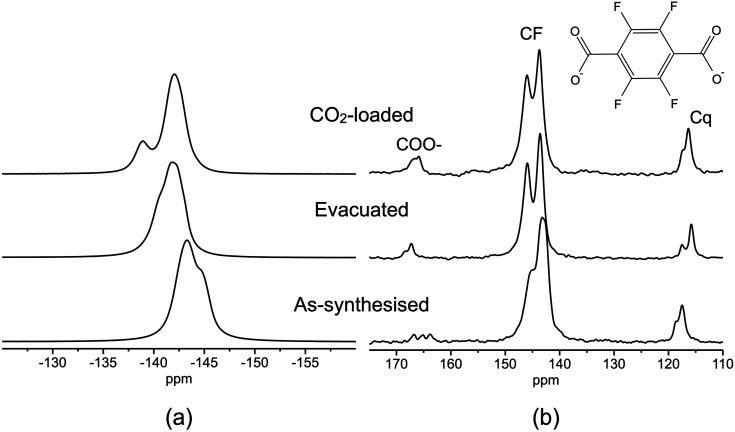
(a) ^19^F DE-MAS spectra and (b) ^19^F–^13^C CP-MAS spectra (ct = 1 ms) recorded on (from bottom to top) as-synthesised, evacuated, and CO_2_-loaded F4_MIL-140A(Ce).

^19^F DE-MAS spectra show broad spinning sideband patterns (Fig. S48†) due to the large chemical shift anisotropy typical of ^19^F nuclei.^54^ The ^19^F isotropic signals (Fig. 8a) lie in the spectral region between −150 and −135 ppm. A spectral deconvolution of the whole spinning sideband profiles showed that inequivalent fluorine atoms are present in all samples: two in the as-synthesised MOF, with isotropic chemical shifts of −143.1 and −144.8 ppm and intensities in a 3 : 1 ratio; three in the evacuated MOF, with chemical shifts of −141.0, −142.0, and −143.0 ppm and intensities in a 1 : 2 : 1 proportion; two in the CO_2_-loaded MOF with isotropic chemical shifts of −138.9 and −142.1 ppm and 1 : 3 relative intensities. In the ^19^F–^13^C CP-MAS spectra (Fig. 8b), the signals of fluorinated (CF), quaternary (Cq), and carboxylic (COO^−^) carbons of the linkers can be recognised in three distinct spectral regions. For each kind of carbon, several peaks are observed, as better appreciated after spectral deconvolution (Fig. S49†). The observed ^19^F and ^13^C multiple signals are ascribable to the two crystallographically inequivalent linkers in the structure of F4_MIL140 (Ce), namely, linker A and linker B. Analogous results were reported for MIL-140A(Zr).^55^ To distinguish signals arising from linkers A and B, the correlations occurring in 2D ^19^F–^13^C HETCOR spectra (Fig. S50–S52†) between signals of ^13^C and ^19^F nuclei in spatial proximity were also used. Therefore, signal assignment was possible combining the analysis of 1D ^19^F and ^19^F–^13^C CP-MAS spectra (recorded at different contact time values) with that of 2D ^19^F–^13^C HETCOR spectra. The results, shown in Table 2 for as-synthesised, evacuated, and CO_2_-loaded F4_MIL-140A(Ce), clearly indicate the presence of two different linkers with symmetry compatible with that found by the PXRD structural analysis (see Section 8 in the ESI† for a detailed description of signal assignment).

**Table 2:** Assignment of signals in 1D ^19^F DE-MAS and ^19^F–^13^C CP-MAS spectra (Fig. 8) and in 2D ^19^F–^13^C HETCOR spectra (Fig. S50–S52) of as-synthesised, evacuated, and CO_2_-loaded F4_MIL-140A(Ce)

Linker	Position	Atom	As-synthesised		Evacuated		CO_2_-loaded	
			*δ*(^13^C) (ppm)	*δ*(^19^F) (ppm)	*δ*(^13^C) (ppm)	*δ*(^19^F) (ppm)	*δ*(^13^C) (ppm)	*δ*(^19^F) (ppm)
A	CF	C4	145.3	−143.1	146.0	−142.0	143.6	−142.1
		C3	145.3	−143.1	146.0	−142.0	145.7	−142.1
	Cq	C2	117.6		115.8		116.3	
	COO^−^	C1	163.7		168.4		165.8	
B	CF	C8	143.5	−143.1	143.6	−141.0	145.7	−142.1
		C7	142.6	−144.8	143.6	−143.0	143.6	−138.9
	Cq	C9	117.6		117.4		117.2	
		C6	118.7		115.8		116.3	
	COO^−^	C10	166.9		167.3		167.2	
		C5	165.2		167.3		166.5	

Interestingly, shifts of ^13^C and ^19^F signals are observed among the as-synthesised, evacuated, and CO_2_-loaded MOF (Fig. 8 and Table 2), ascribable to the change of local chemical environment caused by adsorption of water or CO_2_. For instance, the shielding of ^19^F nuclei could be related to the interaction between the linker fluorine atoms and the adsorbate molecules,^56,57^ although the rotation of the phenyl rings out of the plane of carboxylates could also play a role. For as-synthesised F4_MIL-140A(Ce), ^13^C signals from COO^−^ and Cq carbons spatially close to water ^1^H nuclei are highlighted in the ^1^H–^13^C CP-MAS spectrum shown in Fig. S53,† since only for these carbons ^1^H–^13^C magnetisation transfer can effectively take place through dipolar coupling.

On the other hand, the signal of CO_2_ carbons was not observed in ^19^F–^13^C CP MAS spectra of CO_2_-loaded F4_MIL-140A(Ce) (Fig. 8b), even at contact time values as long as 10 ms. The distances between linker fluorines and CO_2_ in the Rietveld-refined structure of CO_2_-loaded F4_MIL-140A(Ce) (Fig. S42 and S43†) would allow sufficiently strong dipolar interactions to occur for an efficient magnetization transfer between ^19^F and ^13^C nuclei, as observed by other authors.^58^ The scarce CP efficiency found in our case should therefore be ascribed to a reduction of the dipolar interactions and/or of the residence time of CO_2_ in the adsorption site due to reorientational and translational motions.^59–61^

#### Energetics of the cooperative CO_2_ adsorption mechanism

**Fig. 9:**
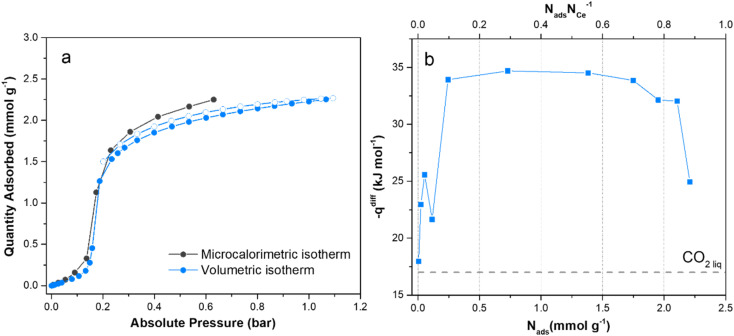
Comparison of volumetric isotherms obtained by performing CO_2_ adsorption at 303 K with the automatic volumetric instrument (blue) and with the volumetric line coupled with the microcalorimeter (black) (a). Differential molar adsorption heats curve related to the adsorption of CO_2_ at 303 K (b). The dotted horizontal line represents the standard molar heat of liquefaction of CO_2_ at 303 K: 17 kJ mol^−1^.

The foregoing discussion on the adsorption properties and structural response of F4_MIL-140A(Ce) to adsorption and desorption of H_2_O and CO_2_ suggests that a concerted mechanism takes place when CO_2_ is involved, due to the ease of rotation of the perfluorobenzene rings. The peculiar CO_2_ adsorption mechanism was further investigated by performing adsorption microcalorimetry at 303 K to directly measure the CO_2_ molar adsorption heat at different coverages (see the ESI† for further details). Fig. 9 reports the volumetric CO_2_ isotherms (a) and the differential molar adsorption heats (b) of F4_MIL-140A(Ce). The volumetric isotherm collected in the 0–0.6 bar pressure range with the microcalorimetric apparatus (dark grey curve in Fig. 9a) is perfectly in agreement with the isotherm obtained by the standard automatic volumetric instrument (light blue curve in Fig. 9a and S54† shows a comparison with the isotherms collected at 298 and 313 K). The differential molar adsorption heat curve (−*q*_diff_, Fig. 9b) unusually starts from low values, close to the molar heat of liquefaction of CO_2_ (17 kJ mol^−1^). The differential heat then increases until 25 kJ mol^−1^ and rapidly decreases to 22 kJ mol^−1^. After this intermediate step, −*q*_diff_ reaches a plateau at ∼34 kJ mol^−1^ corresponding to a coverage above 0.2 mmol g^−1^, *i.e.*, exactly where the step in the isotherm is taking place. These unusual values of differential heat clearly suggest that some endothermic modification of the adsorbent is occurring concurrently with the exothermic adsorption of CO_2_. Indeed, microcalorimetry provides the “average” heat of adsorption which could derive from different contributions generated by the interaction between the porous adsorbent and the adsorptive. If the endothermic event is particularly heat−demanding, the resulting net differential heat displays this peculiar trend.^62^ After reaching the value of 34 kJ mol^−1^, −*q*_diff_ remains constant until a coverage of 2 mmol g^−1^ (a value corresponding to the decrease in the slope of the adsorption isotherm immediately after the step). The relatively high value of −*q*_diff_ over a wide range of coverages suggests that CO_2_ strongly interacts with a homogeneous set of adsorption sites distributed on the internal surface of F4_MIL-140A(Ce).^38^ When all the available adsorption sites are covered by CO_2_, the differential heat curve steeply decreases, again approaching the heat of liquefaction of CO_2_. At the step, the differential heat curve is in agreement with the isosteric heat of adsorption plot, indirectly extracted from the CO_2_ isotherms by applying the Clausius–Clapeyron equation (Fig. S6†). However, the Clausius–Clapeyron model fails in identifying a region of low heat of adsorption before the step takes place.

DFT also provides further insights in the description of the adsorption phenomena: the initial q_diff_ is closely approached by the Δ*H* value computed for CO_2_ adsorbed at the “channel” site (see Table 1). At plateau, the *q*_diff_ value is instead comparable to that computed for CO_2_ at the “Ce” site, though slightly lower. The peculiar energy profile revealed by adsorption microcalorimetry could be explained by a scarce initial affinity of CO_2_ for the surface of the adsorbent. As a matter of fact, even though the “channel” site is not the most favourable adsorption site for CO_2_, the adsorptive must necessarily pass through the porous system to reach the most favourable “Ce” site. Furthermore, the displacement of CO_2_ implies the rearrangement of the material, *i.e.*, the concerted rotation of the perfluorobenzene rings, to enable access to the most energetic adsorption site. The occurrence of this endothermic phenomenon is clearly proved by the differential heat trend observed at the onset of the isotherm step.

**Fig. 10:**
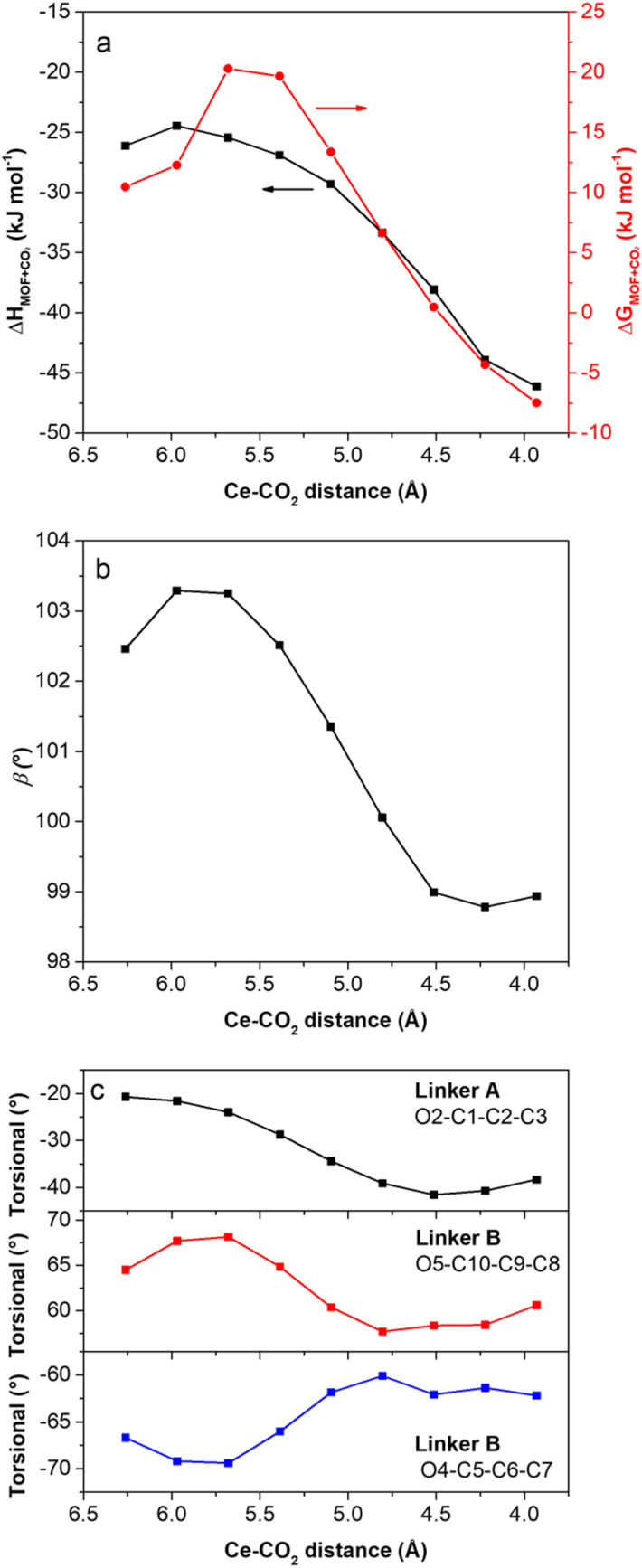
Trends of: (a) Δ*H* and Δ*G* for CO_2_ adsorption; (b) *β* angle of the monoclinic unit cell; and (c) perfluorobenzene ring-carboxylate torsional angles as a function of the Ce–CO_2_ distance. Δ*H* and Δ*G* values are computed at a temperature of 298.15 K and a pressure of 1013 mbar.

To give a quantitative description of the phenomenon, a relaxed potential energy surface scan was performed by DFT. In brief, CO_2_ initially modelled in the “channel” site, was stepwise approached to the “Ce” site. At each step, the whole structure was reoptimized, keeping the distance between the centre of mass of CO_2_ (*i.e.*, its C atom) and Ce constant. The trends of the energetic (Δ*H* and Δ*G*) and selected structural (*β* cell angle, O2–C1–C2–C3 torsional angle in linker A, O5–C10–C9–C8 and O4–C5–C6–C7 torsional angles in linker B) descriptors are reported as a function of the Ce–CO_2_ distance in Fig. 10.

The energy trend clearly shows the existence of an activation barrier (Δ*G* barrier of ∼10 kJ mol^−1^) that the CO_2_ molecule must overcome to reach the most favourable “Ce” site. As the barrier is overtaken, a drastic modification in the structural features of the MOF takes place, accompanied by a progressive stabilisation of the MOF-CO_2_ adduct. This rearrangement implies the distortion of the unit cell through a decrease of the *β* cell angle, as well as the rotation of the perfluorobenzene rings, as testified by the concomitant variation of their torsional angles compared to the carboxylates. The trend from DFT simulation is in full agreement with that observed by PXRD. The key role played by the Ce^IV^ open metal site in F4_MIL-140A(Ce) also explains the lack of step observed in the CO_2_ isotherm of its Zr-based analogue, recently reported by Zhao and co-workers.^30^ Indeed, Zr^IV^ is seven-coordinate in the MIL-140 topology, with no H_2_O coordinated in its as-synthesised form and, therefore, no available open metal sites for adsorbates to interact upon evacuation.

## Conclusions

Through a combination of experimental and computational methods, we have produced a coherent set of evidence supporting the existence of a cooperative mechanism of CO_2_ adsorption in F4_MIL-140A(Ce), which involves the concerted rotation of perfluorinated aromatic rings. This phenomenon opens the gate for CO_2_ to reach a highly favourable adsorption site, where it is stabilised by interactions with both coordinatively unsaturated Ce^IV^ sites and the organic linkers. The step-shaped isotherm of F4_MIL-140A(Ce) resulting from this phase-change behaviour allows to reach saturation in a narrow range of partial pressure compatible with real-life post-combustion capture requirements. While several MOFs are known to display either adsorption at coordinatively unsaturated metal sites or concerted ring rotation, to the best of our knowledge the combination of these two features is observed in F4_MIL-140A(Ce) for the first time. Furthermore, the phase transition has a low activation barrier and does not involve major restructuring of the framework, resulting in a complete reversibility in desorption and leading to a non-hysteretic behaviour. Based on the knowledge acquired with this work, we expect that the CO_2_ adsorption behaviour of F4_MIL-140A(Ce) can be finely tuned by both varying the fluorination degree of the organic linker and preparing mixed Ce/Zr analogues, paving the way towards the development of a class of adsorbents based on the same working principle. In view of the practical application of F4_MIL-140A(Ce) in post-combustion CO_2_ capture, its high affinity for water could be detrimental to the capture performance, as water might preferentially adsorb over CO_2_. We are currently investigating this aspect using dynamic breakthrough analysis, testing both dry and humid gas mixtures.

## Experimental procedures

### Gas adsorption volumetry

N_2_, Ar and CO_2_ isothermal physisorption measurements were performed on a Micromeritics ASAP 2020 analyser at 77 K, 87 K and 273 K, respectively. For CO_2_ adsorption the temperature was kept constant thanks to a home-made patented glass coating cell^63^ in which a coolant or heating fluid, connected to a thermostatic bath (JULABO F25), is recirculating. Prior to the measurement, the sample was heated overnight at 393 K under vacuum. Specific surface areas were determined by using the Brunauer–Emmett–Teller (BET) method, following the Rouquerol consistency criteria.^64^ Cumulative pore volume and pore size distribution were derived from adsorption isotherms by applying the NL-DFT method (see ESI,† Section 3, for further information).

High pressure CO_2_ adsorption–desorption isotherms up to 5 bar were measured with a Quantachrome iSorb High Pressure Gas Analyser at 298, 313, 328 and 343 K. About 200 mg of sample was used for the adsorption studies. The sample was degassed at 393 K under dynamic vacuum for 12 h prior to analysis and at 393 K for 1 h in between subsequent measurements.

### *In situ* IR spectroscopy

IR spectroscopy was performed on a Bruker Vertex 70 instrument equipped with a MCT (mercury cadmium tellurium) cryo-detector. All the spectra were recorded in the 4500–600 cm^−1^ spectral range with a resolution of 2 cm^−1^ and an average of 32 scans. Before the analysis, F4_MIL-140A(Ce), as self-supported pellet, was held in vacuum at 393 K for 12 h. Both CO and N_2_ interactions with the sample were studied by dosing a proper amount of gas (∼60 mbar) into the cell and cooling down to 77 K with liquid nitrogen. The spectra reported for both the experiments were collected in outgassing through subsequent expansions until complete desorption. H_2_O adsorption was carried out by sending incremental doses of H_2_O (from 0.1 to 20 mbar) on the evacuated F4_MIL-140A(Ce). The CO_2_ interaction with F4_MIL-140A(Ce) was studied by sending increasing CO_2_ doses on the sample after applying the same evacuation procedure previously described. The increasing doses of CO_2_ were chosen to be compatible with the equilibrium pressures involved in the adsorption/desorption isotherm at 313 K (40 °C) which is approximately the IR beam temperature.

### Periodic DFT simulations

Vibrational and energetic features of F4_MIL-140A(Ce), also in the presence of relevant adsorbates, were simulated at the DFT level of theory with the CRYSTAL17 code.^65,66^ In detail, the B3LYP hybrid GGA functional^67,68^ was exploited in conjunction with a double-ζ quality basis set (C and O from ref. 39; F from ref. 69; and Ce from ref. 70). A larger Ahlrichs TZV2p basis^39^ described atoms belonging to adsorbed molecules (H_2_O, CO_2_, CO and N_2_). Dispersive interactions were empirically included in the Hamiltonian according to the Becke-Johnson dumped version of the Grimme's D3 scheme.^71,72^ Such computational setup provided a satisfactory structural and electronic description of Ce^IV^-MOFs.^73^

### *In situ* PXRD

The *in situ* PXRD study here described took place at Beamline P02.1 (PETRA III, DESY, Hamburg).^74^ PXRD patterns were collected using 60.0 keV (0.207124 Å) radiation. The sample was held in a 0.5 mm internal diameter glass capillary, which was mounted on a custom-built spinner device that allows for rotating a gas-filled capillary mounted on a commercial Huber goniometer head by 360° forth and back. The temperature of the sample was controlled between 298 and 413 K with an Oxford Hot-Air Blower. The beamline was equipped with a PerkinElmer XRD1621 CN3 – EHS detector (200 × 200 μm^2^ pixel size, 2048 × 2048 pixel area). The detector was positioned at 1000 mm from the sample stage. Each PXRD pattern was collected in the 0.0071–16.3041 °2*θ* range, with a 0.0067 °2*θ* step size and a total acquisition time of 30 s. The resulting 2D images were azimuthally integrated to 1D diffraction patterns using the software Fit2D.^75^

### *In situ* XAS

Ce K-edge XAS data were collected at the BM23 (ref. 76) beamline of the European Synchrotron Radiation Facility (ESRF) in Grenoble, France. The storage ring was operating in the 32-bunch mode with a target current of 150 mA. The measurements were conducted in transmission mode using a Si(311) double-crystal monochromator. The intensity of the X-ray beam was measured by means of three ion chambers (30 cm, 1 kV) placed before the sample (I_0_, filling 0.28 bar Kr + 1.72 bar He), after the sample (I_1_, filling 1.35 bar Kr + 0.65 bar He) and after the CeO_2_ reference sample (I_2_, filling 1.35 bar Kr + 0.65 bar He). This experimental setup allowed us to reference the energy to the edge of the reference sample and thus calibrate the energy for each spectrum.

### SSNMR spectroscopy

SSNMR spectroscopy measurements were carried out on a Bruker Avance Neo spectrometer working at Larmor frequencies of 500.13, 470.59, and 125.77 MHz for ^1^H, ^19^F, and ^13^C nuclei, respectively, equipped with a double-resonance cross polarization – magic angle spinning (CP-MAS) probe head accommodating rotors with an external diameter of 4 mm. MAS experiments were performed at a spinning frequency of 15 kHz. All spectra were acquired at room temperature using air as spinning gas. The chemical shift for all nuclei was referenced to the ^13^C signal of adamantane at 38.46 ppm. Further details on experimental settings for the acquisition of specific spectra are given in Section 8 of ESI.†

Evacuated and CO_2_-loaded F4_MIL140A(Ce) samples for SSNMR measurements were prepared using a home-made cell provided with a mechanical lever operated from outside enabling the capping of the rotor without disturbing the cell atmosphere. For preparing the evacuated sample, the unsealed rotor containing the as-synthesised packed sample was heated overnight under vacuum (0.1 mbar) at the temperature of 423 K within the cell and then sealed under N_2_ atmosphere. For CO_2_ loading, the home-built cell containing the evacuated sample packed into the rotor was loaded with CO_2_ at 1 bar pressure and the rotor was sealed under the gas atmosphere.

### Adsorption microcalorimetry

The molar differential heat of CO_2_ adsorption was evaluated on F4_MIL-140A(Ce) by means of a heat flow microcalorimeter (Calvet C80 by Setaram) connected to a high-vacuum (≈10^−4^ mbar) glass line equipped with a Varian Ceramicell 0–100 mbar gauge and a Leybold Ceramicell 0–1000 mbar gauge. The sample was held in vacuum at 393 K for 12 h before being placed into the calorimeter under isothermal conditions. The measurement was performed at 303 K by following a well-established step-by-step procedure described elsewhere.^77^

## Author contributions

M. C., V. C. and M. T. carried out the gas sorption experiments and data analysis. M. C., C. A. and V. C. carried out the IR and adsorption microcalorimetry experiments and data analysis. C. A., D. M. V., K. A. L. and M. T. carried out the EXAFS experiments. C. A. carried out EXAFS data analysis. M. S. performed the DFT calculations and data analysis. F. C., A. K. and M. T. carried out the PXRD experiments. M. T. carried out PXRD data analysis. L. C., F. M., A. G. and M. G. carried out the SSNMR experiments and data analysis. M. C., C. A., M. S., L. C., F. M., V. C. and M. T. drafted the manuscript. V. C. and M. T. coordinated the work. M. T. conceived the study.

## Conflicts of interest

There are no conflicts of interest to declare.

## Supplementary Material

TA-011-D2TA09746J-s001

TA-011-D2TA09746J-s002

TA-011-D2TA09746J-s003
