# Supplementary material for: Cooperative CO2 adsorption mechanism in a perfluorinated CeIV-based metal organic framework
Source: J Mater Chem A Mater. 2023 Feb 14;11(11):5568–83. doi: 10.1039/d2ta09746j (PMC10012411; doi:10.1039/d2ta09746j)
Supplement: TA-011-D2TA09746J-s002 [file TA-011-D2TA09746J-s002.pdf]

Electronic Supplementary Material (ESI) for Journal of Materials Chemistry A.
This journal is © The Royal Society of Chemistry 2023

Cooperative CO_2 adsorption mechanism in a perfluorinated Ce^{IV}-based metal organic framework

Margherita Cavallo,[1] Cesare Atzori,[1,2] Matteo Signorile,[1] Ferdinando Costantino,[3] Diletta Morelli Venturi,[3] Athanasios Koutsianos,[4,‡] Kirill A. Lomachenko,[2] Lucia Calucci,[5,6] Francesca Martini,[6,7] Andrea Giovanelli,[7] Marco Geppi,[6,7] Valentina Crocellà,[1,*] Marco Taddei[6,7,8*]

[1] *Dipartimento di Chimica, Centro di riferimento NIS e INSTM, Università di Torino, Via G. Quarello 15, I-10135 and Via P. Giuria 7, I-10125 Torino, Italy. Email: valentina.crocella@unito.it*

[2] *European Synchrotron Radiation Facility, 71 Avenue des Martyrs, CS 40220, 38043 Grenoble Cedex 9, France*

[3] *Dipartimento di Chimica Biologia e Biotecnologia, Unità di Ricerca INSTM, Università di Perugia, Via Elce di Sotto 8, 06123 Perugia, Italy*

[4] *Centre for Research & Technology Hellas/Chemical Process and Energy Resources Institute, 6th km. Charilaou-Thermis, 57001, Greece*

[5] *Istituto di Chimica dei Composti Organo Metallici, Unità di Ricerca INSTM, Consiglio Nazionale delle Ricerche, Via Giuseppe Moruzzi 1, 56124 Pisa, Italy*

[6] *Centro per l'Integrazione della Strumentazione Scientifica dell'Università di Pisa (CISUP), 56126 Pisa, Italy*

[7] *Dipartimento di Chimica e Chimica Industriale, Unità di Ricerca INSTM, Università di Pisa, Via Giuseppe Moruzzi 13, 56124 Pisa, Italy. Email: marco.taddei@unipi.it*

[8] *Energy Safety Research Institute, Swansea University, Fabian Way, Swansea, SA1 8EN UK*

‡ *Present address: Anorganische Materialchemie, Fakultät für Chemie & Chemische Biologie, Technische Universität Dortmund, Otto-Hahn-Straße 6, 44227 Dortmund, Germany*

Electronic Supplementary Information

Contents

1. Materials 3
2. Synthesis of F4_MIL-140A(Ce) 3
3. Gas sorption analysis 4
4. *In situ* IR spectroscopy 14
5. Periodic density functional theory (DFT) simulations 16
6. *In situ* powder X-ray diffraction (PXRD) 24
7. *In situ* X-ray absorption spectroscopy (XAS) 36
8. Solid state nuclear magnetic resonance (SSNMR) 39
9. Adsorption microcalorimetry 46
10. Supplemental references 48
11. Appendix: Coordinates of DFT-computed crystal structures 51

1. Materials

Cerium(IV) ammonium nitrate (16774-21-3, 99%, $Ce(NH_4)_2(NO_3)_6$, Sigma-Merck), nitric acid (7697-37-2, 68%, HNO_3, Sigma-Merck), tetrafluoroterephthalic acid (652-36-8, 97%, $C_8H_2F_4O_4$, Fluorochem). All reagents were used as received, with no further purification.

2. Synthesis of F4_MIL-140A(Ce)

F4_MIL-140A(Ce) was synthesised following a literature procedure:[1] tetrafluoroterephthalic acid (179 mg, 0.75 mmol) was dissolved in water (10.5 mL) and 16 mol L^{-1} HNO_3 (1.5 mL, 24 mmol) in a 20 mL scintillation vial, which was kept at 60 °C under stirring in an aluminium heating block. After 10 minutes, a solution of Cerium(IV) ammonium nitrate (411 mg, 0.75 mmol) in water (3 mL) was added in the vial and the mixture was left to react for 1 h. At the end of the reaction, the yellow solid was centrifuged, washed twice with water (15 mL each time) and finally washed with acetone (15 mL). The solid was dried in an oven at 80 °C. Yield: 177 mg (60%). Larger amounts of the MOF were prepared by parallel syntheses carried out on the same scale as the one described here.

3. Gas sorption analysis

N_2, Ar and CO_2 isothermal physisorption measurements were performed on a Micromeritics ASAP 2020 analyser at 77 K, 87 K and 273 K, respectively. (Figure S7) For CO_2 adsorption the temperature was kept constant thanks to a home-made patented glass coating cell[2] in which a coolant or heating fluid, connected to a thermostatic bath (JULABO F25), is recirculating. Prior to the measurement, the sample was heated overnight at 393 K under vacuum. Specific surface areas were determined by using the Brunauer−Emmett−Teller (BET) method, following the Rouquerol consistency criteria (Figure S8-S10).[3] Cumulative pore volume and pore size distribution were derived from adsorption isotherms by applying the NL-DFT method with the Microactive software provided by Micromeritics, using: (i) a cylindrical pore geometry and considering the model "Argon on Oxides at 87 K" for Ar adsorption at 87 K; (ii) a cylindrical pore geometry and considering the model "N_2 Cylindrical Pores Oxides Surfaces" for N_2 adsorption at 77 K; (iii) a slit pore geometry and considering the model "CO_2 – GCMC CO_2 Carbon " for CO_2 adsorption at 273 K (Figure S11-S14).

High pressure CO_2 adsorption-desorption isotherms up to 5 bar were measured with a Quantachrome iSorb High Pressure Gas Analyser at 298, 313, 328 and 343 K (Figure 2a). About 200 mg of sample was used for the adsorption studies. The sample was degassed at 393 K under dynamic vacuum for 12 h prior to analysis and at 393 K for 1 h in between subsequent measurements. The adsorption branches were fitted using the following version of the Dual Site Langmuir-Freundlich equation, implemented in *OriginLab*:

$$q = \frac{q_1 (K_1 P)^{n_1}}{1+(K_1 P)^{n_1}} + \frac{q_2 (K_2 P)^{n_2}}{1+(K_2 P)^{n_2}}$$

Where q is the gas loading at pressure P, q_1 and q_2 are the saturation loadings for each site, K_1 and K_2 are the affinity constants for each site, n_1 and n_2 are the indices of heterogeneity for each site. We note that the physical meaning of the model might not be representative of the actual CO_2 adsorption phenomenon occurring in F4_MIL-140A(Ce), but it provides an excellent fit of the experimental data nonetheless (see Figures S1-S4 and Table S1). Using these fitted isotherms, the isosteric heat of adsorption (Qst) was extracted using the linear version of the Clausius-Clapeyron equation in the loading range 0.1-2.2 mmol g^{-1} (Figure S6).

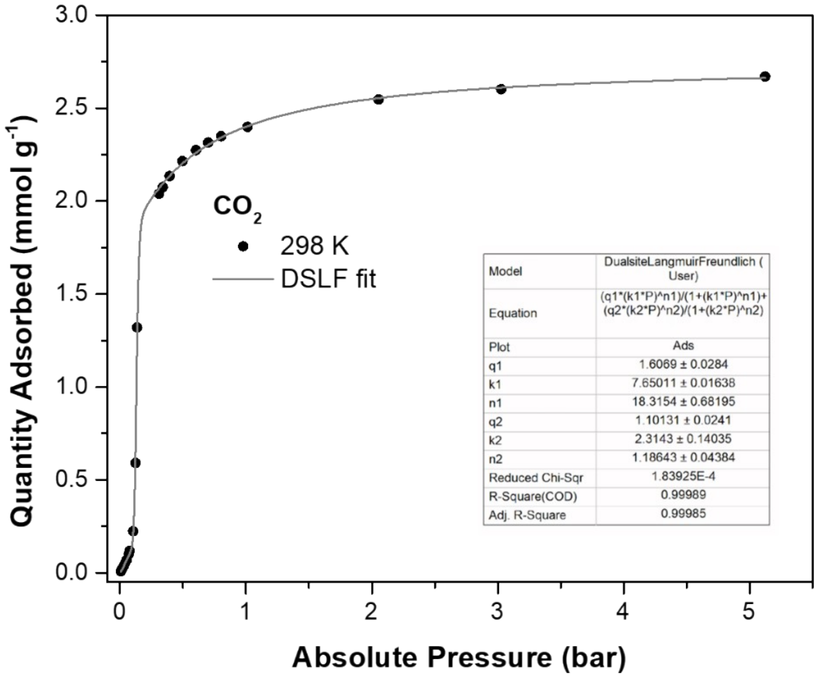

Figure S1. Dual site Langmuir Freundlich (DSLF) fit (grey line) of the CO_2 adsorption isotherm collected at 298 K (black circles) up to 5 bar.

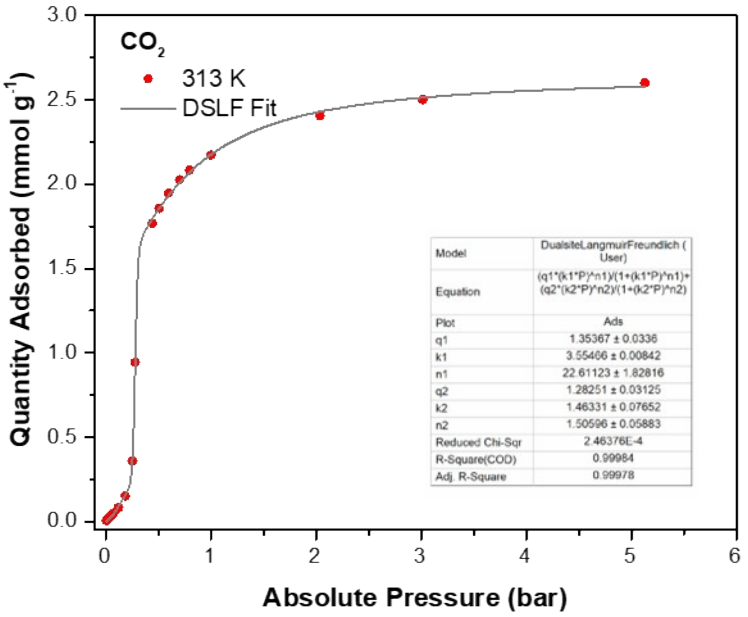

Figure S2. Dual site Langmuir Freundlich (DSLF) fit (grey line) of the CO_2 adsorption isotherm collected at 313 K (red circles) up to 5 bar.

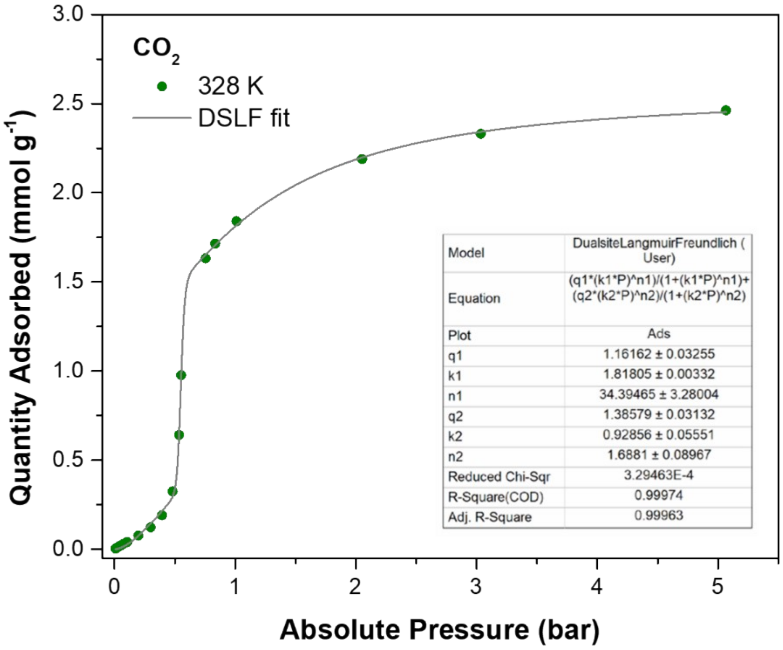

Figure S3. Dual site Langmuir Freundlich (DSLF) fit (grey line) of the CO_2 adsorption isotherm collected at 328 K (green circles) up to 5 bar.

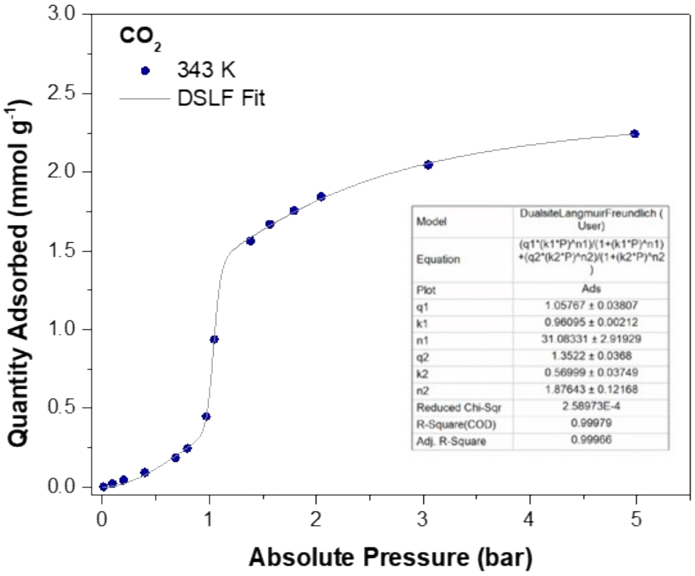

Figure S4. Dual site Langmuir Freundlich (DSLF) fit (grey line) of the CO_2 adsorption isotherm collected at 343 K (blue circles) up to 5 bar.

Table S1. Dual site Langmuir Freundlich fitting parameters of the CO_2 adsorption isotherms collected at 298 K, 313 K, 328 K and 343 K up to 5 bar.

Temperature (K)	q_1	k_1	n_1	q_2	k_2	n_2	R^2
**298**	1.61 ± 0.03	7.65 ± 0.02	18.3 ± 0.7	1.10 ± 0.02	2.3 ± 0.1	1.19 ± 0.04	0.99985
**313**	1.35 ± 0.03	3.555 ± 0.008	23 ± 2	1.28 ± 0.03	1.46 ± 0.08	1.51 ± 0.06	0.99978
**328**	1.16 ± 0.03	1.818 ± 0.003	34 ± 3	1.39 ± 0.03	0.93 ± 0.06	1.69 ± 0.09	0.99963
**343**	1.06 ± 0.04	0.961 ± 0.002	31 ± 3	1.35 ± 0.04	0.57 ± 0.04	1.9 ± 0.1	0.99966

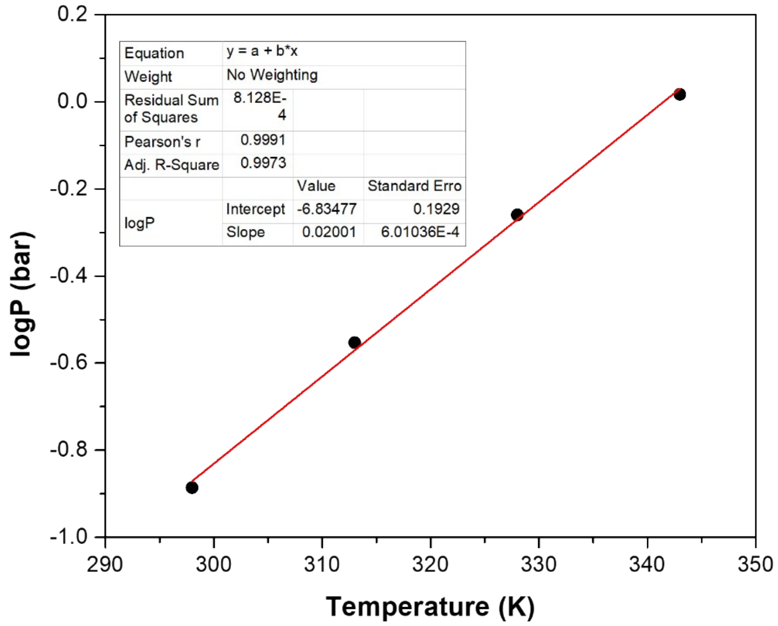

Figure S5. Plot of logP at which the first derivative of the adsorption isotherm reaches the maximum value as a function of the temperature (black circles) and linear fitting (red line). The very good correlation (R^2 = 0.9973) suggests that the threshold pressure for phase change in the 298-343 K temperature range may be straightforwardly predicted.

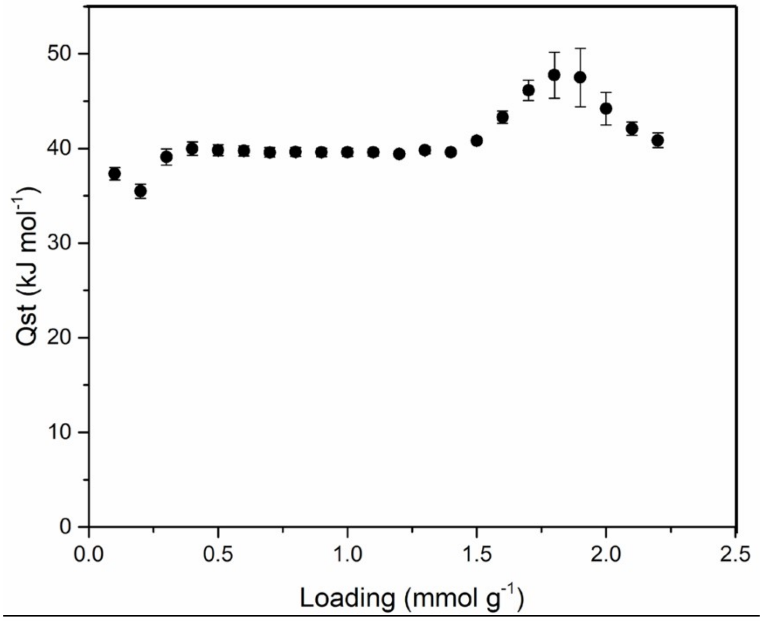

Figure S6. Plot of isosteric heat of CO_2 adsorption (Qst) versus loading obtained from the isotherms collected at 298 K, 313 K, 328 K and 343 K up to 5 bar. The "bump" between 1.5 and 2.0 mmol g^{-1} loading is probably an artefact originating from the uncertainty in the fitting in that region, where the isotherms display a change of gradient near saturation and few or no experimental data are available (See Figures S1-4).

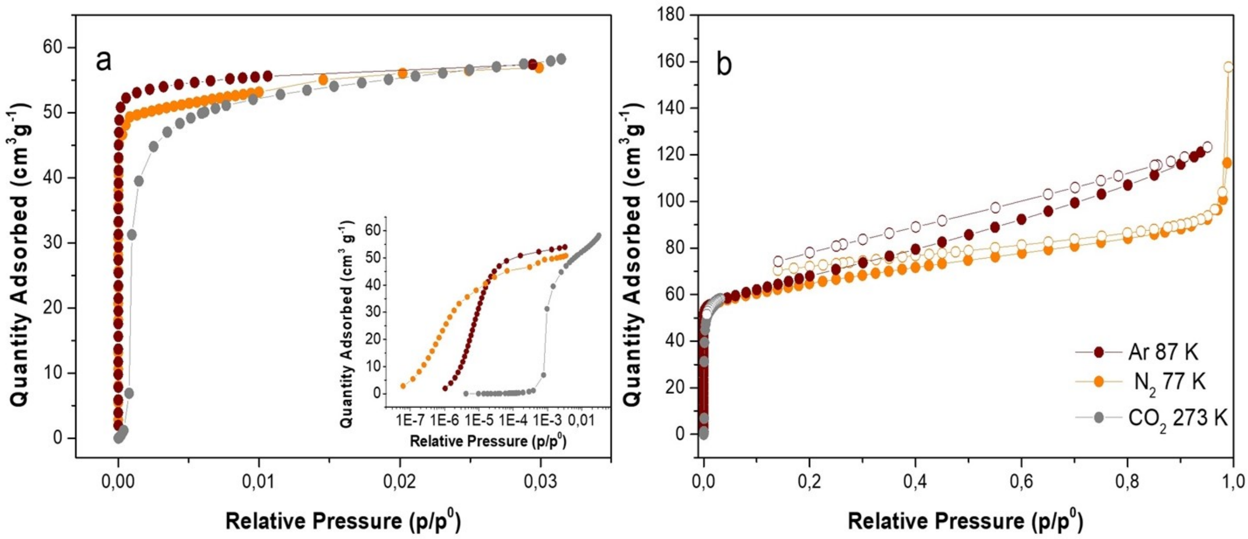

Figure S7. Volumetric adsorption isotherms in the 0 < p/p0 < 0.03 pressure range (semi-logarithmic isotherms in the same pressure range displayed in the inset (a) and volumetric adsorption/desorption isotherms in the whole pressure range of: Ar at 87 K (dark red), N_2 at 77 K (orange) and CO_2 at 273 K (grey) on F4_MIL-140A(Ce) (b).

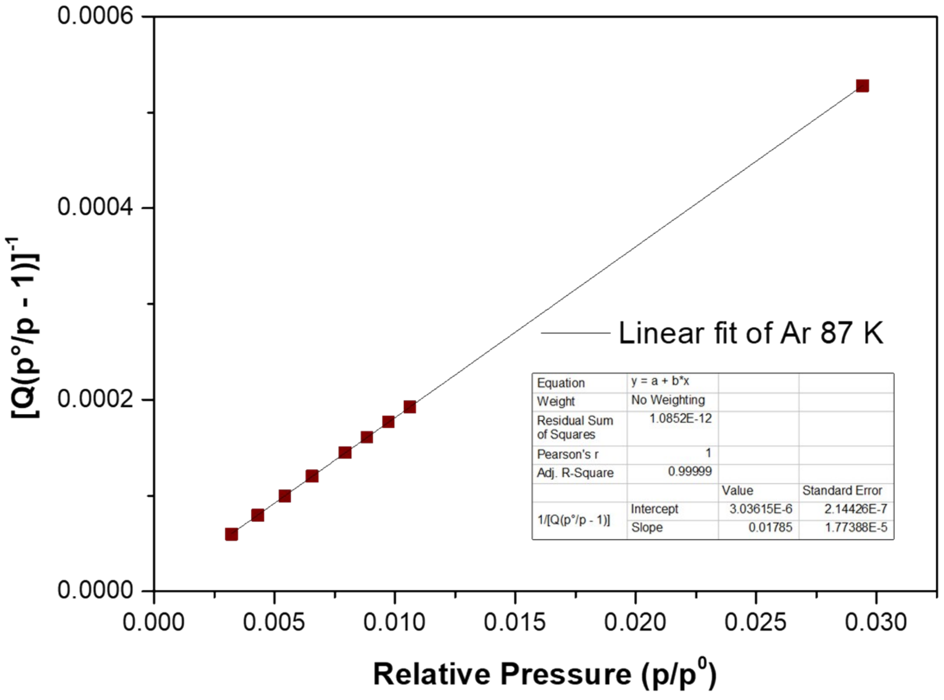

Figure S8. Linear BET fit in the $0.0032<p/p^0<0.036$ pressure range for Ar at 87 K.

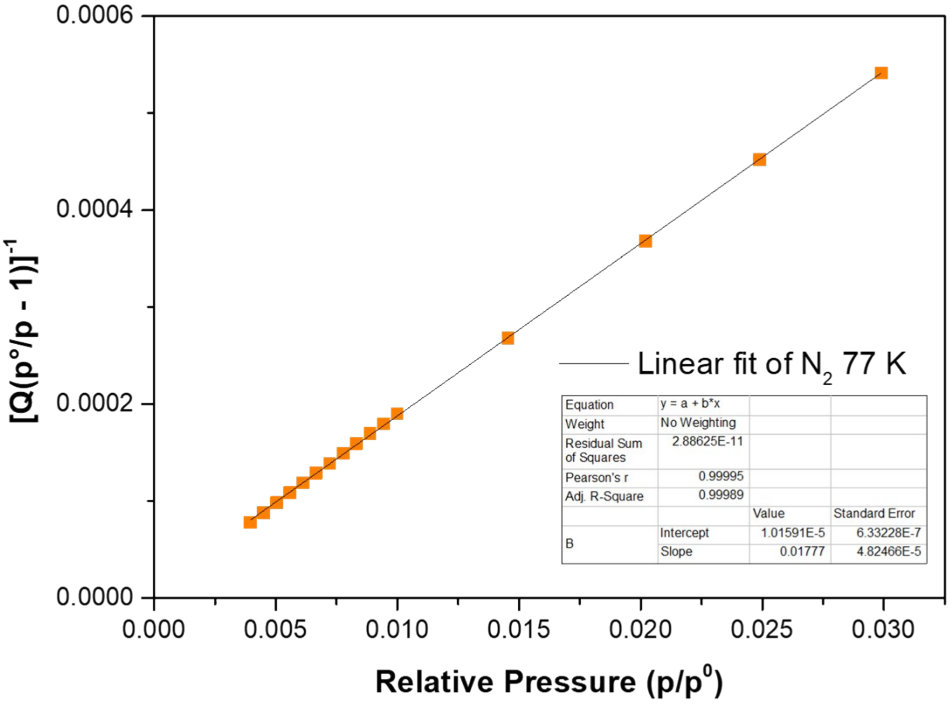

Figure S9. Linear BET fit in the $0.0032<p/p^0<0.036$ pressure range for N_2 at 77 K.

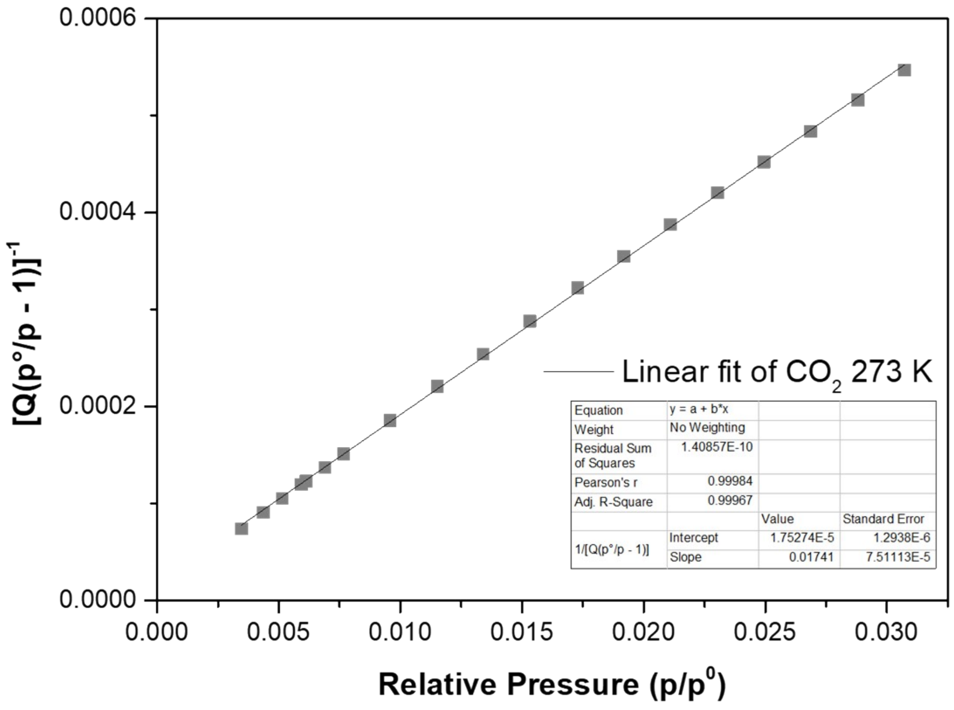

Figure S10. Linear BET fit in the 0.0028<p/p0<0.031 pressure range for CO_2 at 273 K.

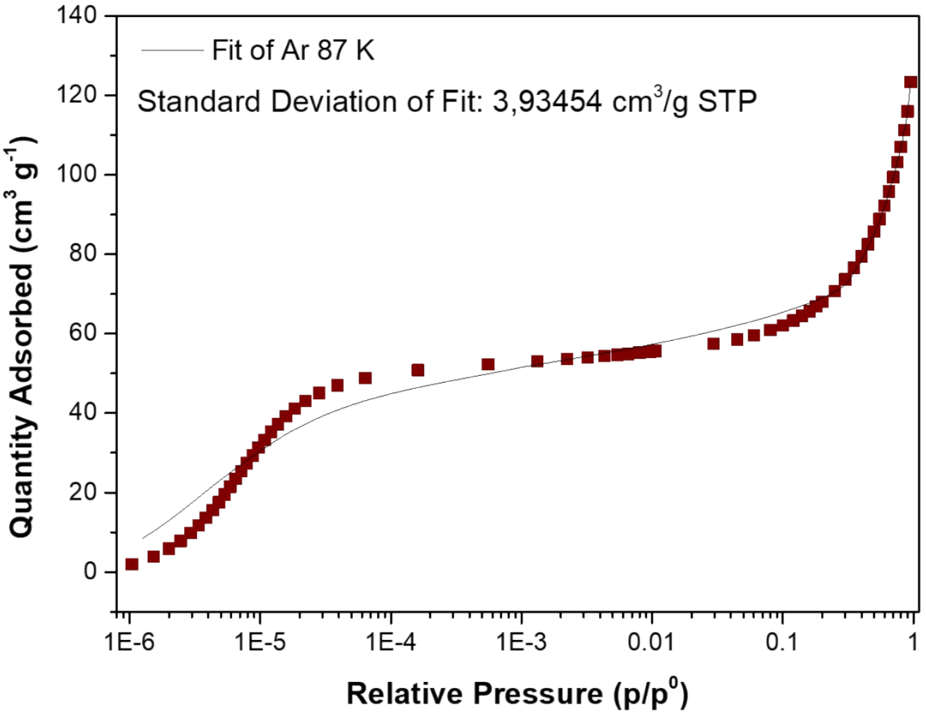

Figure S11. NL-DFT Pore Size Log Goodness of Fit Graph obtained by applying the NL-DFT analysis to the adsorption isotherm, employing a cylindrical pore geometry and considering the model “Argon on Oxides at 87 K” present in the Microactive software.

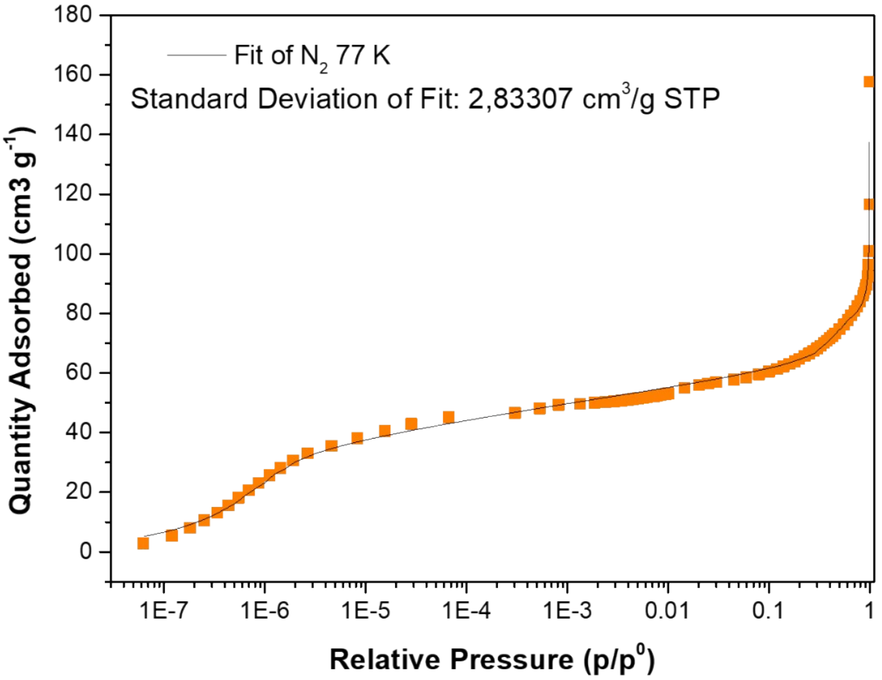

Figure S12. NL-DFT Pore Size Log Goodness of Fit Graph obtained by applying the NL-DFT analysis to the adsorption isotherm, employing a cylindrical pore geometry and considering the model “N_2 Cylindrical Pores Oxides Surfaces” present in the Microactive software.

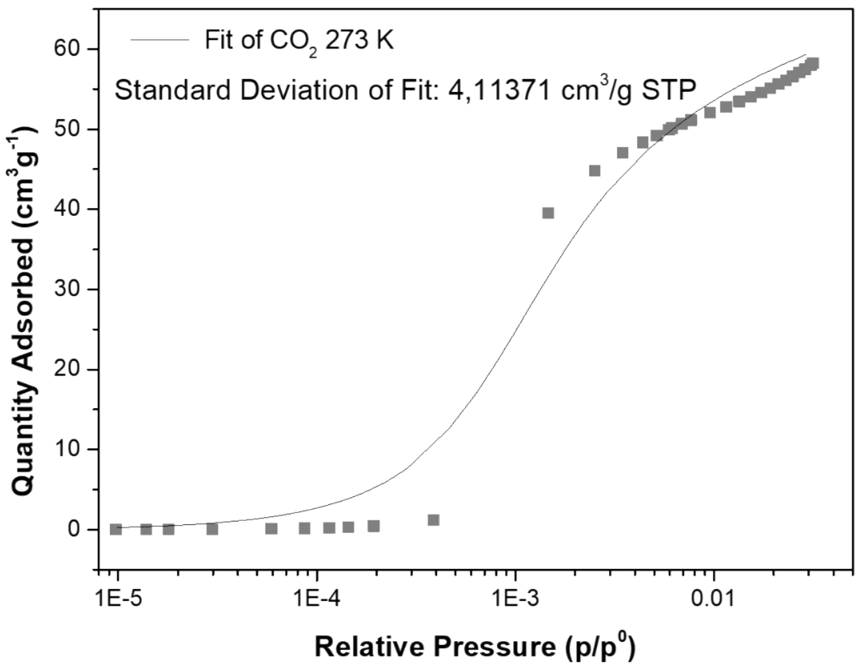

Figure S13. NL-DFT Pore Size Log Goodness of Fit Graph obtained by applying the NL-DFT analysis to the adsorption isotherm, employing a slit pore geometry and considering the model "CO_2 – GCMC CO_2 Carbon" present in the Microactive software.

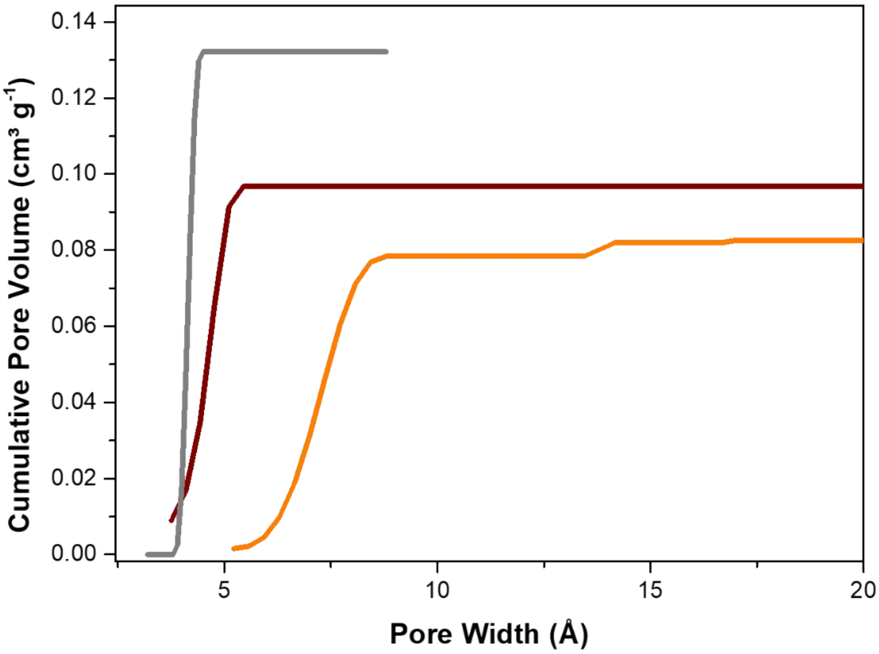

Figure S14. Cumulative pore volume graph derived from NL-DFT analysis of Ar at 87 K (dark red) N_2 at 77 K (orange) and CO_2 at 273 K (grey).

Table S2. Textural Properties of F4_MIL-140A(Ce) evaluated by means of three different molecular probes: Ar at 87 K, N_2 at 77 K and CO_2 at 273 K.

Probe	Temperature (K)	BET area (m^2 g^{-1})	Micropore volume (cm^3 g^{-1})	Micropore size (Å)
**Ar**	87	213.8 ± 0.3 [a]	0.096 [c]	4.8 [f]
**N_2**	77	244.8 ± 0.7 [a]	0.083 [d]	7.4 [g]
**CO_2**	273	262 ± 1 [b]	0.13 [e]	4.2 [h]

[a] BET Areas were determined in the 0.0032<p/p0<0.036 range, applying the Rouquerol consistency criteria.
[b] BET Area was determined in the 0.0028<p/p0<0.031 range, applying the Rouquerol consistency criteria.
[c] Calculated using the cumulative pore volume graph (Figure S8), obtained from NL-DFT analysis of the adsorption isotherm employing a cylindrical pore geometry and considering the model "NLDFT- Argon on Oxides at 87 Kelvin" present in the Microactive software.
[d] Calculated using the cumulative pore volume graph (Figure S8), obtained from NL-DFT analysis of the adsorption isotherm employing a cylindrical pore geometry and considering the model "N2 - Cylindrical Pores- Oxides Surfaces" present in the Microactive software.
[e] Calculated using the cumulative pore volume graph (Figure S8), obtained from NL-DFT analysis of the adsorption isotherm employing a slit pore geometry and considering the model "CO2 – GCMC Carbon" present in the Microactive software.
[f] Derived from the pore size distribution graph (Figure 2B) obtained from the NL-DFT analysis of the adsorption isotherm, employing a cylindrical pore geometry and considering the model "NLDFT- Argon on Oxides at 87 Kelvin" present in the Microactive software.
[g] Derived from the pore size distribution graph (Figure 2B) obtained from the NL-DFT analysis of the adsorption isotherm, employing a cylindrical pore geometry and considering the model "N2 - Cylindrical Pores- Oxides Surfaces" present in the Microactive software.
[h] Derived from the pore size distribution graph (Figure 2B) obtained from the NL-DFT analysis of the adsorption isotherm, employing a slit pore geometry and considering the model "CO2 – GCMC Carbon" present in the Microactive software.

4. *In situ* IR spectroscopy

IR spectroscopy was performed on a Bruker Vertex 70 instrument equipped with a MCT (mercury cadmium tellurium) cryo-detector. All the spectra were recorded in a spectral range of 4500-250 cm^{-1} with a resolution of 2 cm^{-1} and an average of 32 scans. Before the analysis, F4_MIL-140A(Ce), as self-supported pellet, was activated in vacuum at 393 K for 12 h.

The study of the interaction of F4_MIL-140A(Ce) with H_2O was carried out sending incremental doses of H_2O (from 0.1 to 20 mbar) on the activated sample (see Figure3 in the main text).

Both CO and N_2 interactions with the sample were studied by dosing a proper amount of gas (~60 mbar) into the cell and cooling down to 77 K with liquid nitrogen. The spectra reported for both the experiments were collected in outgassing through subsequent expansions until complete degas was achieved (Figure S16).

The sample was completely evacuated at 393 K overnight (Figure 3) and spectra in contact with CO or N_2 were recorded at 77 K. The interaction of CO with F4_MIL-140A(Ce) gives rise to three main bands at 2155, 2130 and 2106 cm^{-1}. The most intense band at 2155 cm^{-1} can be assigned to CO adsorbed on Ce^{4+} sites.[4] Bands at similar frequencies have also been attributed to the interaction of CO with mildly acidic OH groups.[5,6] However, the absence of changes in the OH stretching region at 3645 cm^{-1} suggests that the latter hypothesis is not realistic (see inset of figure S16). The weak band at 2130 cm^{-1} is tentatively assigned to CO interacting with Ce^{3+} sites, whose presence in F4_MIL-140A(Ce) in small amounts was previously revealed,[7] although another hypothesis is the presence of physisorbed CO into the pores which is usually reported to be in the 2145-2131 cm^{-1} region.[5,8] Since the rate of disappearance of the band at 2130 cm^{-1} is the same as that of the 2155 cm^{-1} band upon CO desorption, and this is not the first signal that starts disappearing, the latter hypothesis is to be considered unlikely. The band at 2106 cm^{-1} is due to the isotopic shift of the ^{13}C-^{16}O interacting with Ce^{4+}. The ratio between the band at 2155 cm^{-1} and this latter one is 1.023, which is nearly equivalent to the isotopic shift factor reported in the literature.[9]

N_2 is a much weaker basic probe than CO and becomes IR active due to a decrease in symmetry caused by the interaction with Brønsted or Lewis acid sites. The interaction of N_2 at 77 K with F4_MIL-140A(Ce) causes the appearance of a band at 2328 cm^{-1}, whose assignment is not straightforward, due to the few data reported in literature. Bands in the range 2332-2328 cm^{-1} are usually assigned to N_2 interacting with hydroxyls and are accompanied by the appearance of signals in the O-H stretching region.[10] Since no significant changes are observed in this latter region, the 2328 cm^{-1} band probably testifies the interaction of N_2 with Ce^{4+} acidic sites (Figure S16). This last interpretation is in agreement with what previously assumed for CO bands and is a proof of a specific interaction of N_2 with the sample. The 2328 cm^{-1} value is in fact comparable to those observed for N_2 adsorbed on MIL-100(Cr) (2290 cm^{-1})[11] and $V_2Cl_{2.8}$(btdd) (2342 cm^{-1}).[12] This result clearly demonstrates N_2 is not an inert molecular probe for this MOF with open metal sites and it could be responsible for the higher BET surface area measured with N_2 than with Ar.

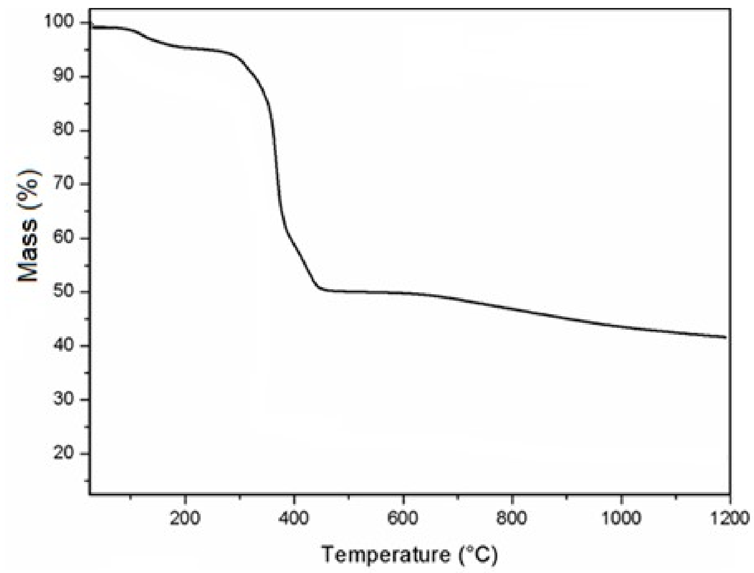

Figure S15. Thermogravimetric curve of F4_MIL-140A(Ce) under oxidative atmosphere, displaying a 4.8% weight loss below 200 °C, corresponding to one water molecule per formula unit, *i.e.*, CeO(OOC-C_6F_4-COO)·H_2O (FW = 410 g mol^{-1}; calculated H_2O = 4.4%) The loading of H_2O, calculated on the basis of adsorption of one molecule per formula unit on the evacuated MOF (FW = 392) corresponds to 2.56 mmol g^{-1}.

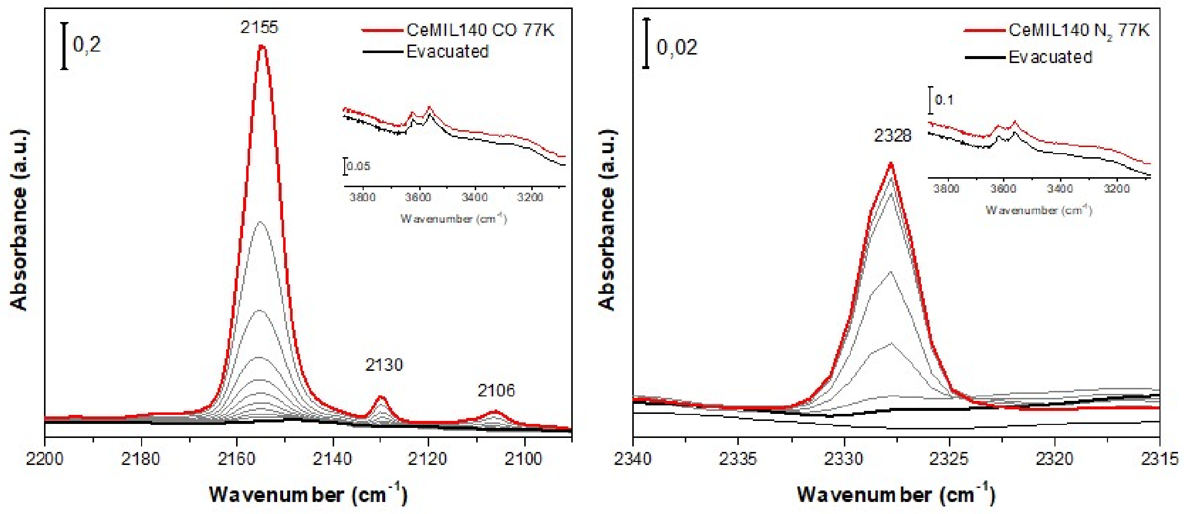

Figure S16. IR spectra of F4_MIL-140A(Ce) after adsorption of 60 mbar of CO at 77 K (left) and after adsorption of 60 mbar of N_2 at 77 K (right) and following outgassing. The spectra were plotted subtracting the spectrum of the evacuated material. In the inset the row data of the hydroxyl region is shown.

5. Periodic density functional theory (DFT) simulations

Vibrational and energetic features of F4_MIL-140A(Ce), also in presence of relevant adsorbates, were simulated at the DFT level of theory with the CRYSTAL17 code.[13,14] In detail, the B3LYP hybrid GGA functional[15,16] was exploited in conjunction with a double-ζ quality basis set (C and O from ref. [17]; F from ref. [18] and Ce from ref. [19]). An effective core potential was adopted to describe the 26 electrons located in the inner shells of Ce. A larger Ahlrichs TZV2p basis[17] described atoms belonging to adsorbed molecules (H_2O, CO_2, CO and N_2). Dispersive interactions were empirically included in the Hamiltonian according to the Becke-Johnson dumped version of the Grimme's D3 scheme.[20,21] The truncations for the mono- and bi-electronic integral (TOLINTEG) were set to {7 7 7 7 14}. The sampling in the reciprocal space (SHRINK) was set to {2 2}, for a total of 6 irreducible k-points sampled in the 1st Brillouin zone. Such computational setup provided a satisfactory structural and electronic description of Ce-MOFs.[22] Initially, the structure of the dehydrated F4_MIL-140A(Ce) (experimental structure as initial model) was geometry optimised. Symmetry was fully exploited in the calculations. Subsequently, adsorbed molecules were introduced and the obtained structures optimised. H_2O was put in direct interaction with the exposed Ce site of the evacuated MOF through a lone-pair of the O atom of the molecule (initial Ce-O distance was set to 2.2 Å). CO_2 interaction with F4_MIL-140A(Ce) was modelled considering two distinct adsorption sites: i) in direct interaction with Ce, by forming a linear adduct (initial Ce-O distance 2.4 Å); ii) positioned within the MOF channels, as proposed by Zhao and coworkers.[23] Vibrational frequencies and IR intensities were computed for all relaxed structures and the absence of imaginary modes verified (i.e. all relaxed models are energy minima). Enthalpies and Gibbs free energies, computed at T = 298.15 K and p = 1013 mbar, were also obtained for the minima structures. Adsorption energies (ΔE), enthalpies (ΔH) and Gibbs free energies (ΔG) per adsorbed molecule were calculated as it follows:

$$\Delta X = \frac{X_{MOF+mol} - X_{MOF} - 4 \cdot X_{mol}}{4} \quad \text{with } X = E, H, or\ G$$

Where $X_{MOF+mol}$ refers to the F4_MIL-140A(Ce) + molecule adduct structure, X_{MOF} to the dehydrated MOF and X_{mol} to the isolated molecule. The obtained values were corrected for the basis set superposition error (BSSE) through the counterpoise method.[24]

Table S3. Cell parameters for the DFT-optimised structures of F4_MIL-140A(Ce), as such and interacting with H_2O and CO_2 (the latter, both directly with the metal site or with the microporous channel).

Model	*a* (Å)	*b* (Å)	*c* (Å)	*β (°)*
**F4_MIL-140A(Ce)**	25.928	11.522	8.169	102.3
**+H_2O**	26.269	11.604	7.950	96.9
**+CO_2 (Ce)**	26.015	11.515	8.125	98.9
**+CO_2 (channel)**	25.870	11.544	8.196	102.5
**+ CO**	26.157	11.550	8.063	103.1
**+ N_2**	26.026	11.550	8.122	103.1

DFT-optimised structure of evacuated F4_MIL-140A(Ce)

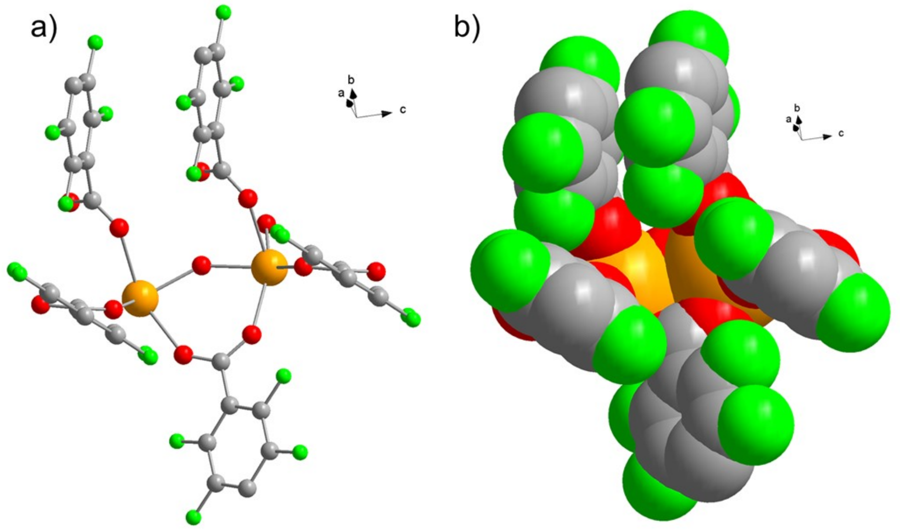

Figure S17. Ball-and-stick (left) and space-filling (right) representation of the DFT-optimised local environment of evacuated F4_MIL-140A(Ce) in the proximity of the free adsorption site. Colour code: Ce, orange; F, green; C, grey; O, red.

DFT-optimised structure of as-synthesised F4_MIL-140A(Ce)

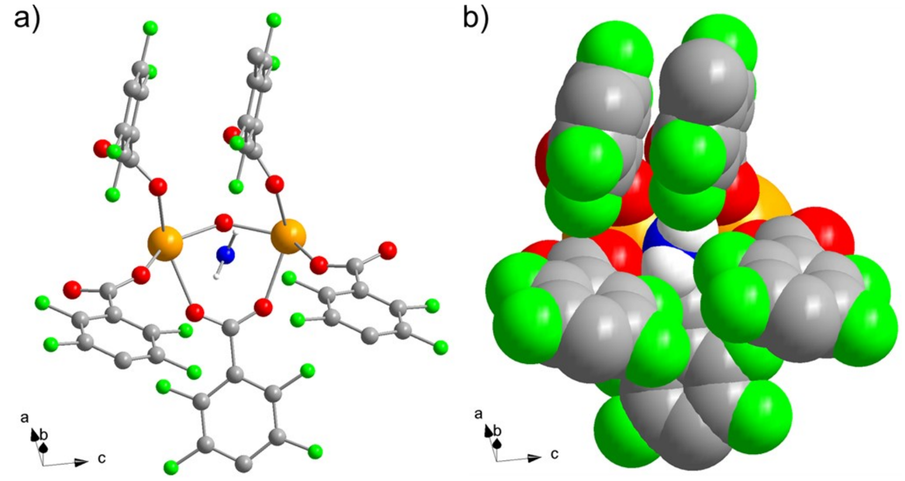

Figure S18. Ball-and-stick (a) and space-filling (b) representation of the DFT-optimised structure of as-synthesised F4_MIL-140A(Ce). Colour code: Ce, orange; F, green; C, grey; O, red; O belonging to H_2O, blue; H, white.

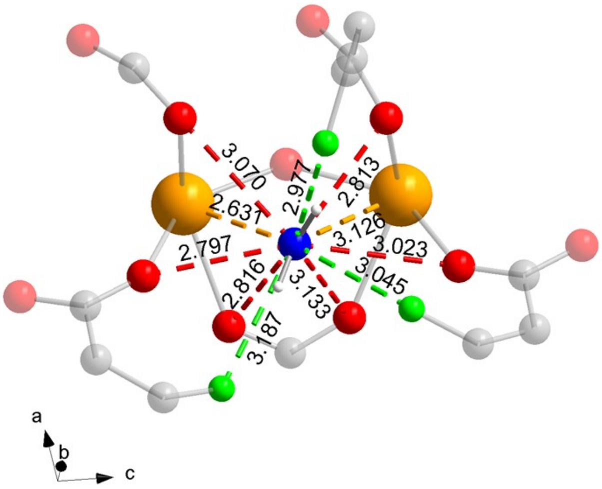

Figure S19. Local environment around the water molecule in the DFT-optimised structure of as-synthesised F4_MIL-140A(Ce). A threshold distance of 3.5 Å between the O atom of water and surrounding atoms was chosen to identify possible interactions, represented as dashed lines having the same colour of the interacting atom. Non-interacting atoms are depicted as transparent. Colour code: Ce, orange; F, green; C, grey; O, red; O belonging to H_2O, blue; H, white.

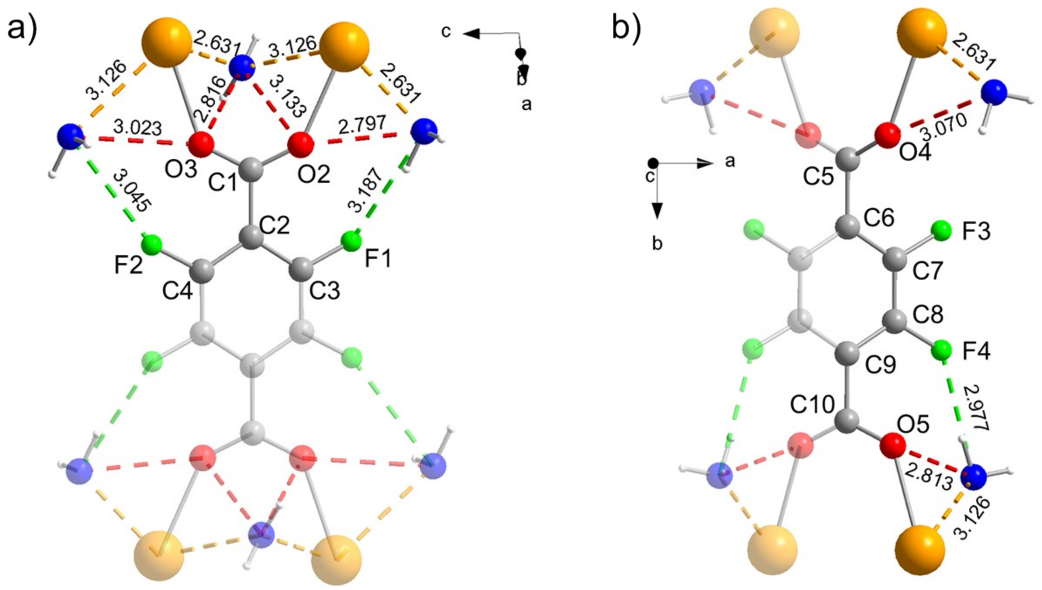

Figure S20. Local environment around linker A (a) and linker B (b) in the DFT-optimised structure of as-synthesised F4_MIL-140A(Ce). A threshold distance of 3.5 Å between the O atom of water and surrounding atoms was chosen to identify possible interactions, represented as dashed lines having the same colour of the interacting atom. Symmetry-generated atoms belonging to the linkers and other atoms either bonded or interacting with them are depicted as transparent. Colour code: Ce, orange; F, green; C, grey; O, red; O belonging to H_2O, blue; H, white.

DFT-optimised structure of CO_2-loaded F4_MIL-140A(Ce)

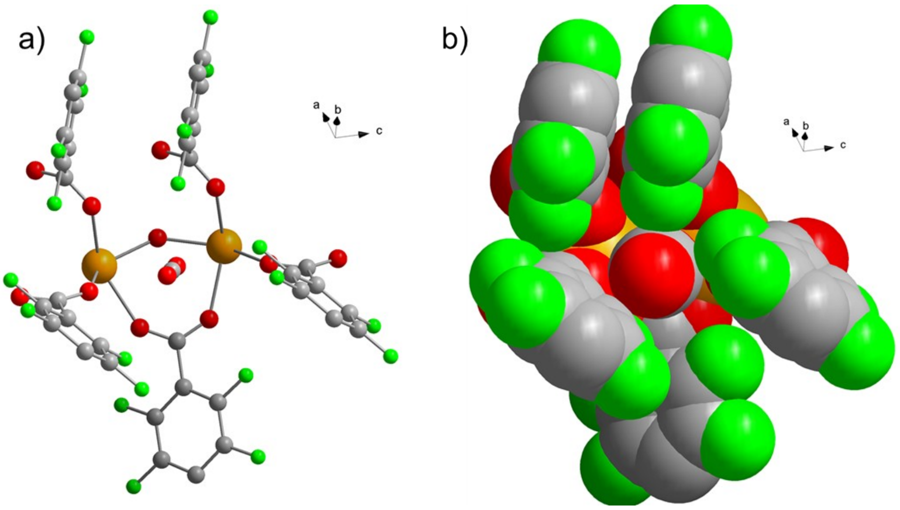

Figure S21. Ball-and-stick (a) and space-filling (b) representation of the DFT-optimised structure of CO_2-loaded F4_MIL-140A(Ce). Colour code: Ce, orange; F, green; C, grey; O, red.

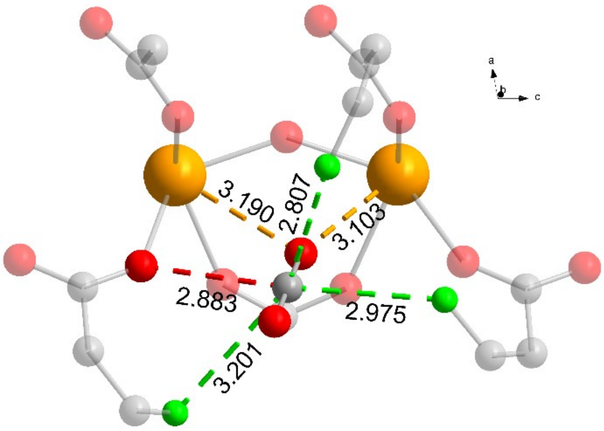

Figure S22. Local environment around the CO_2 molecule in the DFT-optimised structure of CO_2-loaded F4_MIL-140A(Ce). A threshold distance of 3.5 Å between the C and O atoms of CO_2 and surrounding atoms was chosen to identify possible interactions, represented as dashed lines having the same colour of the interacting atom. Non-interacting atoms are depicted as transparent. Colour code: Ce, orange; F, green; C, grey; O, red.

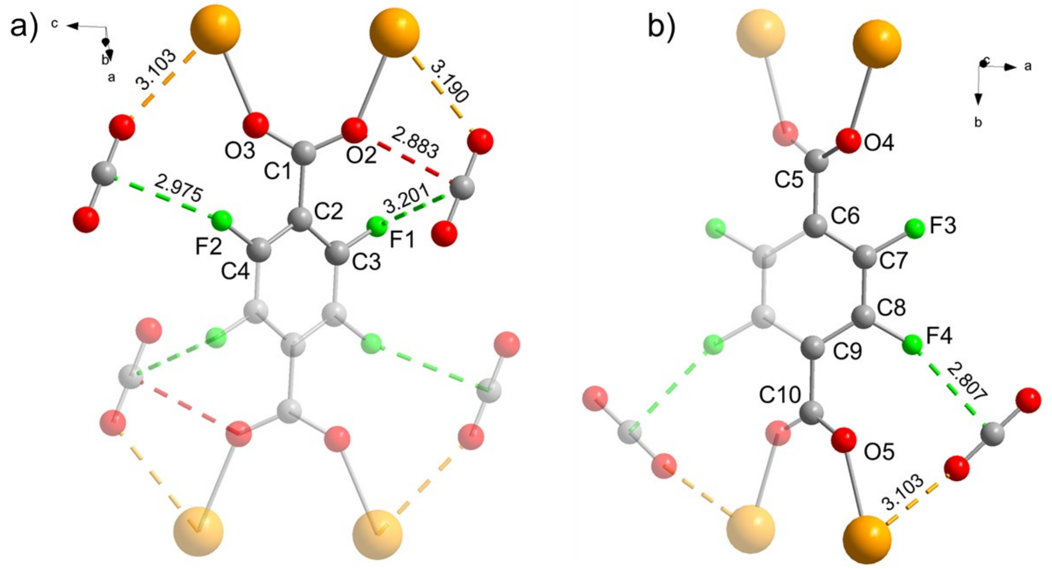

Figure S23. Local environment around linker A (a) and linker B (b) in the DFT-optimised structure of CO_2-loaded F4_MIL-140A(Ce). A threshold distance of 3.5 Å between the C and O atoms of CO_2 and surrounding atoms was chosen to identify possible interactions, represented as dashed lines having the same colour of the interacting atom. Symmetry-generated atoms belonging to the linkers and other atoms either bonded or interacting with them are depicted as transparent. Colour code: Ce, orange; F, green; C, grey; O, red.

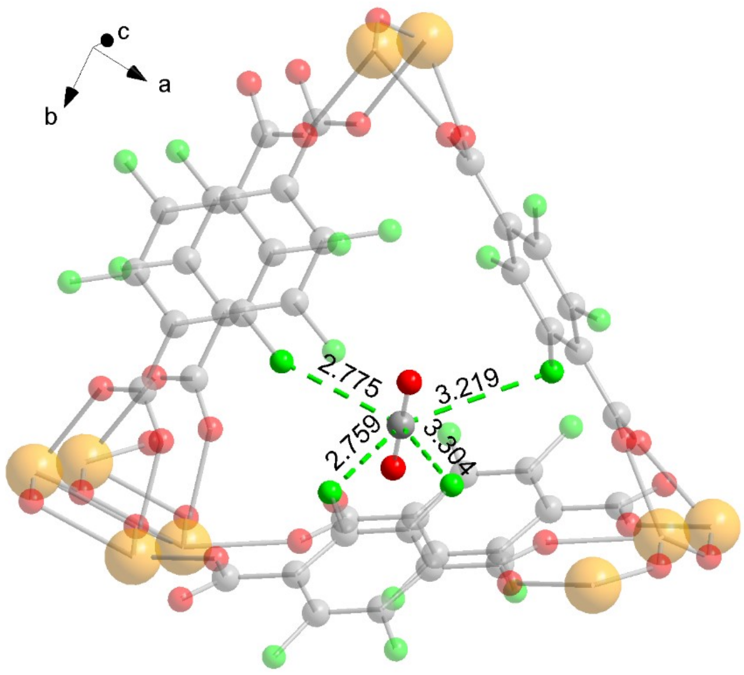

Figure S24. Local environment around the CO_2 molecule in the DFT-optimised structure of F4_MIL-140A(Ce) with CO_2 adsorbed at the “channel” site. A threshold distance of 3.5 Å between the C atom of CO_2 and surrounding F atoms was chosen to identify possible interactions, represented as dashed lines having the same colour of the interacting atom. Non-interacting atoms are depicted as transparent. Colour code: Ce, orange; F, green; C, grey; O, red.

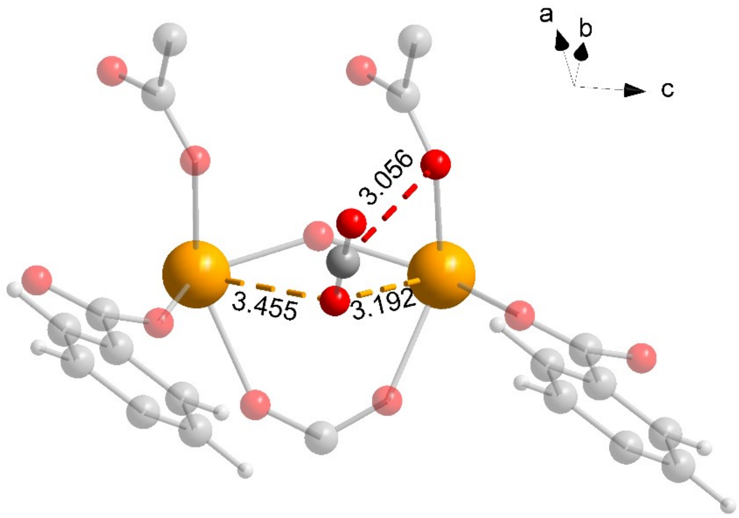

Figure S25. Local environment around the CO_2 molecule in the DFT-optimised structure of CO_2-loaded MIL-140A(Ce). A threshold distance of 3.5 Å between the C and O atoms of CO_2 and surrounding atoms was chosen to identify possible interactions, represented as dashed lines having the same colour of the interacting atom. Non-interacting atoms are depicted as transparent. Colour code: Ce, orange; C, grey; O, red; H, white.

6. *In situ* powder X-ray diffraction (PXRD)

The *in situ* PXRD study described took place at Beamline P02.1 (PETRA III, DESY, Hamburg).[25] PXRD patterns were collected using 60.0 keV (0.207124 Å) radiation. The sample was held in a 0.5 mm internal diameter glass capillary, which was mounted on a custom-built spinner device which allows for rotating a gas filled capillary mounted on a commercial Huber goniometer head by 360° forth and back. The temperature of the sample was controlled between 25 and 140 °C with an Oxford Hot-Air Blower. The beamline was equipped with a Perkin Elmer XRD1621 CN3 - EHS detector (200×200 μm^2 pixel size, 2048 x 2048 pixel area). The detector was positioned at 1000 mm from the sample stage. Each PXRD pattern was collected in the 0.0071-16.3041 °2θ range, with a 0.0067 °2θ step size and a total acquisition time of 30 s. The resulting 2D images were azimuthally integrated to 1D diffraction patterns using the software Fit2D.[26]

The data were analysed by Rietveld refinement as implemented in the software TOPAS 6.0.[27] Wavelength, instrument peak shape parameters and zero-point error were refined based on data collected on LaB_6 standard material. First, a Pawley refinement was carried out to model background, profile shape parameters and lattice parameters. In both cases, a slightly better result was obtained when refining using a $P2_1/c$ space group, rather than the more symmetric $C2/c$ space group observed for the as-synthesised MOF and used for all DFT simulations. However, given the very small differences in Rwp (2.38 in $C2/c$ vs 2.03 in $P2_1/c$ for the evacuated MOF; 2.50 in $C2/c$ vs 2.16 in $P2_1/c$ for the CO_2 loaded MOF), we decided to proceed with Rietveld refinement using the highest symmetry space group, which contains half as many crystallographically independent atoms and requires to refine less parameters. This choice is also supported by SSNMR data, which show that only two crystallographically independent linkers exist.

Rietveld refinements of the dehydrated and CO_2 loaded forms of F4_MIL-140A(Ce) were performed using structural models obtained from the DFT optimisations described above. Rietveld refinement was performed to model the atomic coordinates and atomic displacement parameters. For the CO_2 loaded F4_MIL-140A(Ce), the pattern collected at 40 °C under 1188 mbar CO_2 was refined. The aromatic ring of linker B was modelled by employing a rigid group. To correctly describe the geometry of the aromatic ring, one dummy atom named XX, whose occupancy factor was set to zero, was employed in the rigid group and placed at the centre of the aromatic ring. The position of XX was restrained to the special position (0, y, 0.25) by fixing the translational parameters of the rigid group along the *x* and *z* axis. Rotational parameters along the *x* and *z* axis were kept fixed at 90 and 0°, respectively, whereas rotation along the *y* axis was left free to refine. All the other atoms were refined individually, using 20 distance restraints and 16 angle restraints for each structure. To correctly describe the geometry of the aromatic ring of linker A, another dummy atom named YY, whose occupancy factor was also set to zero, was employed. The position of YY was restrained to the special position (0.25, 0.25, 0.5), and three distance and three angle restraints were applied that involved this atom. In the case of the CO_2 loaded MOF, an additional restraint was applied to ensure flatness of the aromatic ring belonging to linker A. The atomic displacement parameter for Ce was refined independently, while those of the light atoms were constrained to the same value. In the CO_2 loaded MOF, the occupancy of the CO_2 molecule was set to 0.9, in agreement with the observed loading at 40 °C under 1188 mbar CO_2 (2.25 mmol, versus a loading at saturation of 2.5 mmol) and the atomic displacement parameters of the constituting C and O atoms were constrained to refine to the same value, independently from those of the light atoms belonging to the framework. At the end of the refinement, all the parameters were refined together until convergence. Details of the refinements are reported in Table S4.

Table S4. Crystallographic parameters and refinement details of the different forms of F4_MIL-140A(Ce).

	As-synthesised	Evacuated	CO_2-loaded
**Temperature**	298 K	298 K	313 K
**Refinement method**	Pawley	Rietveld	Rietveld
**Empirical formula**	$CeO_6C_8F_4H_2$	$CeO_5C_8F_4$	$CeO_{6.8}C_{8.9}F_4$
**Formula weight**	410.19	392.19	431.79
**Wavelength/Å**	0.207124	0.207124	0.207124
**Crystal system**	monoclinic	monoclinic	monoclinic
**Space group**	*C*2/*c*	*C*2/*c*	*C*2/*c*
***a*/Å**	25.738(6)	25.578(3)	25.548(3)
***b*/Å**	11.578(2)	11.509(1)	11.496(1)
***c*/Å**	8.110(5)	8.178(1)	8.181(1)
**β/°**	96.35(5)	103.99(2)	97.68(1)
**V/Å^3**	2402(2)	2336.0(5)	2381.2(6)
**Z**	8	8	8
**Calc. density/g cm^{-3}**	2.27	2.23	2.41
**R_p**	0.0128	0.0362	0.0399
**R_{wp}**	0.0197	0.0485	0.0523
**R_F2**	-	0.0234	0.0244
**GOF**	3.752	3.165	3.375
**CCDC Deposition number**	-	2212834	2212835

Evacuation process

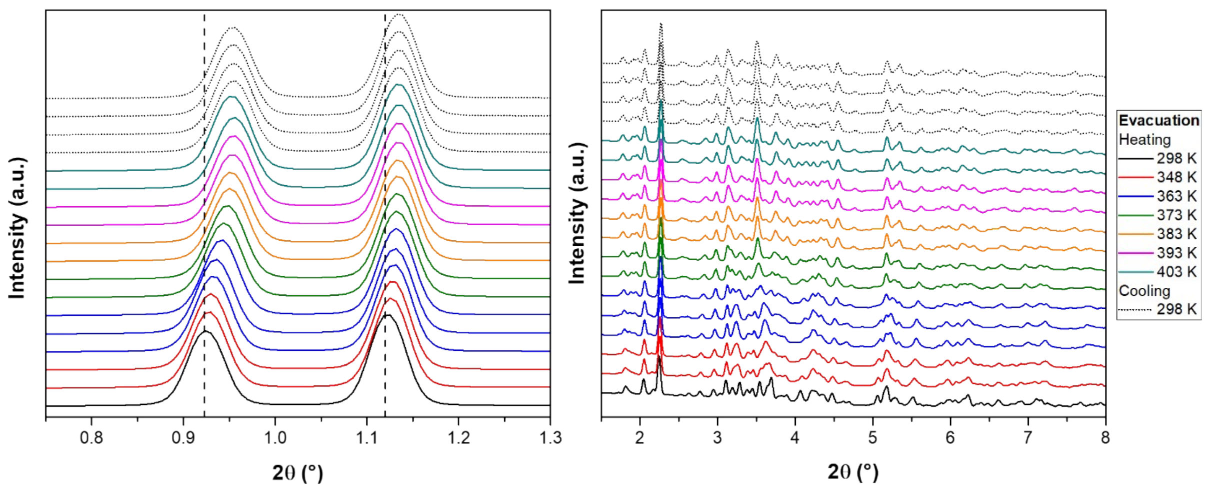

Figure S26. Low angle region (left) and high angle region (right) of the PXRD patterns of F4_MIL-140A(Ce) collected at temperatures in the range comprised between 298 and 403 K during the evacuation step. The sample was held under dynamic vacuum in the range of 10^{-5}-10^{-6} bar. Different patterns at the same temperature were collected with a 5 min gap. The dashed vertical lines mark the position of the 200 (leftmost) and 110 (rightmost) reflections in the as-synthesised MOF.

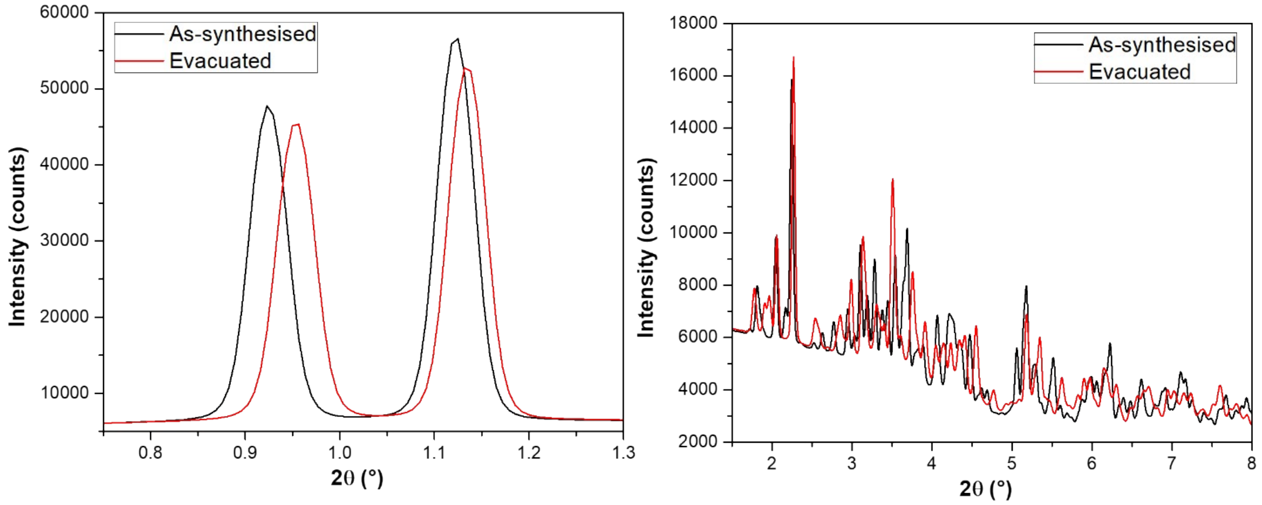

Figure S27. Comparison between the low angle (left) and high angle (right) regions of the PXRD patterns of as-synthesised (black) and evacuated (red) F4_MIL-140A(Ce).

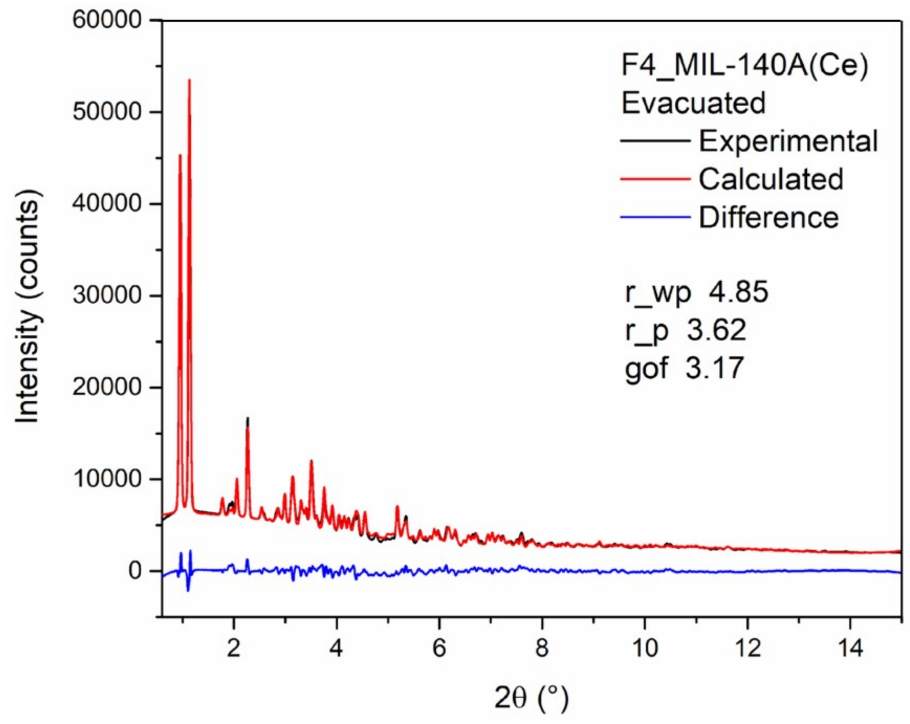

Figure S28. Rietveld plot for the evacuated F4_MIL-140A(Ce).

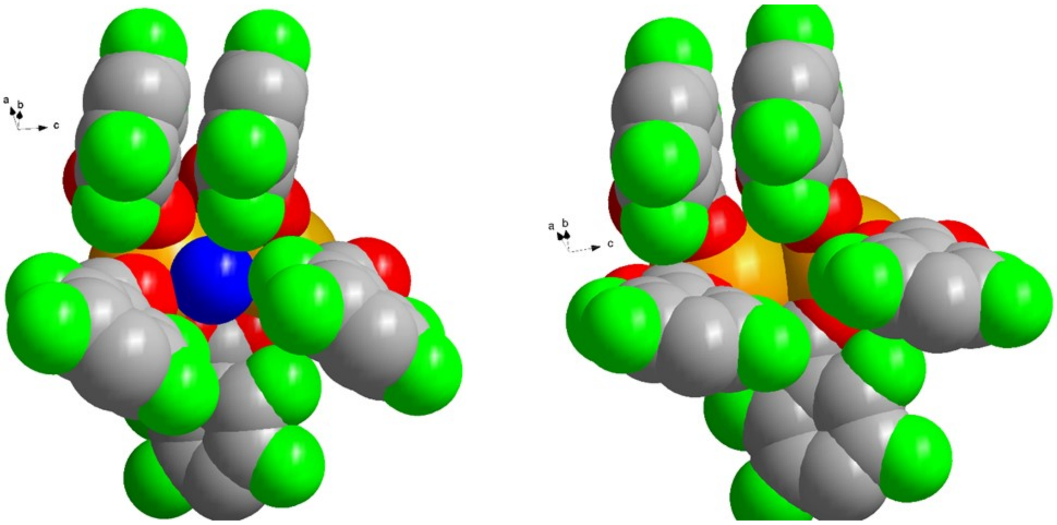

Figure S29. Comparison between the space-filling representation of Figures 5b (left) and 5d (right) in the main text. Colour code: Ce, orange; F, green; C, grey; O, red.

CO_2 adsorption

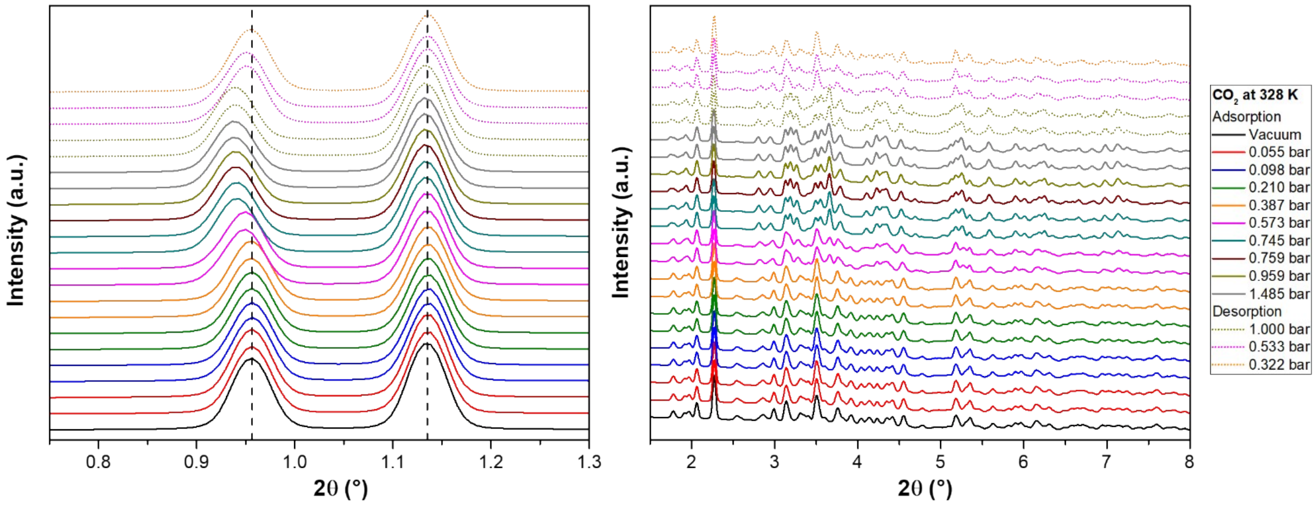

Figure S30. Low angle region (top) and high angle region (bottom) of the PXRD patterns of F4_MIL-140A(Ce) collected at 328 K under CO_2 pressures comprised between 0 and 1.485 bar. Different patterns at the same pressure were collected with a 5 min gap. The dashed vertical lines mark the position of the 200 (leftmost) and 110 (rightmost) reflections in the evacuated MOF.

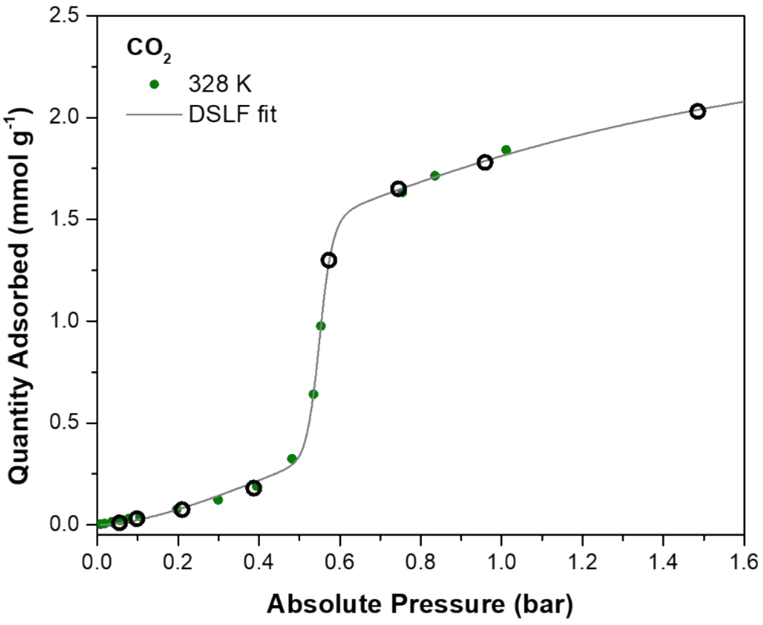

Figure S31. Localisation of the CO_2 pressures investigated in adsorption during the PXRD experiment carried out at 328 K (black circles) on the respective adsorption isotherm.

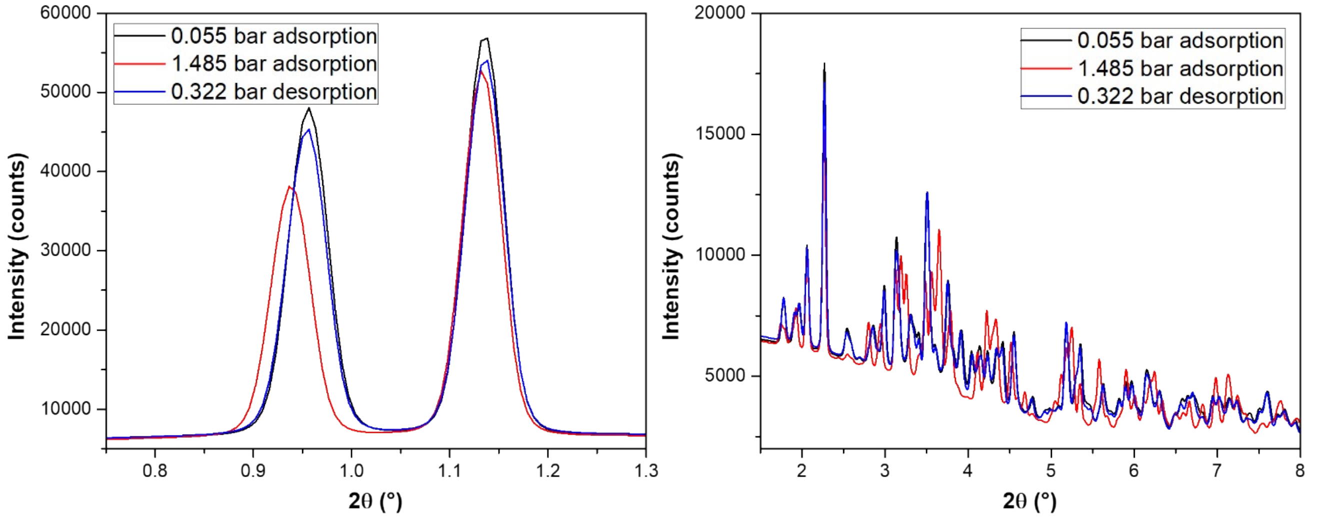

Figure S32. Comparison between the low angle (left) and high angle (right) regions of the PXRD patterns of F4_MIL-140A(Ce) collected at 328 K under 0.055 bar (black) and 1.485 bar (red) of CO_2 in adsorption and under 0.322 mbar (blue) of CO_2 in desorption.

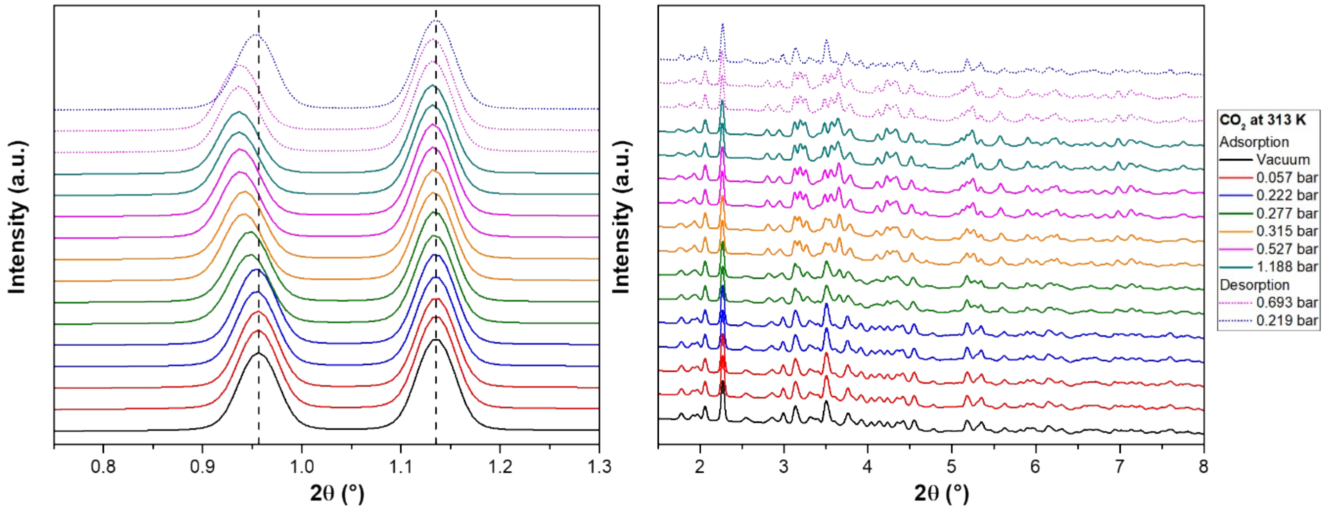

Figure S33. Low angle region (left) and high angle region (right) of the PXRD patterns of F4_MIL-140A(Ce) collected at 313 K under CO_2 pressures comprised between 0 and 1.188 bar. Different patterns at the same pressure were collected with a 5 min gap. The dashed vertical lines mark the position of the 200 (leftmost) and 110 (rightmost) reflections in the evacuated MOF.

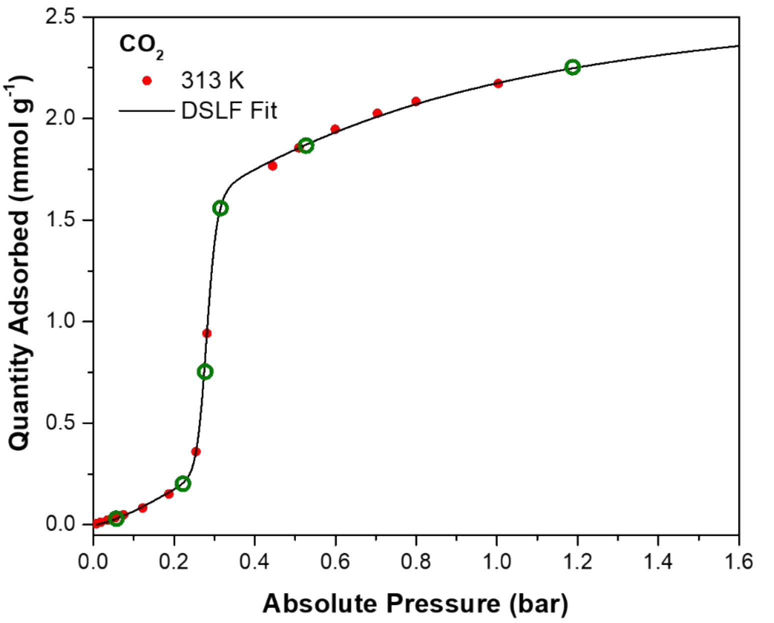

Figure S34. Localisation of the CO_2 pressures investigated in adsorption during the PXRD experiment carried out at 313 K (green circles) on the respective adsorption isotherm.

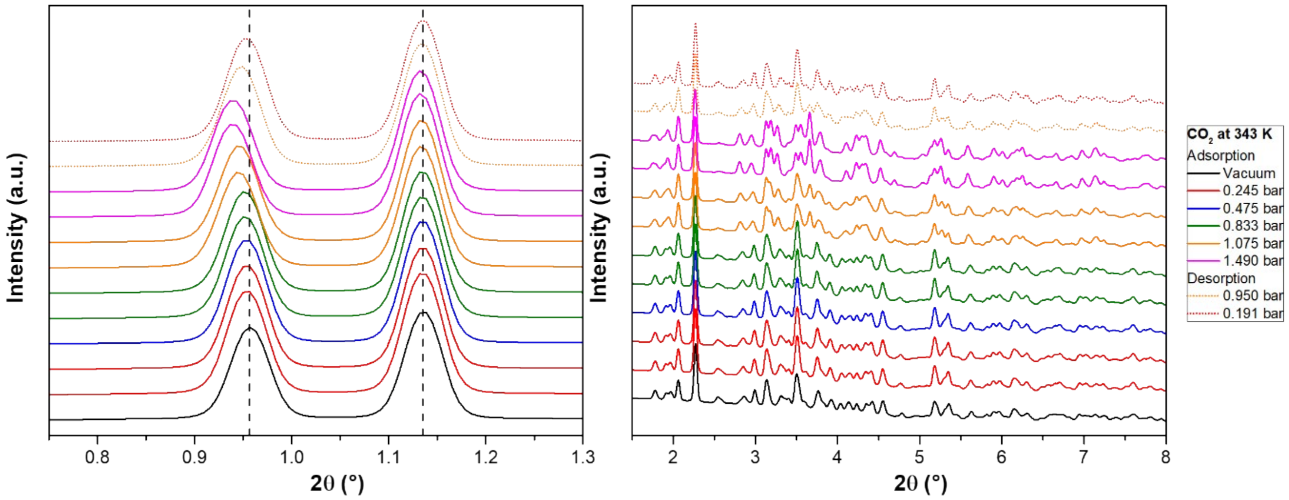

Figure S35. Low angle region (left) and high angle region (right) of the PXRD patterns of F4_MIL-140A(Ce) collected at 343 K under CO_2 pressures comprised between 0 and 1.490 bar. Different patterns at the same pressure were collected with a 5 min gap. The dashed vertical lines mark the position of the 200 (leftmost) and 110 (rightmost) reflections in the evacuated MOF.

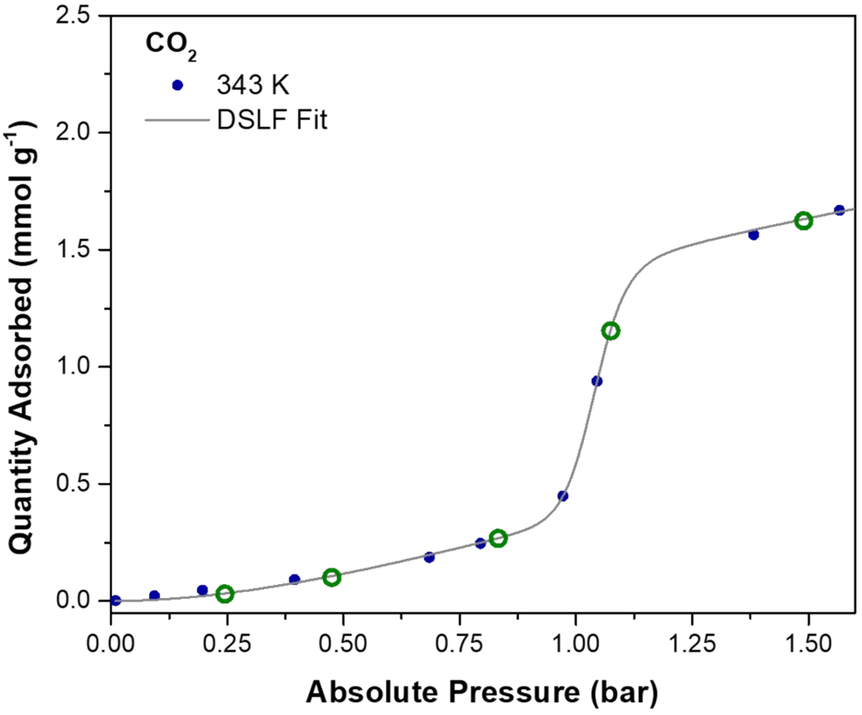

Figure S36. Localisation of the CO_2 pressures investigated in adsorption during the PXRD experiment carried out at 343 K (green circles) on the respective adsorption isotherm.

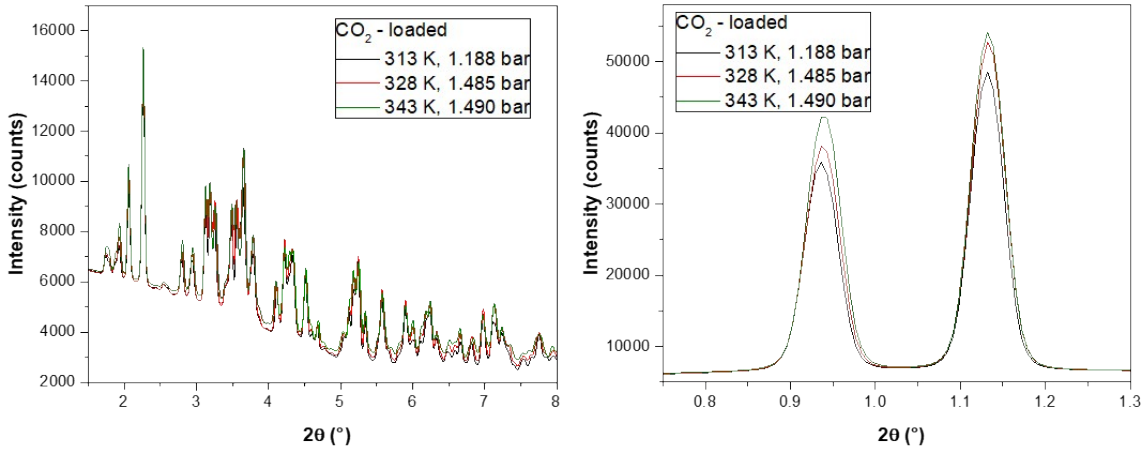

Figure S37. Comparison between the low angle (left) and high angle (right) regions of the PXRD patterns of F4_MIL-140A(Ce) loaded with CO_2 at 313 K (1.188 bar, black), 328 K (1.485 bar, red) and 343 K (1.490 bar, green).

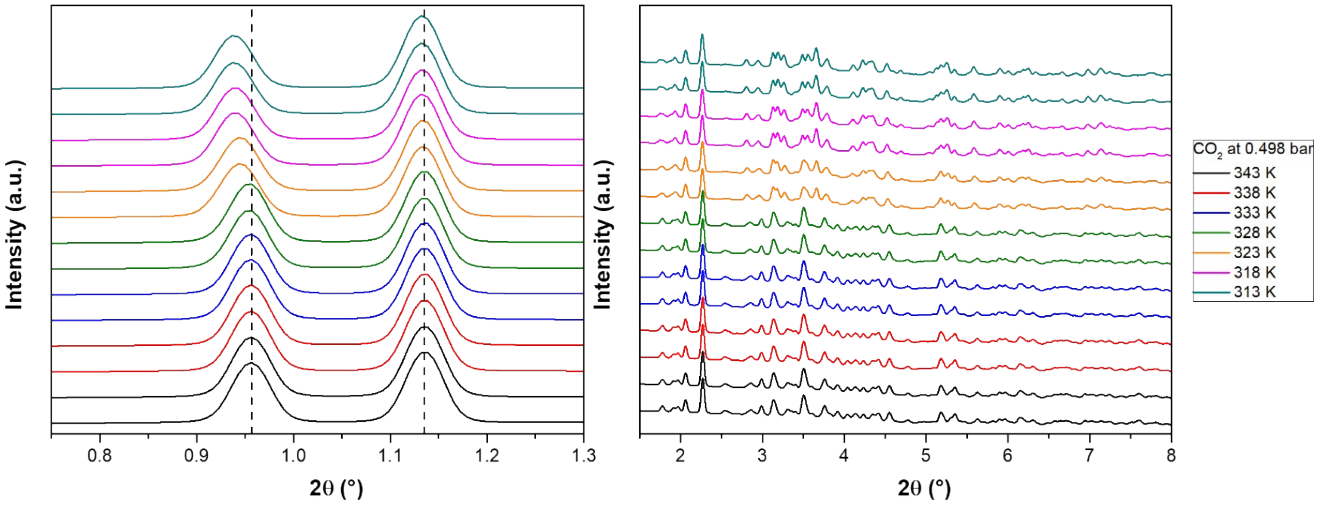

Figure S38. Low angle region (left) and high angle region (right) of the PXRD patterns of F4_MIL-140A(Ce) collected under constant 0.498 bar CO_2 pressure at temperature comprised between 343 and 313 K. Different patterns at the same pressure were collected with a 5 min gap. The dashed vertical lines mark the position of the 200 (leftmost) and 110 (rightmost) reflections in the evacuated MOF.

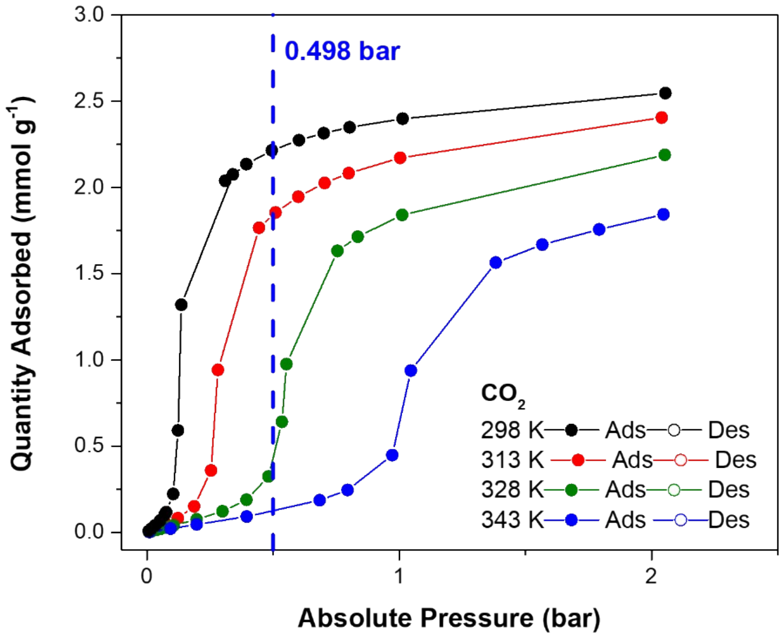

Figure S39. Localisation of the 0.498 bar CO_2 pressure (dashed blue line) used for the isobaric experiment, with respect to the experimental CO_2 adsorption isotherms. The phase transition from evacuated to CO_2-loaded observed from PXRD occurs just below 328 K, as expected.

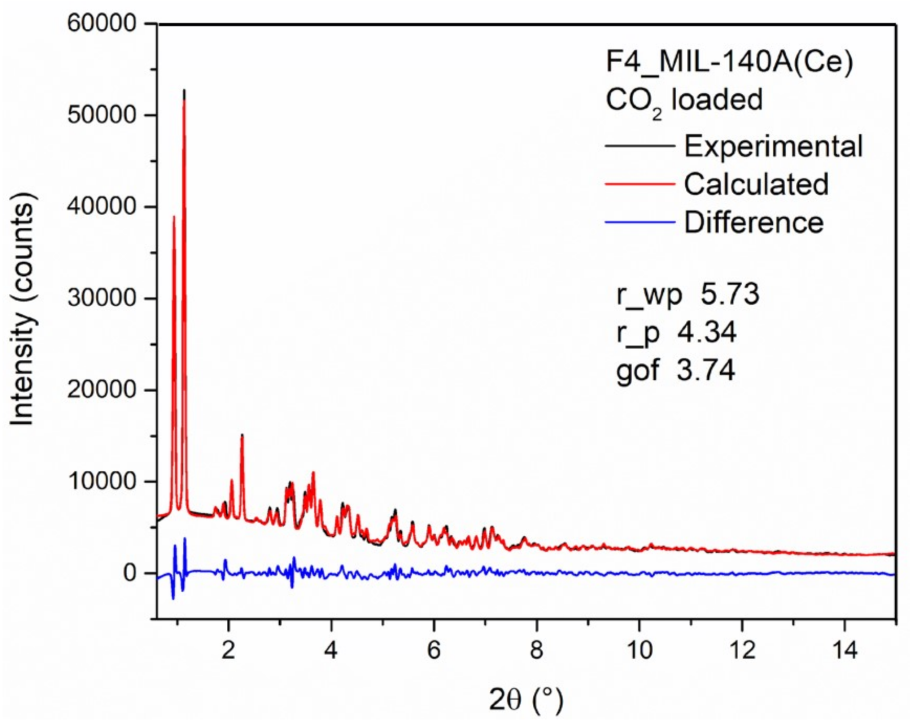

Figure S40. Rietveld plot for CO_2-loaded F4_MIL-140A(Ce) at 313 K.

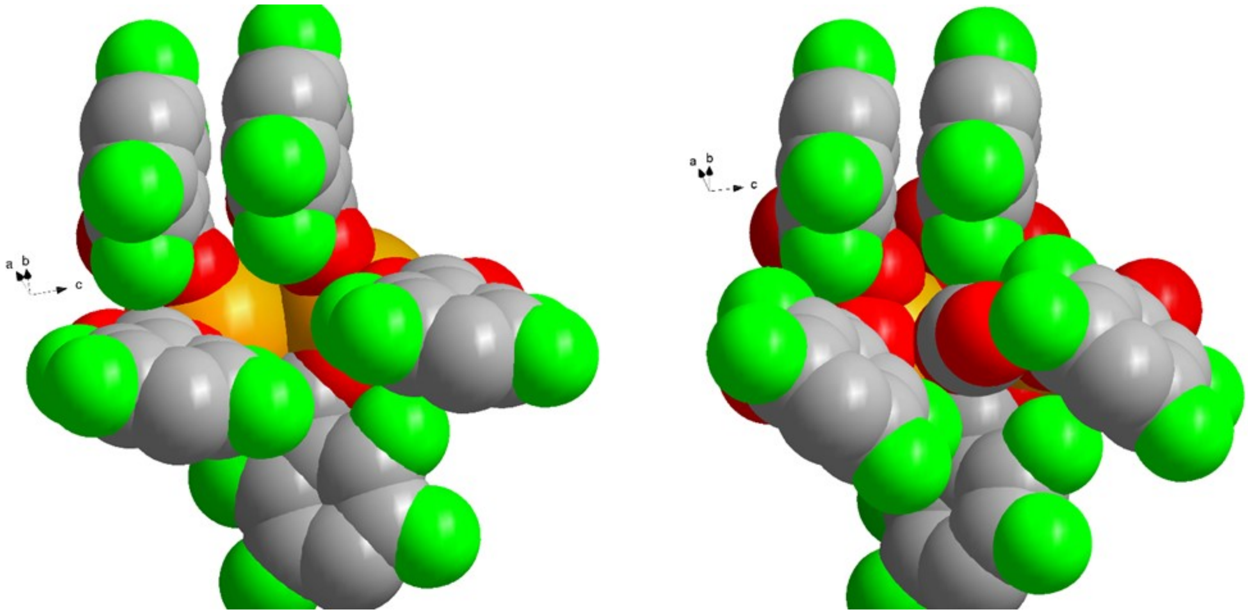

Figure S41. Comparison between the space-filling representation of Figures 5d (left) and 5f (right) in the main text. Colour code: Ce, orange; F, green; C, grey; O, red.

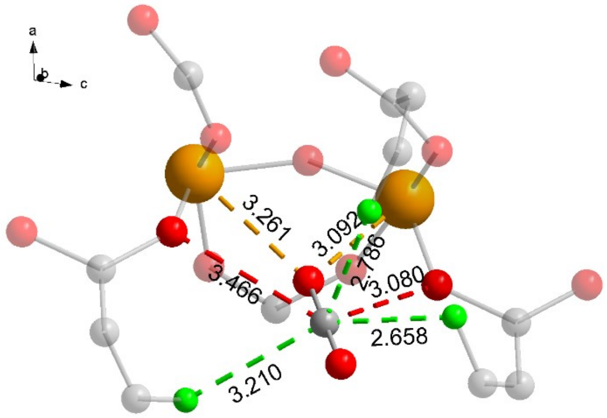

Figure S42. Local environment around the CO_2 molecule in the Rietveld-refined structure of CO_2-loaded F4_MIL-140A(Ce). A threshold distance of 3.5 Å between the C and O atoms of CO_2 and surrounding atoms was chosen to identify possible interactions, represented as dashed lines having the same colour of the interacting atom. Non-interacting atoms are shaded. Colour code: Ce, orange; F, green; C, grey; O, red.

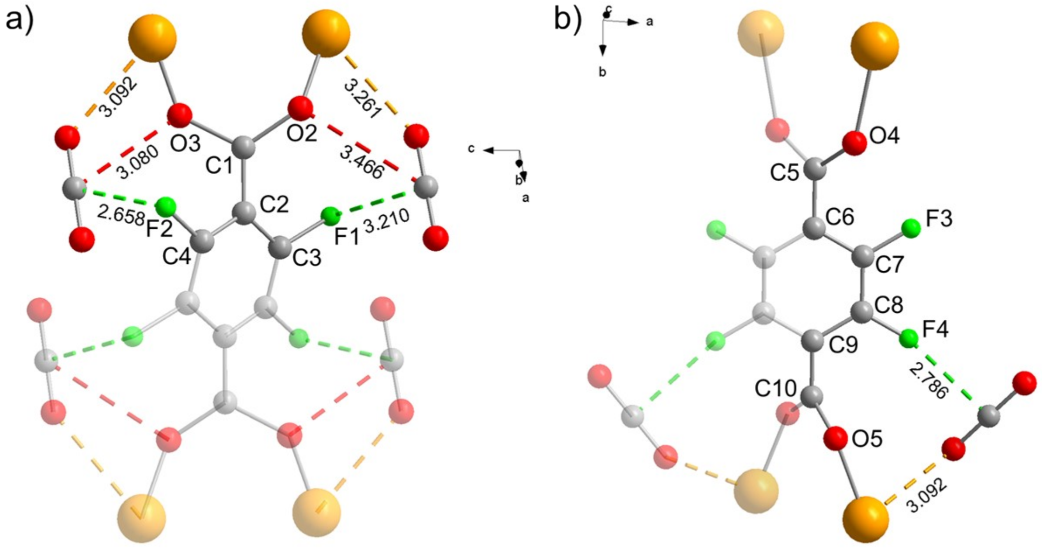

Figure S43. Local environment around linker A (a) and linker B (b) in the Rietveld-refined structure of CO_2-loaded F4_MIL-140A(Ce). A threshold distance of 3.5 Å between the C and O atoms of CO_2 and surrounding atoms was chosen to identify possible interactions, represented as dashed lines having the same colour of the interacting atom. Symmetry-generated atoms belonging to the linkers and other atoms either bonded or interacting with them are depicted as transparent. Colour code: Ce, orange; F, green; C, grey; O, red.

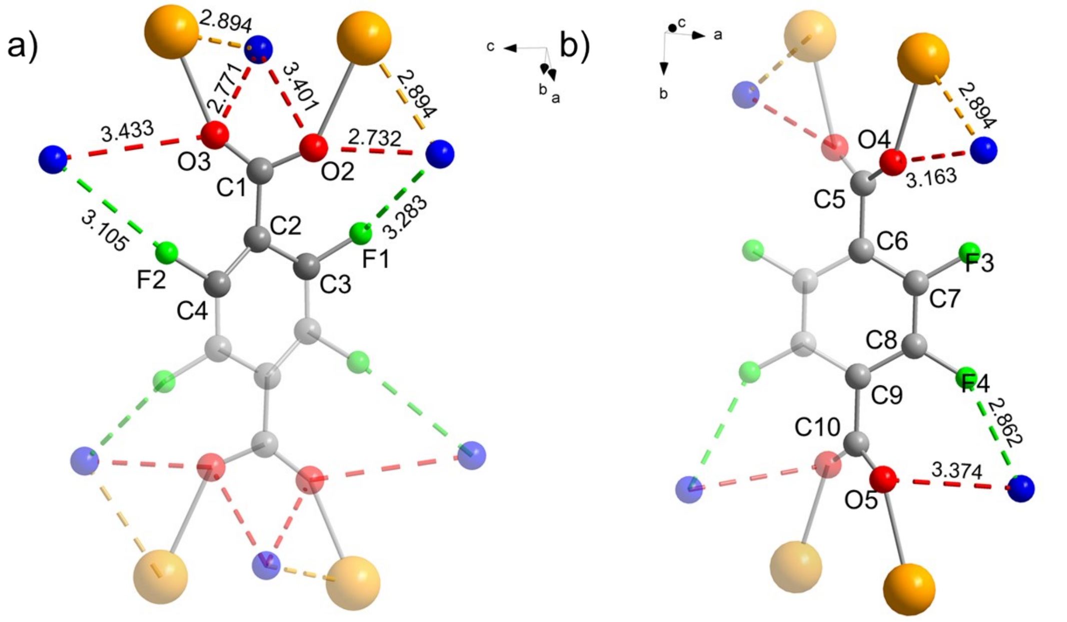

Figure S44. Local environment around linker A (a) and linker B (b) in the Rietveld-refined structure of as-synthesised F4_MIL-140A(Ce). A threshold distance of 3.5 Å between the O atom of water and surrounding atoms was chosen to identify possible interactions, represented as dashed lines having the same colour of the interacting atom. Symmetry-generated atoms belonging to the linkers and other atoms either bonded or interacting with them are depicted as transparent. Colour code: Ce, orange; F, green; C, grey; O, red; O belonging to H_2O, blue. H atoms are not shown because the structure was determined from PXRD data.

Table S5. Torsional angles between the carboxylate groups and the aromatic rings of the two crystallographically independent linkers, as obtained by Rietveld refinement and DFT calculations, for as-synthesised, evacuated and CO_2-loaded F4_MIL-140A(Ce).

State	Method	Torsional angles		
		Linker A	Linker B	
		O2-C1-C2-C3	O5-C10-C9-C8	O4-C5-C6-C7
As-synthesised	Rietveld	-36.3°	58.8°	-56.7°
	DFT	-25.9°	50.2°	-64.9°
Evacuated	Rietveld	-11.0°	36.2°	-50.0°
	DFT	-27.5°	52.6°	-57.0°
CO_2-loaded	Rietveld	-64.0°	70.0°	-52.8°
	DFT	-38.3°	60.6°	-62.2°

7. *In situ* X-ray absorption spectroscopy (XAS)

Ce K-edge XAS data were collected on the BM23[28] beamline of the European Synchrotron Radiation Facility (ESRF) of Grenoble, France. The storage ring was operating in the 32-bunch mode with a target current of 150 mA. The measurements were conducted in transmission mode using a Si(311) double-crystal monochromator. Intensity of the X-ray beam was measured by the means of three ion chambers (30 cm, 1 kV) placed before the sample (I_0, filling 0.28 bar Kr + 1.72 bar He), after the sample (I_1, filling 1.35 bar Kr + 0.65 bar He) and after the CeO_2 reference sample (I_2 filling 1.35 bar Kr + 0.65 bar He). This experimental setup allowed us to reference the energy to the edge of the reference sample and thus calibrate the energy for each spectrum.

Samples were measured in the form of self-supporting pellets, whose optimized weight were calculated with the XAFSmass code.[29] A home-built gas dosing setup equipped with mass-flow controllers allowed us to dose He/CO_2 mixtures at carefully selected amounts in order to collect adsorption isotherms. Total flow was set to 50 mL min^{-1}. The temperature was set to 328 K.

EXAFS data analysis was carried out by the means of the Demeter package[30]: Athena for the raw data treatment (normalization, extraction) and Artemis for fitting of the EXAFS spectra. FEFF6[31] was used to calculate phase shifts and scattering amplitudes.

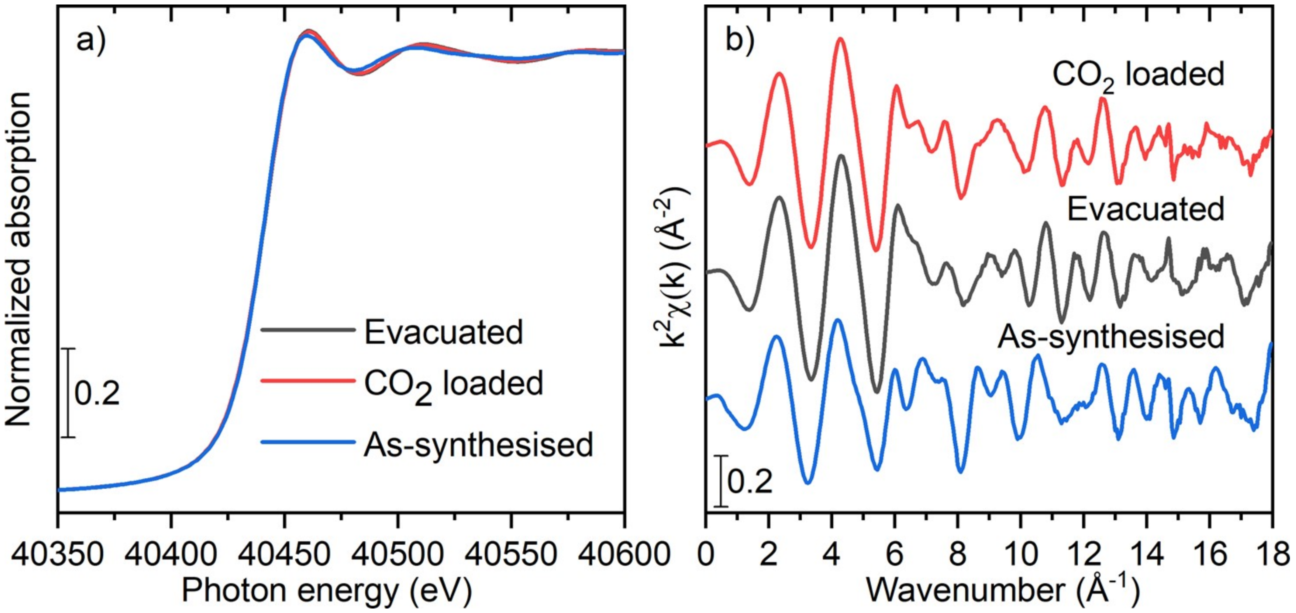

Figure S45. Ce K-edge XANES spectra (left side) and k^2-weighted EXAFS signal (right side) of F4_MIL-140A(Ce) in the as-synthesised (blue line), evacuated (grey) and CO_2-loaded (red) states.

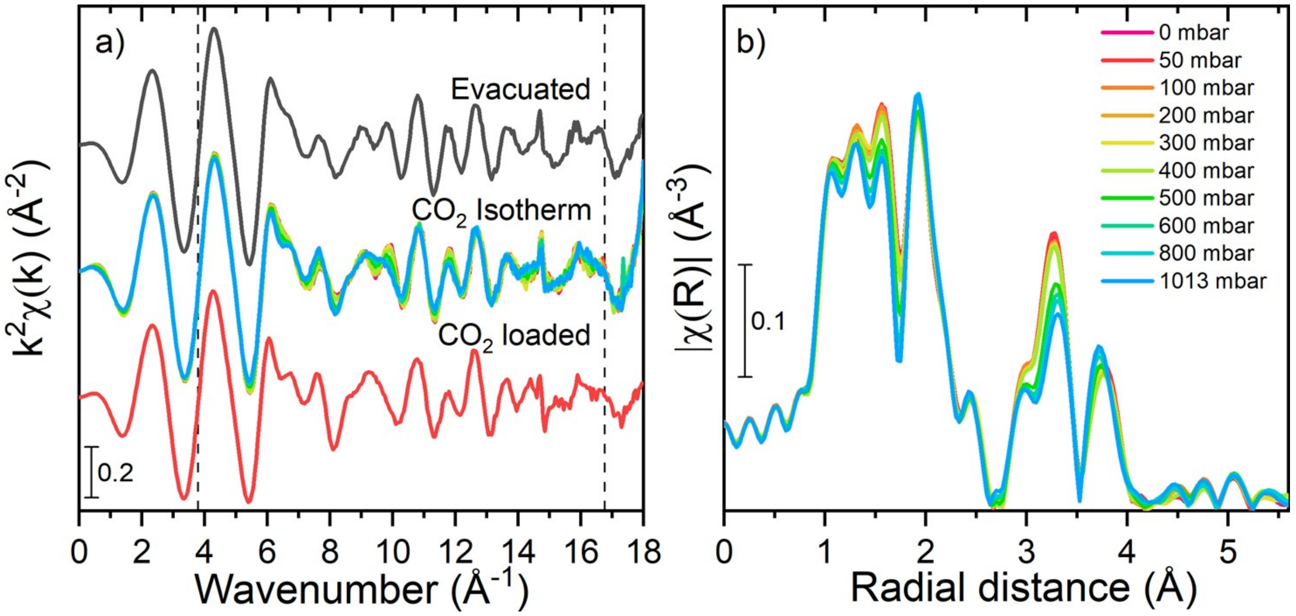

Figure S46. k^2-weighted Ce K-edge EXAFS signal (a) and the modulus of its phase-uncorrected Fourier transform (b) of F4_MIL-140A(Ce) during the CO_2 adsorption process at 328 K. Dashed lines limit the k-region used for calculating the Fourier transform.

Table S6. Relevant fitted parameters for the EXAFS fitting of F4_MIL-140A(Ce) in the as-synthesised, evacuated, and CO_2-loaded states.

Parameter	As-synthesised	Evacuated	CO_2-loaded
R-factor	0.016	0.035	0.038
Fitting range in k ($Å^{-1}$)	3.8-16.8	3.8-16.8	3.8-16.8
Fitting range in R (Å)	2.61-4.1	2.7-4.1	2.55-4.1
N_{ind}	11.89	11.13	12.39
N_{par}	9	7	9
S_0^2	0.95	0.95	0.95
ΔE	-6(3)	0(4)	-5(4)
R_{Ce1} (Å)	3.58(4)	3.56(1)	3.58(3)
σ^2_{Ce1} ($Å^2$)	0.004(4)	0.0014(8)	0.004(3)
R_{Ce2} (Å)	3.66(2)	3.66(2)	3.66(2)
σ^2_{Ce2} ($Å^2$)	0.003(2)	0.002(1)	0.004(3)
R_{Ce3} (Å)	4.05(1)	4.12(2)	4.10(2)
σ^2_{Ce3} ($Å^2$)	0.0044(4)	0.007(1)	0.0059(8)
R_{Ow} (Å)	3.368(3)	-	-
σ^2_{Ow} ($Å^2$)	0.002(2)	-	-
ΔR_{CO2} (Å)*	-	-	0.008(7)
σ^2_{CO2} ($Å^2$)	-	-	0.002(2)

*This parameter accounts as a correction factor for an isotropic elongation/contraction of all crystallographic Ce-C_{CO2} distances.

8. Solid state nuclear magnetic resonance (SSNMR)

SSNMR measurements were carried out on a Bruker Avance Neo spectrometer working at Larmor frequencies of 500.13, 470.59, and 125.77 MHz for ^{1}H, ^{19}F, and ^{13}C nuclei, respectively, equipped with a double-resonance cross-polarization magic angle spinning (CP/MAS) probe head accommodating rotors with external diameter of 4 mm.

The SSNMR characterization was performed on as-synthesised, evacuated, and CO_2-loaded F4_MIL-140A(Ce) samples. For preparing the evacuated sample, the unsealed rotor containing the as-synthesised sample was heated overnight under vacuum (0.1 mbar) at the temperature of 423 K in a home-built cell and then sealed under N_2 atmosphere. For CO_2 loading, the home-built cell containing the evacuated sample packed into the rotor was loaded with CO_2 at 1 bar pressure and the rotor was sealed under the gas atmosphere. The cell is indeed provided with a mechanical lever operated from outside enabling the capping of the rotor without disturbing the cell atmosphere.

^{1}H spectra were recorded by Direct Excitation (DE) under static conditions, accumulating 4 scans with a recycle delay between consecutive transients of 2 s. The ^{19}F DE-MAS spectra were recorded at a MAS frequency of 15 kHz using a recycle delay of 4 s and accumulating 64 scans for the evacuated and as-synthesized samples, while a recycle delay of 25 s was used and 16 scans were accumulated for the CO_2-loaded sample.

^{1}H-^{13}C and ^{19}F-^{13}C cross-polarization (CP) and heteronuclear correlation (HETCOR) experiments were performed at a MAS frequency of 15 kHz. ^{19}F-^{13}C CP-MAS spectra were recorded using contact times (*ct*) ranging from 0.2 to 10 ms using a recycle delay of 2 s for the evacuated and as-synthesized samples and of 12 s for the CO_2-loaded one; 800 scans were accumulated for all samples. The ^{1}H-^{13}C CP-MAS experiment on the as-synthesized sample was acquired using a *ct* of 2 ms, 10000 scans and a recycle delay of 2 s. For the as-synthesized and evacuated samples, ^{19}F-^{13}C HETCOR experiments with FSLG decoupling[32] in the indirect dimension were performed recording 64 rows, accumulating 80 scans with a recycle delay of 2 s and using *ct* values of 0.5, 1.5 or 3 ms. For the CO_2-loaded sample, 72 rows were acquired, accumulating 320 scans with a recycle delay of 10 s and using *ct* values of 0.2 and 2 ms.

^{13}C spectra were recorded on CO_2-loaded F4_MIL-140A(Ce) by Direct Excitation (DE) with background suppression and high power ^{19}F decoupling under both MAS (15 kHz) and static conditions. The MAS spectra were acquired with recycle delays of 150 and 2 s and accumulating 800 and 200 scans, respectively. For the static spectrum, a recycle delay of 2 s was employed to selectively observe the CO_2 signal and 25000 scans were accumulated.

All spectra were acquired at room temperature using air as spinning gas. The chemical shift for all nuclei was referenced to the ^{13}C signal of adamantane at 38.46 ppm.

The line shape of the CO_2 powder pattern in the static ^{13}C DE spectrum was simulated using the NMR-WEBLAB online software[33,34] using δ_{11}= δ_{22}= 230 ppm and δ_{33}= -85 ppm for the CO_2 principal chemical shift tensor components, in agreement with the literature,[35,36] and a Lorentzian line width of 30 ppm. The wobbling in a cone model in the fast motion limit was used with a cone angle of 75° and a flip angle of 120° (flipping between 3 sites). It must be pointed out that the same line shape could be obtained by wobbling among ≥3 sites.

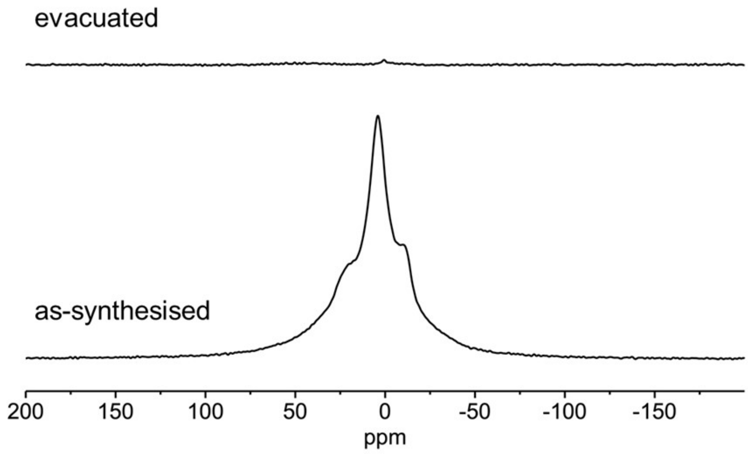

Figure S47. ^{1}H DE static spectra of as-synthesised (bottom) and evacuated (top) F4_MIL-140A(Ce).

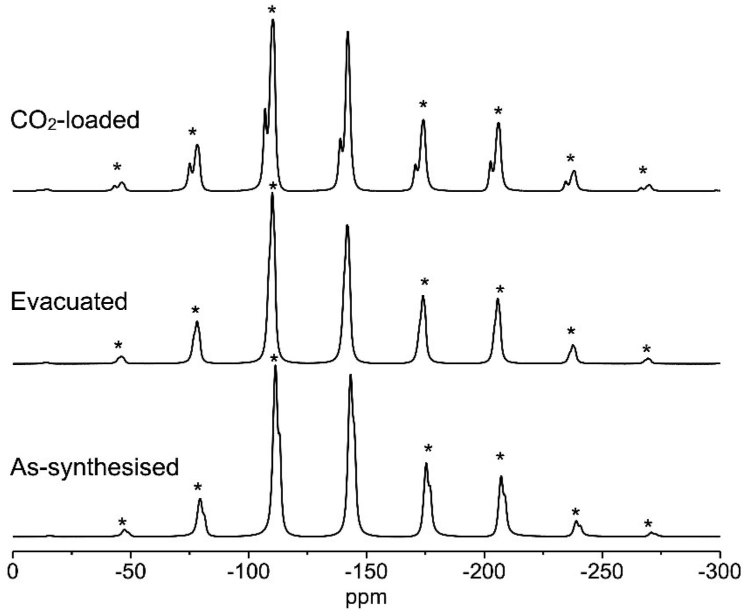

Figure S48. ^{19}F DE-MAS spectra of, from bottom to top, as-synthesised, evacuated, and CO_2-loaded F4_MIL-140A(Ce). Spinning sidebands are marked with asterisks.

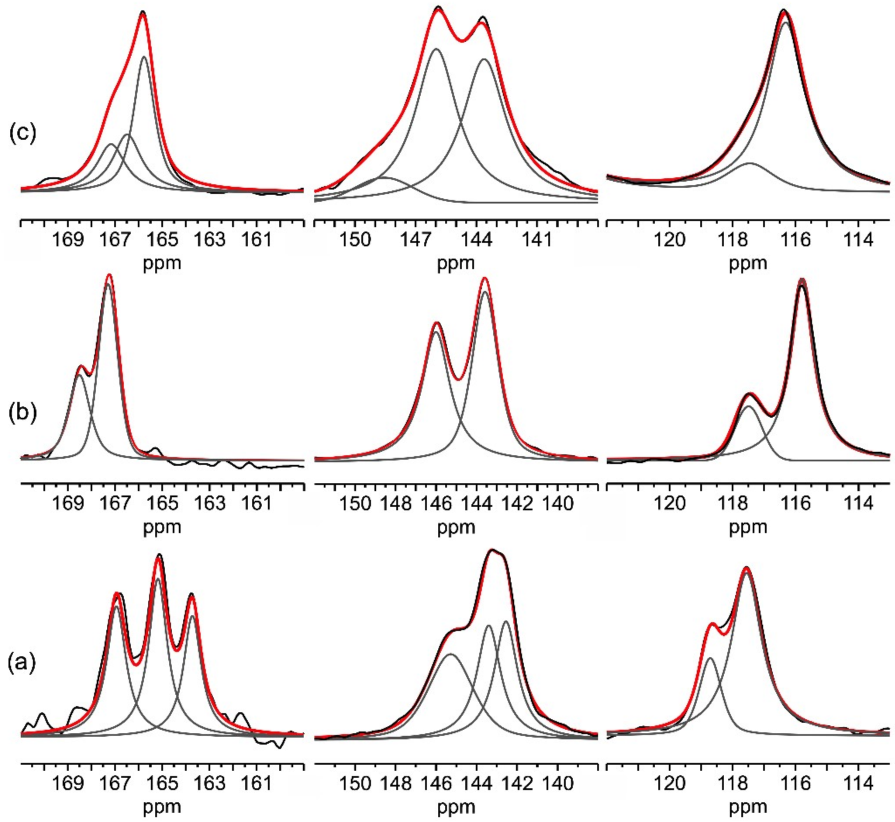

Figure S49. Spectral deconvolution of, from left to right, quaternary (Cq), fluorinated (CF), and carboxylic (COO^-) carbon signals of the ^{13}C MAS spectra of as-synthesised (a), evacuated (b), and CO_2-loaded (c) F4_MIL-140A(Ce), performed using a sum of Lorentzian-Gaussian peaks. Experimental spectra (black), calculated spectra (red) and single peaks used for deconvolution (grey) are shown. In the case of evacuated and as-synthesised samples, ^{19}F-^{13}C CP-MAS spectra recorded with *ct* = 1 ms and *ct* = 4 ms were used in the deconvolution for CF and COO^- and for Cq signals, respectively. For the CO_2-loaded sample, the deconvolution was performed on the ^{13}C DE-MAS spectrum acquired with a recycle delay of 150 s.

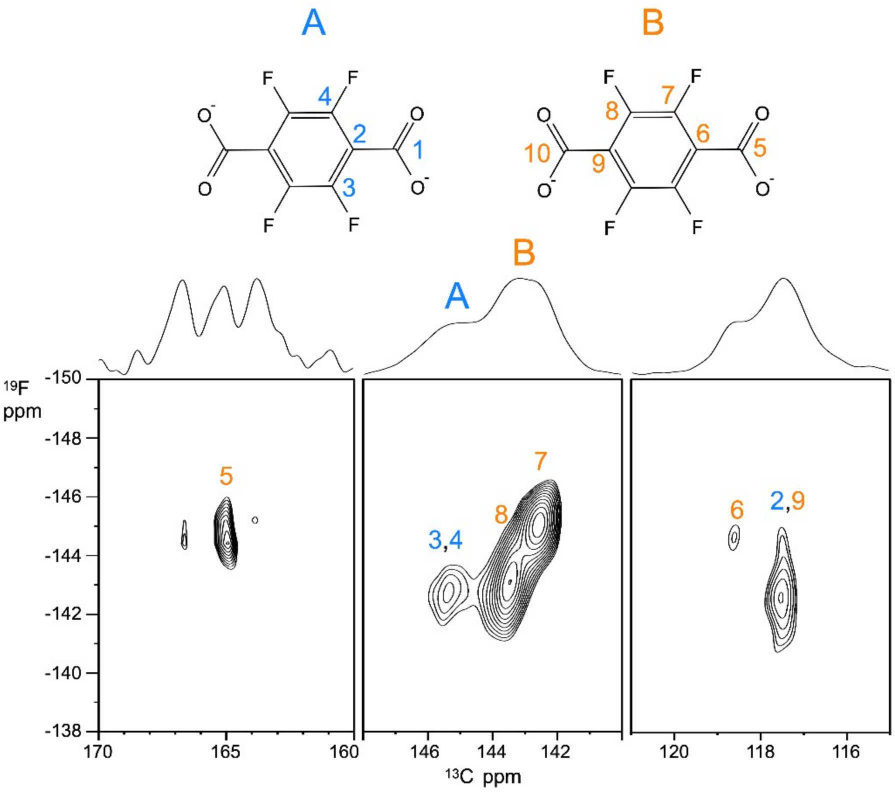

Figure S50. Expansions of the ^{19}F-^{13}C HETCOR spectra of as-synthesised F4_MIL-140A(Ce) with signal assignment. Spectral regions of carboxylic (*ct* = 1.5 ms), fluorinated (*ct* = 0.5 ms), and quaternary (*ct* = 1.5 ms) carbons are shown from left to right. A and B are the two independent linkers in the crystal structure sitting on an inversion centre and on a two-fold axis, respectively.

For as-synthesised F4_MIL-140A(Ce), the deconvolution of signals of CF, Cq and COO^- carbons in ^{19}F-^{13}C CP-MAS spectra (Figure S49) highlights the presence of three CF peaks at 142.6, 143.5 and 145.3 ppm. On the basis of the ^{19}F-^{13}C HETCOR spectra shown in Figure S50, ^{13}C signals at 142.6 and 143.5 ppm, giving strong correlation peaks with ^{19}F signals at about -145 and -143 ppm, respectively, are ascribed to CF carbons (C7 and C8) belonging to linker B. Indeed, these CF carbons seem to be bonded to two inequivalent Cq carbons (C6 and C9) resonating at different ^{13}C chemical shifts (118.7 and 117.6 ppm), as expected in the presence of a two-fold symmetry axis. Carboxylic carbons resonating at 166.9 and 165.2 ppm also belong to ring B and can be tentatively ascribed to positions C10 and C5. On the other hand, the ^{13}C signal at 145.3 ppm, correlating with the ^{19}F signal at about -143 ppm, is attributable to CF carbons (C3 and C4) belonging to linker A spatially close to Cq carbons (C2) resonating at 117.6 ppm and carboxylic carbons at 163.7 ppm (C1).

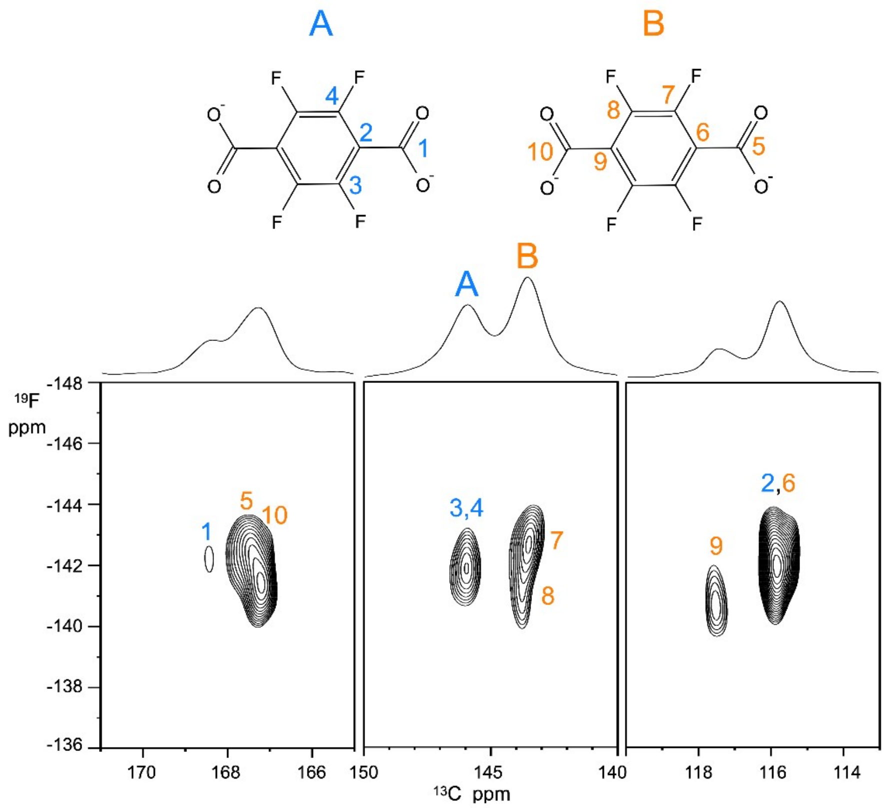

Figure S51. Expansions of the ^{19}F-^{13}C HETCOR spectra of evacuated F4_MIL-140A(Ce) with signal assignment. Spectral regions of carboxylic (*ct* = 3 ms), fluorinated (*ct* = 0.5 ms), and quaternary (*ct* = 0.5 ms) carbons are shown from left to right. A and B are the two independent linkers in the crystal structure sitting on an inversion centre and on a two-fold axis, respectively.

As far as evacuated F4_MIL-140A(Ce) is concerned, deconvolution of the ^{19}F-^{13}C CP-MAS spectra (Figure S49) highlights the presence of two CF peaks at 146.0 and 143.6 ppm, which can be ascribed to linkers A (C3 and C4) and B (C7 and C8), respectively. Indeed, based on the correlation peaks visible in the ^{19}F-^{13}C HETCOR spectrum (Figure S51), the ^{13}C peak at 143.6 ppm (C7 and C8) correlates with two distinct ^{19}F signals at about -143 and -141 ppm, which are also associated to COO^- carbons resonating at about 167.3 ppm (C5 and C10) and to two inequivalent Cq carbons resonating at 115.8 (C6) and 117.4 (C9) ppm, in agreement with the presence of a two-fold symmetry axis. On the other hand, the ^{13}C peak at 146.0 ppm (C3 and C4) correlates with the ^{19}F signal at about -142 ppm, also associated with COO^- and Cq carbons resonating at 168.4 (C1) and 115.8 (C2) ppm, respectively, which is compatible with the presence of an inversion centre in the linker.

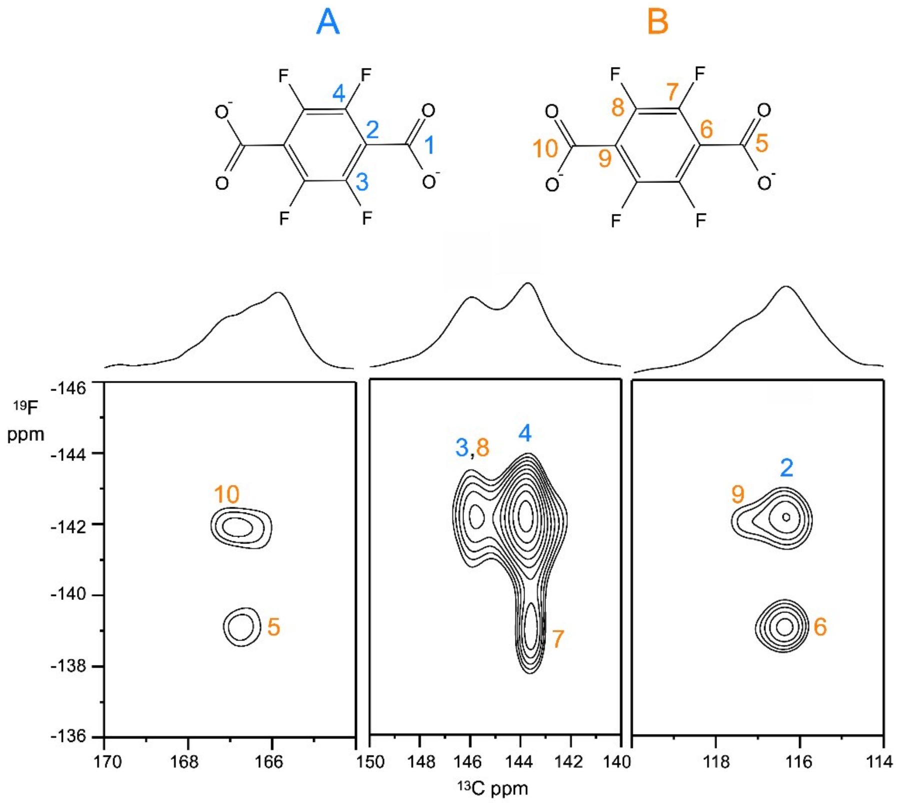

Figure S52. Expansions of the ^{19}F-^{13}C HETCOR spectra of CO_2-loaded F4_MIL-140A(Ce) with signal assignment. Spectral regions of carboxylic (*ct* = 2 ms), fluorinated (*ct* = 0.2 ms), and quaternary (*ct* = 2 ms) carbons are shown from left to right. A and B are the two independent linkers in the crystal structure sitting on an inversion centre and on a two-fold axis, respectively.

In the case of CO_2-loaded F4_MIL-140A(Ce), the deconvolution of the ^{13}C DE-MAS spectra (Figure S49) shows the presence of two CF peaks at 145.7 and 143.6 ppm, each arising from 2 CF carbons of linker A and 2 of linker B. In fact, in the ^{19}F-^{13}C HETCOR spectrum recorded with *ct* = 0.2 ms (Figure S52, central panel), in which the sole carbons bonded to fluorines are observed, the ^{13}C peak at 143.6 ppm correlates with two distinct ^{19}F signals at about -142 and -139 ppm, whereas the ^{13}C peak at 145.7 ppm only correlates with the ^{19}F signal at -142 ppm. The signals at -139 and -142 ppm indeed arise from 2 and 6 fluorine atoms, respectively. When the ^{19}F-^{13}C HETCOR spectrum is recorded with *ct* = 2 ms, a correlation peak also appears between the ^{19}F signal at -139 ppm and the ^{13}C signal at 145.7 ppm, arising from carbons not directly bonded but on the same linker. The ^{19}F signal at -139 ppm also correlates with signals of quaternary and carboxylic carbons at 116.3 ppm (3 carbons) and 166.5 ppm (1 carbon), respectively. On the other hand, the ^{19}F signal at -142 ppm correlates with quaternary carbon signals at 117.2 ppm (1 carbon) and 116.3 ppm and with COO^- signals at 167.2 ppm (1 carbon) and 165.8 ppm (2 carbons). Considering the symmetry of linkers A and B, we tentatively assign ^{13}C signals at 143.6 (C7), 116.3 (C6), and 166.5 ppm (C5), correlated to the ^{19}F signal at -139 ppm, and ^{13}C signals at 145.7 (C8), 117.2 (C9), and 167.2 ppm (C10), correlated to the ^{19}F signal at -142 ppm, to linker B. Linker A shows two different signals for fluorinated carbons at 143.6 (C4) and 145.7 (C3) ppm, although a single signal is detected for fluorine atoms at -142 ppm, and signals at 116.3 and 165.8 for C2 and C1 carbons, respectively.

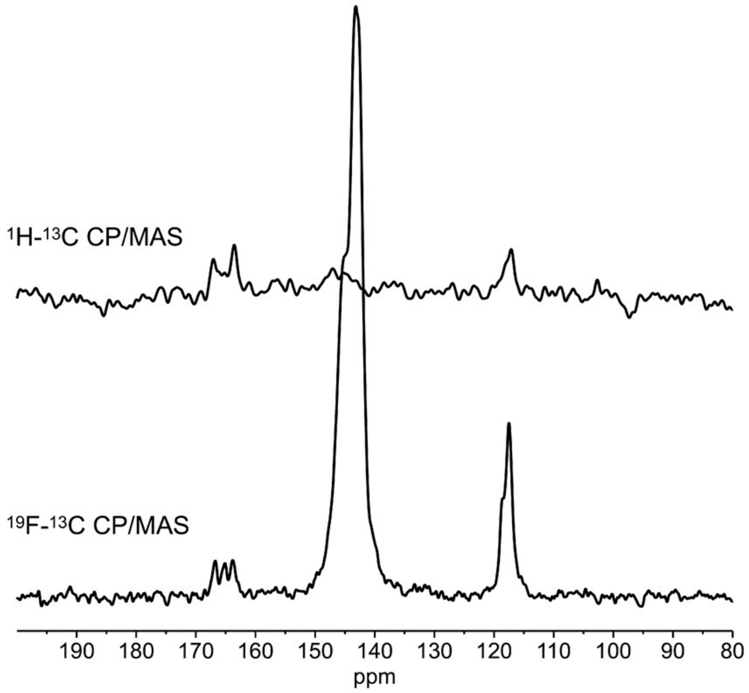

Figure S53. ^{19}F-^{13}C CP-MAS (*ct* = 1 ms, bottom) and ^{1}H-^{13}C CP-MAS (*ct* = 2 ms, top) spectra of as-synthesised F4_MIL-140A(Ce).

9. Adsorption microcalorimetry

The differential molar heat of adsorption was evaluated on F4_MIL-140A(Ce) by means of a heat flow microcalorimeter (Calvet C80 by Setaram) connected to a high-vacuum (≈ 10^{-4} mbar) glass line equipped with a Varian Ceramicell 0-100 mbar gauge and a Leybold Ceramicell 0-1000 mbar gauge. The sample was activated in vacuum at 120 °C for 12 h before being placed into the calorimeter under isothermal conditions. The measurement was performed at 303 K (30 °C) and not at 298 K (25°C) due to technical limitations. This procedure allows the determination of both integral heats evolved ($-Q_{int}$) and adsorbed amounts (N_{ads}) for small increments of the adsorptive pressure. The heats of adsorption obtained for each small dose of gas admitted over the sample ($-q_{diff}$) are reported as a function of coverage, in order to obtain the (differential) enthalpy changes. The differential heat plot was obtained by taking the middle point of the partial molar heat ($\Delta Q_{int}/\Delta N_{ads}$, kJ mol^{-1}) vs. N_a histogram relative to the individual incremental dose. A volumetric isotherm was performed at the same temperature by means of a Micromeritics ASAP 2020 instrument and were compared with the one measured at 298 K (25 °C) and 313 K (40 °C) in the pressure range 0-1.2 bar showing an intermediate CO_2 uptake (Figure S54).

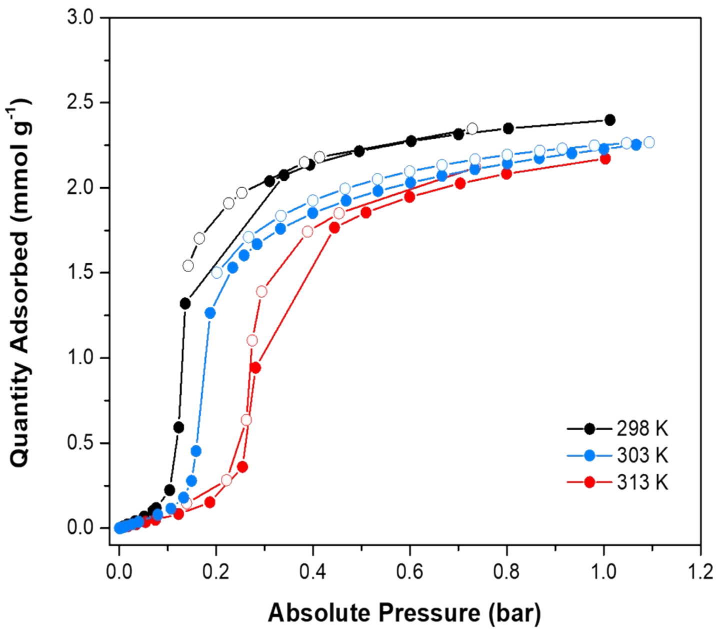

Figure S54. CO_2 adsorption-desorption isotherms collected on F4_MIL-140A(Ce) at 298 K (25 °C, black), 303 K (30 °C, light blue) and 313 K (40 °C, red) up to 1 bar.

10. Supplemental references

1 S. J. I. Shearan, J. Jacobsen, F. Costantino, R. D'Amato, D. Novikov, N. Stock, E. Andreoli and M. Taddei, *Chem. – Eur. J.*, 2021, **27**, 6579–6592.

2 Crocellà, V., Atzori, C., Latini, G., and Signorile, M.. A kit for volumetric measurements of gas adsorption. WO Pat. 2021181211A1, 2021.

3 J. Rouquerol, P. Llewellyn and F. Rouquerol, in *Studies in Surface Science and Catalysis*, eds. P. L. Llewellyn, F. Rodriquez-Reinoso, J. Rouqerol and N. Seaton, Elsevier, 2007, vol. 160, pp. 49–56.

4 F. Vindigni, M. Manzoli, T. Tabakova, V. Idakiev, F. Boccuzzi and A. Chiorino, *Phys. Chem. Chem. Phys.*, 2013, **15**, 13400–13408.

5 C. Binet, M. Daturi and J.-C. Lavalley, *Catal. Today*, 1999, **50**, 207–225.

6 S. Rojas-Buzo, P. Concepción, J. L. Olloqui-Sariego, M. Moliner and A. Corma, *ACS Appl. Mater. Interfaces*, 2021, **13**, 31021–31030.

7 R. D'Amato, A. Donnadio, M. Carta, C. Sangregorio, D. Tiana, R. Vivani, M. Taddei and F. Costantino, *ACS Sustain. Chem. Eng.*, 2019, **7**, 394–402.

8 C. Atzori, J. Ethiraj, V. Colombo, F. Bonino and S. Bordiga, *Inorganics*, *2020*, **8**, 9.

9 K. Hadjiivanov, M. Mihaylov, D. Panayotov, E. Ivanova and K. Chakarova, in *Spectroscopic Properties of Inorganic and Organometallic Compounds: Volume 45*, The Royal Society of Chemistry, 2014, vol. 45, pp. 43–78.

10 K. Chakarova, I. Strauss, M. Mihaylov, N. Drenchev and K. Hadjiivanov, *Microporous Mesoporous Mater.*, 2019, **281**, 110–122.

11 J. W. Yoon, H. Chang, S.-J. Lee, Y. K. Hwang, D.-Y. Hong, S.-K. Lee, J. S. Lee, S. Jang, T.-U. Yoon, K. Kwac, Y. Jung, R. S. Pillai, F. Faucher, A. Vimont, M. Daturi, G. Férey, C. Serre, G. Maurin, Y.-S. Bae and J.-S. Chang, *Nat. Mater.*, 2017, **16**, 526–531.

12 D. E. Jaramillo, D. A. Reed, H. Z. H. Jiang, J. Oktawiec, M. W. Mara, A. C. Forse, D. J. Lussier, R. A. Murphy, M. Cunningham, V. Colombo, D. K. Shuh, J. A. Reimer and J. R. Long, *Nat. Mater.*, 2020, **19**, 517–521.

13 A. Erba, J. Baima, I. Bush, R. Orlando and R. Dovesi, *J. Chem. Theory Comput.*, 2017, **13**, 5019–5027.

14 CRYSTAL17 manual, https://www.crystal.unito.it/Manuals/crystal17.pdf, (accessed September 2021).

15 A. D. Becke, *J. Chem. Phys.*, 1993, **98**, 1372–1377.

16 C. Lee, W. Yang and R. G. Parr, *Phys. Rev. B*, 1988, **37**, 785–789.

17 L. Valenzano, B. Civalleri, S. Chavan, S. Bordiga, M. H. Nilsen, S. Jakobsen, K. P. Lillerud and C. Lamberti, *Chem. Mater.*, 2011, **23**, 1700–1718.

18 R. Nada, C. R. A. Catlow, C. Pisani and R. Orlando, *Model. Simul. Mater. Sci. Eng.*, 1993, **1**, 165–187.

19 J. Graciani, A. M. Márquez, J. J. Plata, Y. Ortega, N. C. Hernández, A. Meyer, C. M. Zicovich-Wilson and J. Fdez. Sanz, *J. Chem. Theory Comput.*, 2011, **7**, 56–65.

20 S. Grimme, J. Antony, S. Ehrlich and H. Krieg, *J. Chem. Phys.*, 2010, **132**, 154104.

21 S. Grimme, S. Ehrlich and L. Goerigk, *J. Comput. Chem.*, 2011, **32**, 1456–1465.

22 A. Airi, C. Atzori, F. Bonino, A. Damin, S. Øien-Ødegaard, E. Aunan and S. Bordiga, *Dalton Trans.*, 2020, **49**, 12–16.

23 Z. Zhang, S. B. Peh, R. Krishna, C. Kang, K. Chai, Y. Wang, D. Shi and D. Zhao, *Angew. Chem. Int. Ed.*, 2021, **60**, 17198–17204.

24 G. Lendvay and I. Mayer, *Chem. Phys. Lett.*, 1998, **297**, 365–373.

25 A.-C. Dippel, H.-P. Liermann, J. T. Delitz, P. Walter, H. Schulte-Schrepping, O. H. Seeck and H. Franz, *J. Synchrotron Radiat.*, 2015, **22**, 675–687.

26 A. P. Hammersley, *J. Appl. Crystallogr.*, 2016, **49**, 646–652.

27 A. A. Coelho, *J. Appl. Crystallogr.*, 2018, **51**, 210–218.

28 O. Mathon, A. Beteva, J. Borrel, D. Bugnazet, S. Gatla, R. Hino, I. Kantor, T. Mairs, M. Munoz, S. Pasternak, F. Perrin and S. Pascarelli, *J. Synchrotron Radiat.*, 2015, **22**, 1548–1554.

29 K. Klementiev and R. Chernikov, *J. Phys. Conf. Ser.*, 2016, **712**, 012008.

30 B. Ravel and M. Newville, *J. Synchrotron Radiat.*, 2005, **12**, 537–541.

31 J. J. Rehr and R. C. Albers, *Rev. Mod. Phys.*, 2000, **72**, 621–654.

32 B.-J. van Rossum, H. Förster and H. J. M. de Groot, *J. Magn. Reson.*, 1997, **124**, 516–519.

33 V. Macho, L. Brombacher and H. W. Spiess, *Appl. Magn. Reson.*, 2001, **20**, 405–432.

34 https://weblab2.mpip-mainz.mpg.de/weblab67/.

35 A. J. Beeler, A. M. Orendt, D. M. Grant, P. W. Cutts, J. Michl, K. W. Zilm, J. W. Downing, J. C. Facelli, M. S. Schindler and W. Kutzelnigg, *J. Am. Chem. Soc.*, 1984, **106**, 7672–7676.

36 C. R. Bowers, H. W. Long, T. Pietrass, H. C. Gaede and A. Pines, *Chem. Phys. Lett.*, 1993, **205**, 168–170.

11. **Appendix: Coordinates of DFT-computed crystal structures**

Structures of DFT models (all in *C*2/*c* space group) are provided hereafter, according to the following format:

- **MODEL NAME**

a b c β (unit cell parameters)

N_i x_i y_i z_i (atom type N and fractional coordinates for the ith atom in the asymmetric unit)

- **F4_MIL-140A(Ce)**

25.92781344 11.52227719 8.16932396 102.348561

C 4.854167236557E-01 5.501405674093E-02 -3.975727494576E-01

C -4.046275132976E-01 7.176008820180E-02 -3.774283472749E-01

C -3.869773846949E-01 1.677206073000E-02 -4.789370091611E-01

F -3.137767528495E-01 1.469280334350E-01 -2.588544853080E-01

F 4.790296899115E-01 1.143714313349E-01 -2.961801919152E-01

C -2.692944316924E-01 3.339927772889E-02 -4.567990328479E-01

O -2.042844767242E-01 6.476525150598E-02 -3.170186013112E-01

O -2.414470829217E-01 1.588636717642E-02 4.220796373992E-01

C 3.805456668928E-01 4.640874730979E-01 1.530007450409E-01

C -4.985155360308E-01 -4.158145391792E-01 1.516183043972E-01

C 3.602032427003E-01 3.602032427003E-01 2.500000000003E-01

C -3.949203917075E-01 -3.949203917075E-01 2.500000000003E-01

F -4.804530301569E-01 -3.184770956894E-01 4.885472254510E-02

F 2.828291042087E-01 4.480952586823E-01 5.575547905287E-02

C -2.641976538740E-01 -2.641976538740E-01 2.500000000003E-01

O -1.795115528726E-01 -2.520732230624E-01 1.945089117882E-01

C 2.294054065617E-01 2.294054065617E-01 2.500000000003E-01

O 2.157634887616E-01 1.460852573728E-01 1.877743402923E-01

Ce 3.957945061952E-02 -7.790863055264E-02 1.652075088894E-01

O -1.391560183469E-02 5.366192970740E-02 -4.025621226382E-01

O 4.866673875565E-01 -1.229716184057E-01 -8.041404807361E-02

C 4.040564713190E-01 -2.145937115236E-01 -8.303532800349E-03

O 3.206876170208E-01 -3.062158820925E-01 6.283510880562E-02

- **F4_MIL-140A(Ce) + H_2O**

26.26853536 11.60362432 7.94950760 96.851517

C 2.194968993049E-01 2.496624193220E-01 -3.703039723073E-01

C -2.333047866449E-01 -1.944176265633E-01 -3.509384291046E-01

C -2.013290902954E-01 -1.920960327098E-01 -4.797262826227E-01

F -2.201549818510E-01 -1.396389566856E-01 -2.011971463734E-01

F 1.913603407172E-01 2.557007443017E-01 -2.391689777612E-01

C -1.504717146441E-01 -1.298086107017E-01 -4.580618911332E-01

O -1.293672569457E-01 -1.184571086777E-01 -3.087305423258E-01

O -1.339741651097E-01 -9.353528809936E-02 4.097327423351E-01

C -4.273055804269E-02 4.511043636248E-01 1.802152452626E-01

C -4.269512346391E-02 -4.290478215959E-01 1.808381551000E-01

C 3.469446951954E-18 3.889651613450E-01 2.500000000000E-01

C 1.734723475977E-18 -3.662103477303E-01 2.500000000000E-01

F -8.525482737377E-02 -3.738281972115E-01 1.077418640189E-01

F -8.478419039335E-02 3.950230627855E-01 1.070612952110E-01

C 1.908195823574E-17 -2.359377100649E-01 2.500000000000E-01

O 3.763795760085E-02 -1.884149369949E-01 1.944135359618E-01

C 3.469446951954E-18 2.585500858653E-01 2.500000000000E-01

O 3.116900385464E-02 2.118528219301E-01 1.629177713146E-01

Ce 5.992923877093E-02 1.371455748775E-02 1.528244888683E-01

O -3.115052489618E-02 -1.478122222499E-02 -3.918000954255E-01

O 1.152702484433E-01 -1.287605856500E-01 4.546881288344E-01

H 1.484921060215E-01 -1.427256291997E-01 -4.812182098159E-01

H 1.040279408516E-01 -2.024419379193E-01 4.057061793311E-01

- **F4_MIL-140A(Ce) + CO_2 (Ce)**

26.01471801 11.51484765 8.12522670 98.939436

C 2.109501330673E-01 2.797785871578E-01 -4.103474427406E-01

C -2.419661228959E-01 -1.608658569687E-01 -3.852750994683E-01

C -2.016787245409E-01 -1.898652815814E-01 -4.732257460320E-01

F -2.363690454279E-01 -7.292524686730E-02 -2.739002802141E-01

F 1.738873899215E-01 3.134639952617E-01 -3.213878271890E-01

C -1.506808386178E-01 -1.263409972878E-01 -4.445674834627E-01

O -1.330322863778E-01 -9.759790134366E-02 -2.971271902838E-01

O -1.296490794087E-01 -1.054589575767E-01 4.292367718520E-01

C -4.316021052791E-02 4.325691711458E-01 1.739799435183E-01

C -4.298979122494E-02 -4.466363207969E-01 1.731689267409E-01

C 3.642919299551E-17 3.703081303457E-01 2.500000000000E-01

C -3.469446951954E-17 -3.840067418356E-01 2.500000000000E-01

F -8.499642410881E-02 -3.901927475394E-01 9.192151971415E-02

F -8.547530693765E-02 3.756367359167E-01 9.615146589979E-02

C -3.469446951954E-17 -2.529596684358E-01 2.500000000000E-01

O 3.440345891876E-02 -2.046658807789E-01 1.807585143663E-01

C 1.908195823574E-17 2.395137850697E-01 2.500000000000E-01

O 3.347678714993E-02 1.913082341663E-01 1.766520840161E-01

Ce 5.940539040887E-02 -7.326184069674E-03 1.585564452665E-01

O -3.279488049221E-02 7.172398240781E-03 -3.948393291411E-01

O 1.273963925434E-01 -1.431173691964E-01 4.425017160489E-01

C 1.571955667952E-01 -2.119916977540E-01 -4.971456545962E-01

O 1.870687565381E-01 -2.797179951363E-01 -4.370297797045E-01

- **F4_MIL-140A(Ce)+ CO_2 (channel)**

25.86974494 11.54359906 8.19594208 102.459384

C 2.152013334574E-01 2.702153901983E-01 -3.975727494576E-01

C -2.381938007497E-01 -1.664337125479E-01 -3.774283472749E-01

C -2.018747227124E-01 -1.851026619824E-01 -4.789370091611E-01

F -2.303523931422E-01 -8.342435970724E-02 -2.588544853080E-01

F 1.823291292883E-01 2.967005606232E-01 -2.961801919152E-01

C -1.513468547107E-01 -1.179475769818E-01 -4.567990328479E-01

O -1.345248641151E-01 -6.975961260911E-02 -3.170186013112E-01

O -1.286667250491E-01 -1.127803578727E-01 4.220796373992E-01

C -4.177090310255E-02 4.223165699954E-01 1.530007450409E-01

C -4.135049842578E-02 -4.571650376050E-01 1.516183043972E-01

C -9.055256544599E-16 3.602032427003E-01 2.500000000003E-01

C -9.436895709314E-16 -3.949203917075E-01 2.500000000003E-01

F -8.098796723375E-02 -3.994650629232E-01 4.885472254510E-02

F -8.263307723680E-02 3.654621814455E-01 5.575547905287E-02

C -8.690964614644E-16 -2.641976538740E-01 2.500000000003E-01

O 3.628083509494E-02 -2.157923879675E-01 1.945089117882E-01

C -8.517492267046E-16 2.294054065617E-01 2.500000000003E-01

O 3.483911569438E-02 1.809243730672E-01 1.877743402923E-01

Ce 5.874404058608E-02 -1.916458996656E-02 1.652075088894E-01

O -3.378876577104E-02 1.987316393636E-02 -4.025621226382E-01

O -1.951804970189E-01 -3.181521154246E-01 -8.041404807361E-02

C -1.906749085787E-01 -4.052686201023E-01 -8.303532800349E-03

O -1.865482504434E-01 -4.927641325358E-01 6.283510880562E-02

- **F4_MIL-140A(Ce) + CO**

26.15686998 11.54955043 8.06332217 103.124707

C 2.118215259854E-01 2.866121403516E-01 -4.178834985320E-01

C -2.412291312693E-01 -1.527257050001E-01 -3.980353145445E-01

C -2.017406718539E-01 -1.882269171240E-01 -4.785310419976E-01

F -2.355668510420E-01 -5.636682798711E-02 -3.009170989714E-01

F 1.759259221817E-01 3.274666040235E-01 -3.368855474082E-01

C -1.507521306525E-01 -1.237509351451E-01 -4.523132819571E-01

O -1.341208954313E-01 -7.820526079525E-02 -3.078176269066E-01

O -1.282585686779E-01 -1.177518058346E-01 4.257541379440E-01

C -4.597878352664E-02 4.174095308874E-01 1.876866915508E-01

C -4.588863650872E-02 -4.621352880817E-01 1.860408533297E-01

C -1.912012215222E-14 3.548479421523E-01 2.500000000000E-01

C -1.915481662174E-14 -3.995183198403E-01 2.500000000000E-01

F -9.100236423786E-02 -4.064307324063E-01 1.162164089309E-01

F -9.143216995762E-02 3.617330448296E-01 1.228744510472E-01

C -1.912012215222E-14 -2.690859504632E-01 2.500000000000E-01

O 3.380568561811E-02 -2.204281832405E-01 1.846551614973E-01

C -1.912012215222E-14 2.245201096046E-01 2.500000000000E-01

O 3.463939959328E-02 1.760833230119E-01 1.884895947809E-01

Ce 5.918469792262E-02 -2.388297592973E-02 1.699559590859E-01

O -3.349439278878E-02 2.035875516396E-02 -4.038830506260E-01

C 1.422919421651E-01 1.702412785143E-01 -4.918537511768E-02

O 1.766560537945E-01 2.249210684854E-01 1.408827596079E-02

- **F4_MIL-140A(Ce) + N_2**

26.02563367 11.55006134 8.12166228 103.072579

C 2.115849380852E-01 2.856272378675E-01 -4.178987764004E-01

C -2.415102435594E-01 -1.527389221093E-01 -3.987184053956E-01

C -2.017624977206E-01 -1.872576365906E-01 -4.790983753255E-01

F -2.361044729750E-01 -5.662831662448E-02 -3.018915633004E-01

F 1.754739569596E-01 3.256181798862E-01 -3.368974529195E-01

C -1.507566149577E-01 -1.219975023453E-01 -4.535592839879E-01

O -1.342489405831E-01 -7.523886456332E-02 -3.110002252251E-01

O -1.280107196450E-01 -1.169811232899E-01 4.255500993448E-01

C -4.640839486391E-02 4.172110117491E-01 1.915409322385E-01

C -4.632074739881E-02 -4.623205593210E-01 1.897531510863E-01

C -3.816391647149E-17 3.546573808816E-01 2.500000000000E-01

C 3.469446951954E-17 -3.996268790142E-01 2.500000000000E-01

F -9.187777824700E-02 -4.067914526290E-01 1.234411401825E-01

F -9.233285201524E-02 3.615549145988E-01 1.306482451777E-01

C 1.734723475977E-17 -2.691490908365E-01 2.500000000000E-01

O 3.400424672227E-02 -2.203787805502E-01 1.852478834229E-01

C 0.000000000000E+00 2.244076344435E-01 2.500000000000E-01

O 3.455387082484E-02 1.758801878735E-01 1.877076920032E-01

Ce 5.894627481154E-02 -2.369696669647E-02 1.686988741269E-01

O -3.371121180524E-02 2.093071619295E-02 -4.036267329613E-01

N 1.401213595812E-01 -1.677603080763E-01 4.455836407849E-01

N 1.724447582064E-01 -2.190882504686E-01 -4.813790864762E-01

- **MIL-140A(Ce)**

25.71942856 11.50440493 8.14615928 105.151556

C 2.205399635092E-01 2.626525511078E-01 -3.787897372352E-01

C -2.325958360551E-01 -1.733276795950E-01 -3.632834720024E-01

C -2.030529216383E-01 -1.858838949779E-01 -4.844304307175E-01

H -2.182924380364E-01 -1.131870247415E-01 -2.588143288940E-01

H 1.970085074274E-01 2.707637869813E-01 -2.867039540399E-01

C -1.529148974192E-01 -1.165237910062E-01 -4.679849619765E-01

O -1.388406688208E-01 -4.794096695565E-02 -3.415245304802E-01

O -1.273456639942E-01 -1.283107367351E-01 4.201181597190E-01

C -4.108389262459E-02 4.131700246313E-01 2.969699511507E-01

C -4.106275287174E-02 -4.660440681313E-01 2.972517999765E-01

C 1.566975715850E-14 3.524518336548E-01 2.500000000003E-01

C 1.566975715850E-14 -4.054060646416E-01 2.500000000003E-01

H -7.198468824094E-02 -4.172876732148E-01 3.356213508561E-01

H -7.223691697051E-02 3.643918370121E-01 3.340094132487E-01

C 1.566975715850E-14 -2.757513026554E-01 2.500000000003E-01

O 3.700246840364E-02 -2.242347837883E-01 2.009941134773E-01

C 1.566975715850E-14 2.225663748674E-01 2.500000000003E-01

O 3.730865351364E-02 1.712378037947E-01 2.026187816219E-01

Ce 5.863069222246E-02 -2.854868325748E-02 1.718802482807E-01

O -3.402061084956E-02 2.705077602472E-02 -4.090981011404E-01

- **MIL-140A(Ce) + CO_2 (Ce)**

25.85726741 11.53286117 8.14388578 103.043093

C 2.178276665335E-01 2.675951102009E-01 -3.851231168505E-01

C -2.344765457965E-01 -1.726479481371E-01 -3.654907875389E-01

C -2.022123882482E-01 -1.902113997651E-01 -4.804274053947E-01

H -2.217672918467E-01 -1.113183888929E-01 -2.634732712568E-01

H 1.919968014018E-01 2.808726248635E-01 -2.989204693768E-01

C -1.511683178936E-01 -1.252280280255E-01 -4.586796288270E-01

O -1.353292410702E-01 -7.125695811758E-02 -3.208701729877E-01

O -1.269249583827E-01 -1.267834547058E-01 4.227970784118E-01

C -4.052422759012E-02 4.210436985846E-01 3.012244601244E-01

C -4.041341791364E-02 -4.585097713088E-01 3.022312875981E-01

C 1.958155859683E-14 3.602564054464E-01 2.500000000003E-01

C 1.946880157089E-14 -3.978830616381E-01 2.500000000003E-01

H -7.097153704630E-02 -4.104014943119E-01 3.444702692614E-01

H -7.157398323547E-02 3.731985189064E-01 3.407780503154E-01

C 1.946880157089E-14 -2.684996995601E-01 2.500000000003E-01

O 3.735456887865E-02 -2.171920356326E-01 2.011093456707E-01

C 1.954339468035E-14 2.305486301425E-01 2.500000000003E-01

O 3.522042583657E-02 1.794174033160E-01 1.903891860964E-01

Ce 5.894005678701E-02 -2.014638518789E-02 1.682706057479E-01

O -3.316567495893E-02 1.895981286165E-02 -4.035072143726E-01

O 1.356195896853E-01 -1.534914308486E-01 4.717304934126E-01

C 1.382210161645E-01 -2.539032039214E-01 4.751944845719E-01

O 1.418528045513E-01 -3.540326477545E-01 4.797674212758E-01
